# The gender agency gap in fiction writing (1850 to 2010)

**DOI:** 10.1073/pnas.2319514121

**Published:** 2024-07-08

**Authors:** Oscar Stuhler

**Affiliations:** ^a^Department of Sociology, Northwestern University, Evanston, IL 60208; ^b^Max Planck Institute for the Study of Societies, Cologne 50678, Germany

**Keywords:** gender, text analysis, agency, social networks, syntax

## Abstract

Works of fiction play a crucial role in the production of cultural stereotypes. Concerning gender, a widely held presumption is that many such works ascribe agency to men and passivity to women. However, large-scale diachronic analyses of this notion have been lacking. This paper provides an assessment of agency attributions in 87,531 fiction works written between 1850 and 2010. It introduces a syntax-based approach for extracting networks of character interactions. Agency is then formalized as a dyadic property: Does a character primarily serve as an agent acting upon the other character or as recipient acted upon by the other character? Findings indicate that female characters are more likely to be passive in cross-gender relationships than their male counterparts. This difference, the gender agency gap, has declined since the 19th century but persists into the 21st. Male authors are especially likely to attribute less agency to female characters. Moreover, certain kinds of actions, especially physical and villainous ones, have more pronounced gender disparities.

## Significance

Stereotypes often depict women as more passive than men, with popular culture believed to produce and reinforce such views. This study introduces a computational approach for analyzing character interactions in fiction writing. The method is used to offer a large-scale assessment of who is active and who is passive in works of fiction from 1850 to 2010. Findings show a consistent pattern: Female characters are persistently portrayed as more passive, especially by male authors. Termed “the gender agency gap”, this disparity underscores the enduring nature of gendered biases in literature, suggesting both a reflection of and potential impact on societal norms.

An old yet persistent idea about gender is that men are more agentic than women. The association of the male with the active and the female with the passive has a long arc in the history of ideas and can be found in the works of Aristotle (1), Rousseau (2), and Freud (3), among others. It was not until Beauvoir’s well-known critique of these authors (4), that this association became generally recognized as culturally produced and historically contingent. Since then, gender’s entanglement with the difference between active and passive has been a point of reflection in various branches of theoretical work on gender, including poststructuralist feminist theory (5), psychoanalytic approaches to gender (6), feminist science studies (7), and work on objectification (8). Meanwhile, social psychology has shown that a relative lack of agency is a feature of female gender stereotypes in many cultures (9–11). Works of popular culture are thought to play a crucial role in the production and dissemination of such associations. Especially in cinema, a lack of female agency has been brought into focus through the concept of the male gaze (12), which illuminates how film’s perspective is often that of a male subject on a female object. Despite the presumed pervasiveness of gendered agency disparities across different media and a depth of theoretical work, there have been no large-scale diachronic analyses of the cultural association between gender and agency.

This paper offers a comprehensive assessment of gendered agency biases in the medium of fiction writing. I extract networks of character interaction from 87,531 fiction works in order to examine the distribution of agency in literary cross-gender relationships between 1850 and 2010. While there have been prior large-scale analyses of gender in fiction writing, these have generally focused on the prevalence and the semantics of gendered characters but have not addressed the attribution of agency.

As for prevalence, it has been shown that male characters outnumber female ones and that descriptions of them take up more space on the page in 19th and 20th-century fiction writing (13–16). Consequently, interactions between male characters feature more prominently than those between female ones (17). These patterns largely prevail in 21st-century fiction (18) and are especially stark in books for children (19). A nuance to this is that the higher prevalence of male characters is at least partly a consequence of male authors’ overrepresentation in the literary field, as male authors have a stronger tendency to focus on male characters (14, 17). Furthermore, literary social structures from the late 19th to the 21st century lean toward gender heterophily, with cross-gender interaction being more prevalent than one would expect based on the prevalence of characters from both genders (17, 18).

Concerning semantics, there is evidence that descriptions of characters in English-language fiction have become less gendered over the 20th century, and that male authors tend to construct more gender role–conforming characters (13, 14). This pattern also holds for predicting gender based on interactions between male and female characters (17). Moreover, adolescent characters tend to be described in more gender role–conforming ways than adult ones, though this pattern does not change over time (15).

While neither of these findings directly speak to the question of gender and agency, literary analysis provides suggestive observations. Especially in the 19th century, descriptions of female characters are more focused on the body than those of male characters (20), which may underscore that fiction writing shares with film a tendency to represent female characters as passive objects connoting, in the words of Mulvey, “to-be-looked-at-ness” (12). Furthermore, various authors have highlighted an emphasis on female interiority in 19th-century English-language fiction (21–23) leading some to diagnose a “crisis of action” (24). Insofar as a focus on interiority is antithetical to agency, this may hint at a surplus in male agency.

More broadly, large-scale discourse analyses outside the medium of fiction provide mixed evidence on the evolution of gender stereotypes throughout the 19th and 20th centuries. Some report an overall decline in gendered associations in a variety of different text forms (25–27), while others have found no major shifts (28). Recent work using American print media finds that stereotypes about gender and agency in the context of education have remained highly stable throughout the 20th century (29). Taken together, these findings offer far from conclusive evidence for the matter at hand. We thus set out with two primary questions: Are male characters described as more agentic in fiction writing, and, if so, how has this tendency evolved since the middle of the 19th century?

## Data

To examine the distribution of agency in fiction, this study builds on the NovelTM dataset which is based on the HathiTrust Digital Library. The NovelTM aims to represent the population of fiction works held in U.S. university libraries. The corpus was assembled and is described in detail by Underwood et al. (30). The version of the corpus used here contains a total of 87,531 works of fiction written by approximately 40,000 authors between 1850 and 2010. These data are an Anglocentric representation of the literary field. While also containing works originally written in non-English languages, such works must pass the relatively high threshold of being considered both worthy of translation by a publisher and purchase by a U.S. library. Furthermore, it is worth noting that because the data reflect book-buying decisions of university libraries, they scale toward the reading preferences of a relatively well-educated, literary public. That said, to the author’s knowledge, the corpus is the largest and most comprehensive collection of English-language fiction writing that exists. Characteristics of the corpus and many other details can be found in *SI Appendix*, Section D. To provide additional evidence beyond what is presented below, all key findings are replicated with a second corpus of fiction works in *SI Appendix*, Section F.

## A Syntax-Based Measure of Agency

The measure of agency introduced in this study is theoretically grounded in formal semantics’ canonical triad of event, agent (who brings the event about), and recipient (who “undergoes” the event) (31, 32). Agency is held by an entity when it is the agent of such an event. This notion of agency is relational in that it is distributed between two textual entities—the agent, who has it in relation to the recipient, who does not. To operationalize agency, I build on syntax. Notably, I consider clauses that involve subject, verb, and object. In the clause “A kisses B”, for instance, agency is held by “A” in relation to “B.” Throughout this paper, verbs like “kiss” are referred to as actions, though it should be noted that transitive verbs connecting two characters do not exclusively connote social actions in the classic sociological sense. They may, for instance, represent cognitive processes (A believes B.) or emotions (A loves B.). Such expressions are important, for they capture pivotal perspectival facets of a narrative. Consequently, the analysis starts with an assessment of all transitive clauses, irrespective of verb semantics. Subsequently, we proceed to investigate different forms of agency, such as physical action and communication. Besides this, a valid concern is that verbs differ in their capacity to project agency onto the subject. In *SI Appendix*, Senction L, the analyses are replicated using two alternative measures of agency for which verbs are weighted with agency scores. Because both measures yield highly similar results to the ones presented below, I use the conceptually simpler measure in this paper.

To identify actions, the corpus is processed with the SpaCy language pipeline (33), which integrates a series of tasks, including word tokenization, sentence segmentation, lemmatization, part-of-speech tagging, and dependency parsing. Instances in which one textual entity is agentic toward another are identified by applying a comprehensive set of extraction rules to the annotations of this pipeline. This rule set captures simple subject–verb–object triplets like the above but also accounts for considerable syntactic variation. These variations include but are not limited to constructions that involve clausal complements (A starts to kiss B.), indirect objects (A gives B a book.), multiple subjects or objects (A asks C and B.), auxiliaries (A can kiss B.), or prepositional verbs (A talks to B.). Passive voice clauses (B is kissed by A.) are reverse coded. The scope of the rules is described in more detail in *SI Appendix*, Section A which also contains validation analyses.

For coreference resolution, the task of identifying all textual features referring to the same character, I build on the BookNLP pipeline (34, 35) that has been optimized for performing this task in English fiction. The pipeline also generates predictions for the gender of each character (see *SI Appendix*, Sections M and K for validation and supplementary analyses) based on multiple pieces of information, including the gender scaling of given names according to government records, the alignment of character names with gendered honorifics (e.g., “Mr. Pargiter”), as well as pronoun associations obtained through coreference (i.e., characters referenced with male or female pronouns are tagged as male or female, respectively). Given this, it should be clear that the term gender is used in this manuscript to connote referential gender. More complex notions of gender will likely require a more sophisticated measurement approach.

**Fig. 1.**
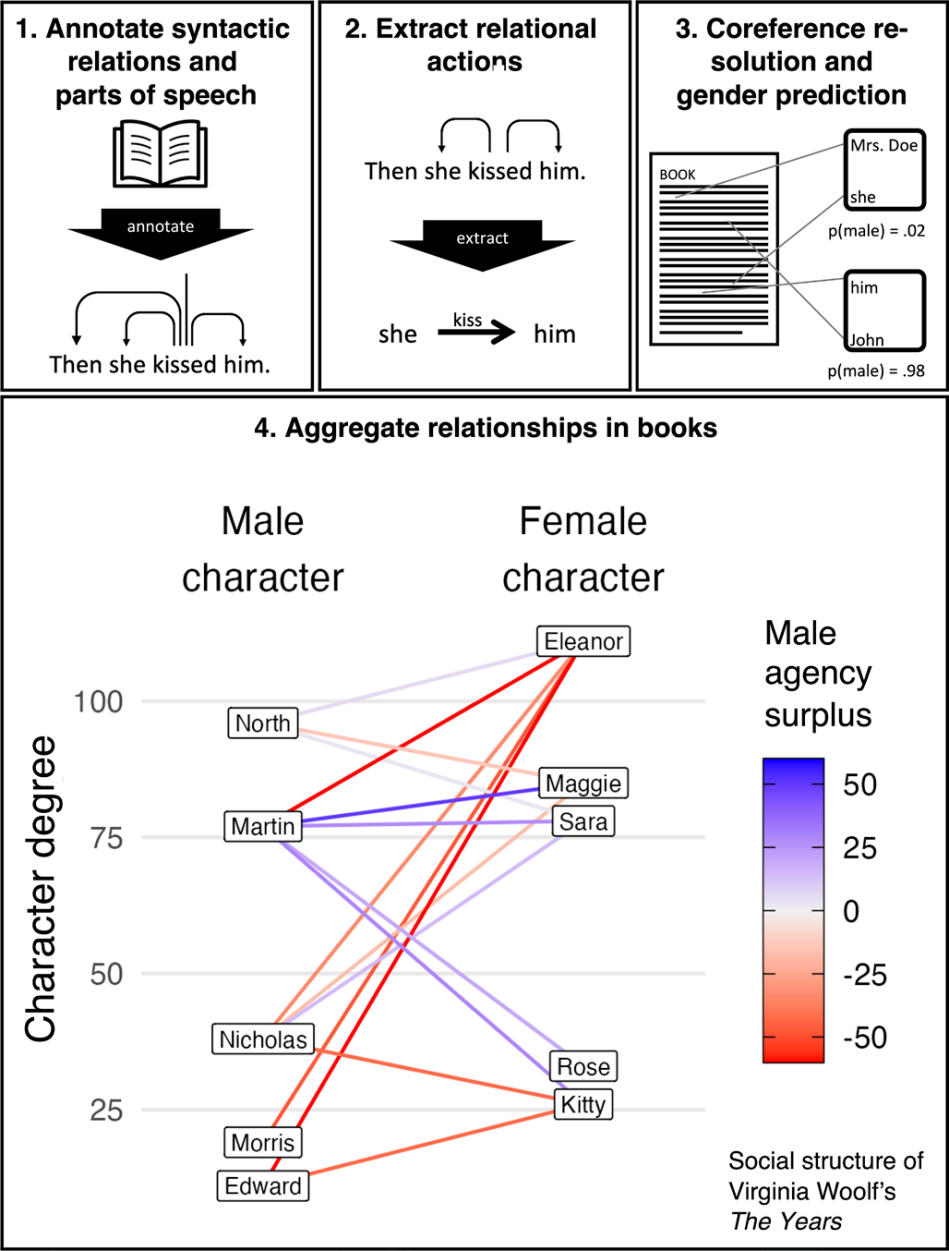
Workflow for extracting networks of character interactions from text. The first three panels are schematic representations of steps underlying the extraction of character interactions. Panel 4 shows the relationships between the five most important male and female characters in Virginia Woolf’s The Years. Importance is measured via a character’s degree, that is, the total number of actions the character is involved in within all relationships that pass a threshold of at least five exchanged actions, including same-gender ones. Characters were labeled with the most frequent proper noun referring to them.

Finally, the data are aggregated at the level of relationships between characters. Each relationship comprises two sets of actions, those sent from A to B and those sent from B to A. For the subsequent analyses, only the main relationships in a book are considered, defined as those passing a threshold of at least five exchanged actions. The criterion ensures a focus on relationships between recurrent characters for whom gender can be reliably predicted (see *SI Appendix*, Section K for supplementary analyses with alternative specifications). This yields 568,302 cross-gender relationships between 333,330 male and 297,249 female characters, with an average of 14.8 actions per relationship. For each dyad, the percentage point surplus of male agency is then measured. To illustrate this, consider Panel 4 of Fig. 1 which shows the distribution of agency among the five most important male and female characters in Virginia Woolf’s The Years. Importance is measured via a character’s degree, that is, the total number of actions the character is involved in with other characters. The relationship between Eleanor, the lead character of the novel, and her brother Martin is composed of 10 exchanged actions, with Eleanor sending 80% of the actions, and Martin only sending 20%, resulting in a value of −60 for the measure of male agency surplus. The measure ranges from −100 (all actions were sent by the female character) to 100 (all actions were sent by the male character).

## Results

**Fig. 2.**
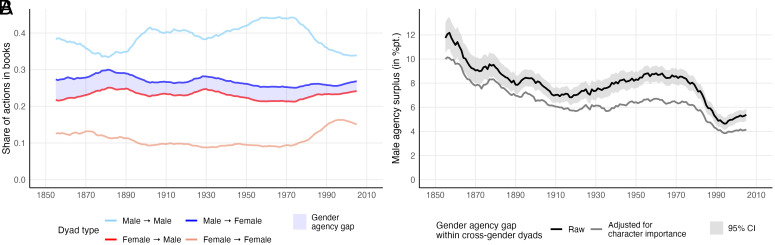
Gender distribution of actions and gender agency gap over time. Panel (*A*) shows the share of actions in the main relationships with more than five exchanged actions. Shares were averaged over books. Panel (*B*) shows the gender agency gap over time (black). The gray line represents the adjusted gap, that is, the expected gap for a scenario in which male and female characters were equally important to each work according to their degree, effective ego network size, and betweenness centrality. Estimates are based on linear mixed models described in more detail in *SI Appendix*, Section B. Lines represent 10-y moving averages. For the importance-adjusted gap, this means that an independent model was fit for every 10-y window. Note that due to measurement error in gender prediction, these numbers provide a conservative estimate of the gender agency gap. I estimate that the true gap may be around 0.8 (raw) to 0.7 (importance-adjusted) percentage points higher than presented in Panel (*B*). Analyses and discussions of this are provided in *SI Appendix*, Sections K and M.

The analyses begin with an examination of the distribution of action over the four possible dyad types in the main relationships of all works (Fig. 2*A*). On average, actions in male → male relationships make up 38.9% of the actions in a book. This is more than three times as much as actions in female → female relationships at 11.4%. Thus, a by-product of this analysis is comprehensive evidence to suggest that substantive relationships among recurrent female characters are severely underrepresented in fiction writing, a quality that has primarily been discussed for the medium of film (36, also see *SI Appendix*, Section O for further contextualization and discussion of this finding). However, this does not tell much about the distribution of agency as a relational quality, for which actions in cross-gender relationships must be considered. Here, we find that male → female actions make up 26.6% of the average book’s actions, while female → male ones make up only 23.1%. This implies an average gender agency gap of 7.2 percentage points, with male and female characters being responsible for 53.6% and 46.4% of the actions in cross-gender relationships, respectively. Put differently, for every six female → male actions in a work of fiction, there are about seven male → female actions.

Research has shown that representations of gender in literary works have generally become less stereotypical since the late 19th century (14, 17). The gender agency gap persists throughout the entire period between 1850 and 2010 but has nearly halved over this time (Fig. 2*B*). Its decline has not been linear, however. Instead, it features two relatively steep drops as well as long phases of relative stagnation and backslide. The first drop occurred in the second half of the 19th century. This was followed by a backlash that lasted for much of the 20th century and roughly coincides with the Fordist period in most Western societies. The 1950s and 60s, generally recognized as a period with rigid gender roles in dominant U.S. culture (37), were the 20th century’s decades that saw the lowest levels of female agency. It was only in the 1970s, that female characters started to quickly gain agency, eventually reducing the gap to around 5 percentage points around 1990—the lowest it has been for the period studied. While precise chronological limits of societal trends are hard to define, it is noteworthy that this second drop occurred in a period generally associated with second-wave feminism. Since 1990, the gap stabilized again, aligning with the relative stagnation of many indicators for gender equality in the United States (38).

### Adjusting for Character Importance.

These results establish that in cross-gender relationships, male characters are typically more agentic than female ones. In this regard, however, it is worth noting an insight from the sociology of gender: Many of the asymmetries found in real-world male–female interaction are not a direct consequence of gender, but rather of the fact that they occur in contexts in which men tend to have higher status (39, 40). Instead of being a consequence of gender per se, for instance, observed differences in male and female social behavior at work may result from men holding more senior positions. When men and women occupy the same structural positions, many behavioral differences become considerably less pronounced (41, 42). While these findings may not be directly convertible to the realm of fiction, there is an analogous structural inequality in the sense that fiction tends to focus on male characters (13–16, 18, also see Fig. 2*A*). It seems plausible that characters that are more central to the plot are portrayed as more agentic in their relationships with less central ones. Consider, for instance, Virginia Woolf’s The Years, where in most relationships, the character with higher degree carries more agency (Panel 4 of Fig. 1). This prompts the following question: How much of the agency gap can be attributed to the fact that male characters are, on average, featured more centrally in works of fiction?

While character importance is an established concept of literary analysis (43), there is no agreed-upon formal measurement. Building on recent work that has highlighted the potential of network-based measures for this purpose (44–46), I operationalize the extent to which a work focuses on a character with three conceptually distinct measures. First, I measure character degree, defined as the number of actions a character is involved in within all its relationships. The intuition here is that the amount of attention a work devotes to a character’s social conduct reflects its importance. Second, I use the effective size of a character’s ego network. This measure was proposed as an operationalization of the notion of “structural holes” (47, 48) and takes into account that a node may be structurally redundant to the extent that its contacts are connected to one another. In the case at hand, the measure implements the notion that a character is more important if it is positioned in a structural hole of the character network, that is, the character serves as a bridge connecting different relatively unconnected parts of the story’s social structure. Finally, I use the betweenness centrality of a character. Unlike the former, this measure is based on the global network and implements the idea that a character is more relevant if it is placed at the center of a book’s entire social structure. On average, male characters are indeed more important according to each of these measures. All attributes are then used to create three measures for the relative difference in character importance within a given dyad (see *SI Appendix*, Section N for details and formal definitions).

Subsequently, these dyadic measures are used as covariates in a linear mixed model that predicts the male agency advantage in a dyad and includes random intercepts for male and female characters, books, and authors. The different forms of character importance are highly correlated and each of them is associated with having more agency. Yet, a combined model indicates that it is especially a character’s capacity to bridge structural holes in the character network that comes with agency advantages (see detailed results in *SI Appendix*, Section B). A one SD increase in the corresponding dyadic measure is estimated to lead to a 9.6 percentage point higher agency share (*P* < 0.001). In this sense, agency appears more tightly linked to a character’s structural position than to the attention given to its conduct. To assess the extent to which fiction’s focus on male characters contributes to male agency advantages, I fit independent models for 10-y time windows and use the their parameters to estimate the expected gap for a counterfactual scenario in which male and female characters were equally important according to each of the three measures. The results are presented in Fig. 2*B*. Averaging over the time series, 18.7% of the raw gender agency gap is accounted for by male characters’ greater importance. This implies that even in relationships where the male and the female characters are equally important to the book’s plot, the latter usually holds more agency. In other words, male agency advantages are not primarily a by-product of fiction’s previously documented tendency to focus on male characters, but a largely independent quality of literary cross-gender relationships.

### Author Gender.

Beyond text-immanent factors, another possible explanation for the gender agency gap lies in the underrepresentation of female authors in the literary field. An estimated 66.7% of the books in the corpus were written by men (see *SI Appendix*, Section H on author gender inference). If male and female authors were to attribute more agency to characters of their own gender, the gender agency gap could be a consequence of male authors numerically dominating the discourse. There is some prior research hinting in this direction. Traditionally, literary critics have pointed to female authors’ unique capacity to transcend conventions of a patriarchic canon, though also at times to the unique conformity pressures faced especially by 19th-century women authors (49–51). Recent work in the digital humanities shows that female authors tend to describe characters in less gender role–conforming ways (13, 14, 16).

**Fig. 3.**
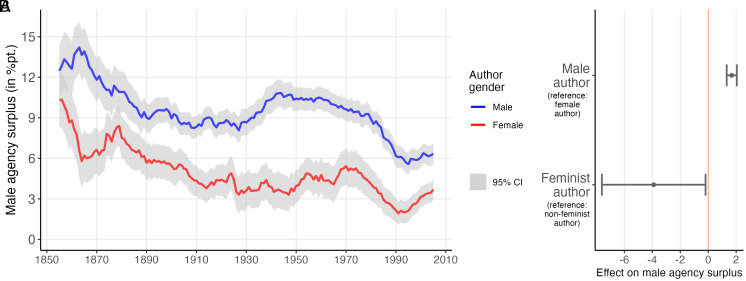
Gender agency gap and author gender. Panel (*A*) shows the gender agency gap over time by author gender. Lines represent 10-y moving averages with 95% CIs. Panel (*B*) shows the effect of author gender and of the author being identified as a feminist on a cross-gender dyads agency imbalance. These are based on a linear mixed model fit with the 486,180 dyads contained in works with identified author gender. The model uses random intercepts for male and female characters, books, and authors, as well as fixed effects for each decade; it controls for degree imbalances between characters and the number of actions exchanged in a dyad (see *SI Appendix*, Section B for details). Bars denote 95% CIs based on cluster-robust SEs on the author level.

Fig. 3*A* shows that both male and female authors attribute more agency to male characters. However, the gap in male-authored works is notably larger, in some periods, around three times that of female-authored works. Trends in the works of male and female writers mostly align; however, especially the midcentury backlash was more pronounced in male-authored works. Some portion of the discrepancy between male and female authors can be attributed to the fact that male authors feature male characters more prominently. Yet even when controlling for differences in character importance via the three measures established in the previous section, the agency gap is estimated to be 1.7 percentage points higher in male-authored works (Fig. 3*B*). To further nuance this finding, I tested whether authors regarded as feminists attribute more agency to female characters. Such authors were identified by consulting various curated recommendation lists (*SI Appendix*, Section E). This indeed appears to be the case, as the average gender agency gap among feminist authors is 0.6 percentage points—suggesting almost equal agency between male and female characters. It should be noted, however, that the effect in the model is barely statistically significant as there is considerable heterogeneity among feminist writers (see *SI Appendix*, Section E for supplementary analyses). In sum, the gender agency gap persists irrespective of the author’s gender. However, female and especially feminist authors tend to distribute agency more equally when writing about cross-gender relationships.

### Actions’ Gender Valences.

**Fig. 4.**
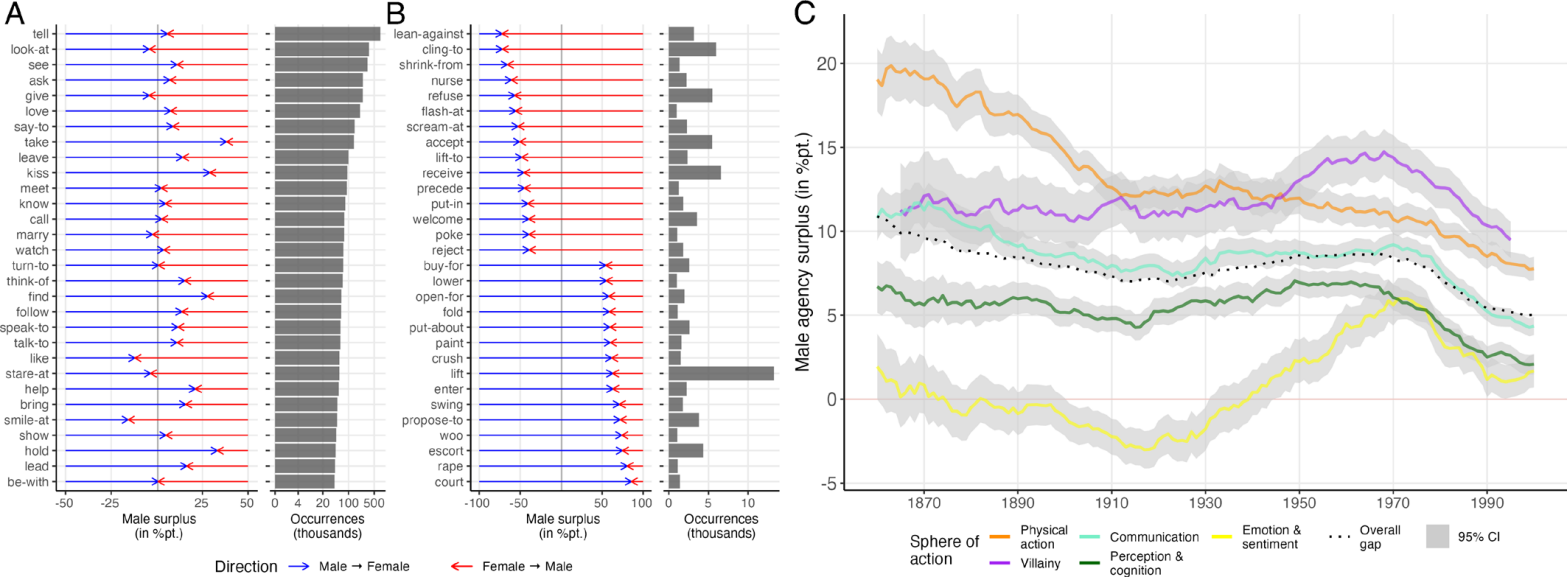
Actions’ gender valence and male agency surplus by sphere of action. Panels (*A*) and (*B*) show the gender valence of the most frequent (Panel *A*) and the most imbalanced (Panel *B*) actions as they occur in cross-gender relationships. The arrows indicate the share of instantiations of the respective action that have a male (blue) or female (red) subject, the object being of the other gender, respectively. For Panel (*B*), a frequency threshold of at least 1,000 occurrences was used. Panel (*C*) shows the male agency surplus for six different action spheres with 90% CIs based on 20-y moving averages (30-y for villainy as the data are more sparse). The measures are conceptually identical to those shown in Fig. 2*B* and Fig. 3*A*, except that only actions associated with the respective sphere were considered. For details on sphere-specific verb dictionaries, see *SI Appendix*, Section I.

Thus far, we have examined the distribution of agency at large. However, there is good reason to believe that different kinds of action come with different distributions. Gender as a social construct, that is, as something which must be enacted, is premised on certain actions being culturally encoded as male or female. Yet while certain behaviors are recognized as male or female and research has concerned itself with how gender is performed in interaction (52, 53), formally measured evidence for the gender valence of specific actions is hard to come by. For instance, the neologism “mansplain”, has entered the cultural mainstream in the United States to highlight and mock that men often feel the need to explain things to women. But is the action of explaining something to someone actually a typically male → female one? Here, we have the unique opportunity to assess the gendered distribution of actions. When considering all 18,518 instances of “explain-to” that occurred across gender lines, we indeed find that 61.0% of them were initiated by male subjects. Fig. 4 shows the gender valence of the most frequent action in cross-gender relationships (Panel *A*) as well as those with the strongest imbalance in either direction (Panel *B*). Most of the highly frequent actions are conducted by characters of both genders, though there are exceptions as some physical actions lean toward male agency (“kiss”, “hold”), while others are associated with female agency (“smile-at”). Among the most masculine forms of agency are pursuit and courtship (“court”, “woo”, “propose-to”, “buy-for”) as well as actions indicative of physical strength (“swing”, “lift”, “fold”). Inversely, some of the most stereotypically female actions are responses to male advances (“refuse”, “accept”, “reject”) or ones that claim physical support (“lean-against”, “cling-to”). As a general resource for research on the evolution of gender roles, a longitudinal dataset with the estimated gender valences of all actions is made available as a supplement to this paper (also see *SI Appendix*, Section C for a web interface which allows for easy exploration of the data).

### Gendered Spheres of Action.

In the present paper, I study these distributions of agency more systematically by building on the notion of spheres of action, originated by Russian formalist Vladimir Propp. In a classic study, Propp (54) observed that folktales are typically structured around archetypical roles engaged in certain kinds of actions. There is, for instance, the sphere of action of the villain, constituted by acts of villainy. While modern and contemporary literature is more complex and defies Propp’s typology, conventionalized pairings of character roles and particular forms of action persist and are often intertwined with gender. Take, for instance, the archetype of the male hero in adventure fiction, who is primarily engaged in physical actions but is often uncommunicative and lacks complex emotions toward others (55, 56). In contrast, female protagonists’ thoughts and emotions are at the center of 19th-century domestic fiction (16, 21, 22). Here, five such spheres of action are examined more closely: physical action (e.g., touch, push, kiss); communication (e.g., tell, ask, answer); perception and cognition (e.g., watch, believe, remember); emotion and sentiment (e.g., love, hate, miss); as well as villainy (e.g., kill, abuse, harm). To measure these, action dictionaries for each dimension were created in a two-step process that involved assigning the most frequent verbs to dictionaries and subsequently expanding these with a generative language model. The distributions of sphere-specific agency for each dyad are then computed by considering only the actions contained in the respective dictionary. Details on these measures and additional analyses are provided in *SI Appendix*, Section I.

Fig. 4*C* shows the distribution of agency in different spheres of action over time. The gap between male and female characters is particularly large for physical action. In the mid-19th century, male characters had an advantage of up to 19 percentage points, implying, roughly, that for every two physical actions by a female character in a cross-gender relationship, a male character conducts three. Similarly, villainy is in the domain of male agency, with female characters typically being the recipients of such actions. Remarkable here is the lack of change. While most other action spheres—like action itself—become more equally distributed over time, villainous actions are almost as male at the turn of the 21st century as in the mid-19th century. The pattern for communication closely follows that of the overall agency gap, while the imbalance in perception and cognition is typically 3 to 4 percentage points lower.

Emotions are the only sphere with a notably different distribution and trend. Here, female characters had approximately equal or even slightly more agency well into the 20th century. Only from the 1940s onward did the distribution start to favor male characters. Instead of male characters becoming more emotional, however, supplementary analyses (*SI Appendix*, Section I) indicate that the prevalence of emotions in cross-gender relationships has steeply declined overall since the 19th century, but that this trend was more pronounced and persistent for female characters. That said, it should be noted that while there has been a surplus in male emotions since the 1940s, this surplus is lower than the overall gap, implying that emotions still make up for a larger share of female characters’ actions than of male characters’ actions.

Overall, I find that beneath the aggregate trend, there lies considerable heterogeneity in how agency is distributed in different spheres of action. For instance, while male characters continue to be responsible for most of the physical action in cross-gender relationships in the 21st century, agency is almost equally distributed in the spheres of emotion or perception. Whereas differences between spheres were stark before the midcentury, there is an overall trend toward convergence, which suggests a general decline in the gendering of forms of agency.

### Agency and Gender Role Conformity.

The previous analyses show that agency is unequally distributed among characters of different genders, but also that there are differences in the kinds of actions that male and female characters direct toward each other. What is the relationship between these two modes of constructing gender; specifically, what is the relationship between agency, defined as the relational quality of who initiates more action, and the genderedness of action content? To examine this, I form a measure for the gender role conformity^*^ of a dyad. This measure is based on a simple intuition: How predictable is the direction of the actions contained in a cross-gender dyad, given what we know about the genderedness of particular actions?

To operationalize this notion, I first compute the conditional probabilities of all actions *a* for both directions based on the content of all cross-gender dyads. For instance, for the action “nursing”, I generate the probabilities *p(a = nurse | m → f)* and *p(a = nurse | f → m)*. For each action, I then use these two probabilities to form the terms *p(m → f | a)* and *p(f → m | a)*. For the case of “nursing”, for instance, *p(m → f)* = 0.18 and *p(f → m)* = 0.82. Note that because I base these terms on actions’ probabilities conditional on direction, they are independent of the fact that there are more male → female than female → male actions. The role conformity of a dyad is then defined as the average likelihood of its actions’ observed directions (see *SI Appendix*, Section Q for details). Values closer to 0 indicate that a dyad’s action content is subversive and unexpected under the prevailing gender roles. For instance, a cross-gender relationship in which the male character “nurses” and “smiles-at” the female character while the latter “explains-to”, “buys-for”, and “woos” the male character has a value of 0.27. Meanwhile, values closer to 1 indicate gender role conformity and a cross-gender relationship in which the previously mentioned actions go in the other direction would have a value of 0.73.

I then test whether the role conformity of conduct in a relationship is predictive of male agency advantages, using the new measure as an additional covariate in the linear mixed model described above. When controlling for variation over time as well as all other variables previously discussed, I find that a one-SD increase in the gender role conformity of a dyad’s action content is associated with a 5.7 percentage point increase in the male agency surplus (*P* < 0.001; models are documented in *SI Appendix*, Section Q). In other words, in relationships in which the characters engage in gender role–conforming conduct, female characters typically also have less agency. This suggests that presenting male characters as active and female characters as passive is but one way that authors construct gender, one that usually coincides with other modes of constructing gendered identities in fiction writing.

## Discussion

More so than other media, works of fiction do not only document but broadcast social norms. They help define ideas about what conduct is appropriate for different kinds of identities. Crucially, this includes shaping societal expectations about what men and women behave like. This paper reveals a persistent gender agency gap in fiction writing. Specifically, we find that female characters are systematically presented as more passive than male characters in cross-gender relationships. This disparity declined since the 19th century but prevails into the 21st. Male writers are especially likely to attribute passivity to female characters. Moreover, it is shown that some forms of agency are more heavily gendered than others, with physical action and villainy being firmly in the domain of male agency, while emotions are more equally distributed between characters of both genders.

Beyond these primary findings, the present study points to various avenues for further research. As much of social science strives to move beyond proof-of-concept uses of natural language processing (57), a central challenge for the computational study of narrative (58, 59) is to find ways of breaking down text into features that align with the analytical concepts developed by humanists and sociologists. The present study contributes to this effort by introducing an approach to extracting networks of character interaction from textual data. This opens up a variety of possibilities for future inquiry.

One of them is a general assessment of economies of agency in works of popular culture. While the present study is a first step in this direction, it remains limited by its focus on gender in the absence of other sociodemographic traits like race, class, or age, let alone their intersection. This has to do with methodological constraints. The linguistic manifestation of gender through pronouns, honorifics, and given names, allows for reliable prediction of entities’ referential gender. Other sociodemographic characteristics are considerably harder to infer from text as they often remain implicit and name-based inference is less robust (60). Developing estimation techniques for the salience of such traits on the entity level will be crucial to studying economies of agency and, more broadly, to advancing the emerging study of intersectionality with natural language processing methods (61, 62). Besides this, this paper focuses on agency as a quality of social relationships. However, there are other ways in which character agency can manifest itself, such as a character’s capacity to drive its own storyline. Future work could aim to examine different modes of agency, as well as their interrelatedness.

In more general terms, the present study demonstrates how attributions of agency can be formally measured at scale. Future work may exploit this to examine such attributions in discursive domains that are more directly intertwined with real-world social structures. This can involve, for instance, studying how public discourse attributes agency to market identities (63), social categories (64), or political actors. Meanwhile, research aiming to uncover how social relationships are constructed and altered through communicative efforts (65, 66) may examine how individuals claim or strategically attribute agency in conversation or written interpersonal communication. Beyond the domain of fiction, the present study thus points to the possibility of a syntactically grounded computational study of agency attributions.

## Materials, Methods, and Supplementary Analyses

### Text Corpus.

Details on corpus composition and descriptive statistics are provided in *SI Appendix*, Section D. *SI Appendix*, Section F contains a replication of the main analyses using an alternative corpus.

### Models.

Regression models are documented and described in more detail in *SI Appendix*, Section B. *SI Appendix*, Section Q contains the formal definition of the role conformity measure and the models estimating its effect on the male agency surplus.

### Alternative Measures and Specifications.

Replications of the analyses with two alternative measures of agency are provided in *SI Appendix*, Section L. *SI Appendix*, Sections J, K, and M contain supplementary analyses and analyses with alternative specifications for which characters and relationships should be considered. *SI Appendix*, Section P explores whether the gender agency gap typically changes or declines over the course of a book.

### Validation and Explanation of Measures.

Details on action extraction as well as corresponding validation analyses are provided in *SI Appendix*, Section A. Validation of character gender prediction and coreference resolution can be found in *SI Appendix*, Section M. *SI Appendix*, Section K additionally investigates the potential implications of measurement error in gender prediction for the estimation of the size of the gender agency gap. Details on the identification of feminist authors are provided in *SI Appendix*, Section E. The process of generating dictionaries for spheres of action, as well as the dictionaries themselves are detailed in *SI Appendix*, Section I. Inference for authors’ gender is explained and validated in *SI Appendix*, Section H. *SI Appendix*, Section N contains additional information for the measures of character importance. *SI Appendix*, Section O examines the distribution of reference forms by character gender.

## Supplementary Material

Appendix 01 (PDF)

## Data Availability

Code used for this study and aggregate data of actions’ gender valences data have been deposited in OSF (https://osf.io/64cwz/) (67). The text data contained in the NovelTM that were used for this study are copyrighted and cannot be made available by the author. However, the full texts are generally available to researchers through the HathiTrust Digital Library (https://www.hathitrust.org/) (30). Additional details on corpus creation are provided in a previously published paper (30).

## Footnotes

^*^I use the term “role” here to highlight how expectations link gendered identities to conduct, without intending to evoke the functionalist connotations at times associated with this term.
