# Supplementary material for: The gender agency gap in fiction writing (1850 to 2010)
Source: Proc Natl Acad Sci U S A. 2024 Jul 8;121(29):e2319514121. doi: 10.1073/pnas.2319514121 (PMC11260095; doi:10.1073/pnas.2319514121)
Supplement: Supplementary file 1 — Appendix 01 (PDF) [file pnas.2319514121.sapp.pdf]

# Supporting Information for

## The gender agency gap in fiction writing (1850-2010)

**Oscar Stuhler**

**Correspondence should be sent to**
**E-mail: oms@northwestern.edu**

**This PDF file includes:**

Supporting text
Figs. S1 to S26
Tables S1 to S14
SI References

## Supporting Information Text

This document contains additional details on methods and data as well as supplementary analyses with alternative data and measures.

## Contents


A Extracting directed actions 3

B Regression models 8

C Action data and web interface 10

D Notes on corpus composition and descriptive statistics 11

E Identifying feminist authors 15

F Replication of main findings with US Novel Corpus 18

G Genre analyses 20

H Identifying author gender 21

I Spheres of action 23

J Analyses without first-person narrator 27

K Analyses based on variations of the action threshold 29

L Using weighted verb representations for alternative measures of agency 32

M Validation of coreference resolution and character gender prediction 36

N Measures for character importance 39

O Distribution of reference form by character gender 41

P Within relationship change of gender agency gap 42

Q Gender role conformity within dyads 43

## A. Extracting directed actions

To identify actions directed from one character towards another, I employ a combination of dependency parsing and rule-based extraction. Dependency parsers are models that predict the syntactic relationships between the lexical features in a sentence. Syntactic dependencies can be indicative of semantic relationships. To illustrate this, consider Figure A 1, which shows a sentence with dependency annotations and part-of-speech tags. The token *Jane* is labeled as the nominal subject (nsubj) of the token *kissed*, which allows us to infer that she is the agent doing the kissing. Similarly, the token *John* is the direct object (dobj) of *kissed*, which allows us to infer that *John* is the recipient of the kissing. Directed actions can be identified within text by writing a series of extraction rules that take dependency annotations and part-of-speech tags as input. For instance, a simple rule of the form [Extract all nsubj-VERB-dobj paths] would allow us to extract the information that she kissed him from the example sentence in Figure S1.

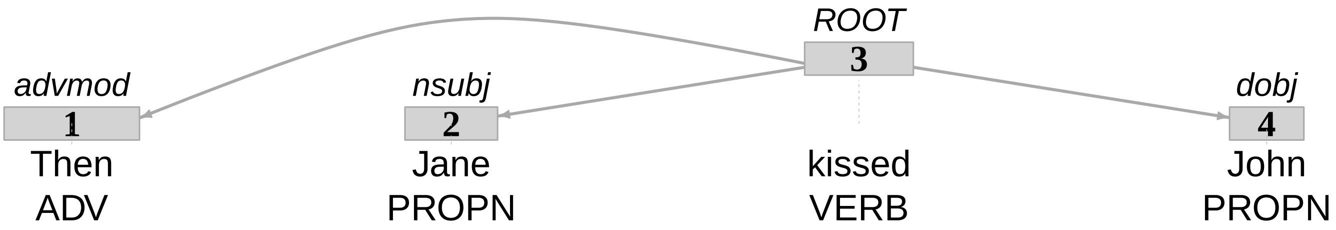


**Fig. S1.** A simple sentence with dependency annotations and part-of-speech tags.

Most naturally occurring English sentences are considerably more complex than this example. To illustrate this, consider the sentence in Figure S2. Here *Jane* is still associated with an action (chased). However, syntactically, the relationship between the two tokens is mediated by *started* as the main verb of the sentence (ROOT) and by the open clausal complement *run* (xcomp). Furthermore, *Jane* is itself not the primary subject associated with these verbs. Rather, it is a conjunct dependent (conj) of the nominal subject *John* (njubj). Nonetheless, semantically, it is obvious that *Jane* is chasing.

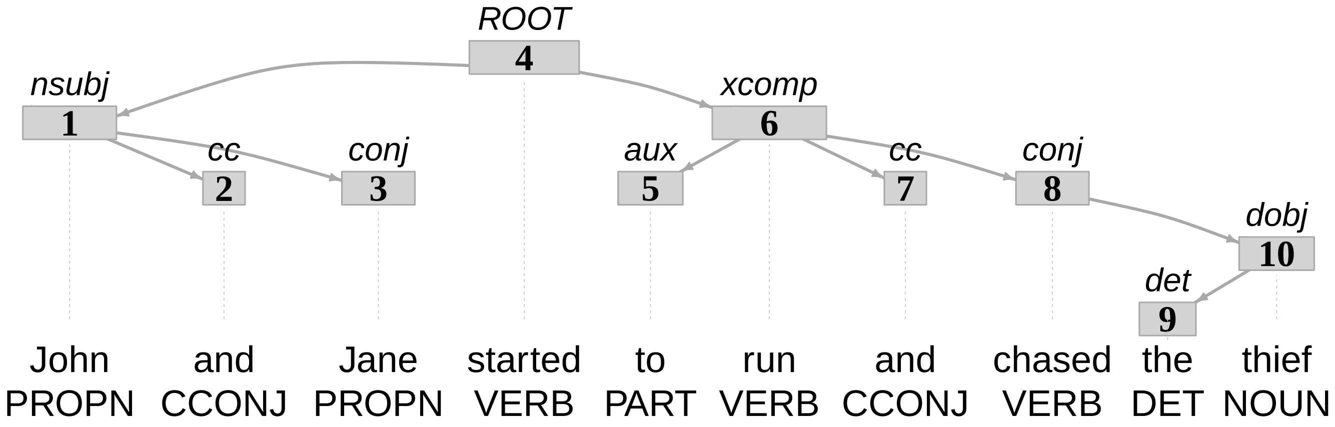


**Fig. S2.** A more complex sentence with dependency annotations and part-of-speech tags.

The sentence is an example of how syntactic relations can have more formal complexity than semantic ones. To account for this complexity, I build on a large number of rules that map syntactic structures into a semantic grammar structured around textual entities. This makes it possible to extract triplets of agent, action, and recipient from textual data. In other words, I extract semantic relationships that are grounded in syntax. The dependency grammar underlying these efforts is the ClearNLP scheme (1) that is used by SpaCy annotation pipeline used for parsing (2). In Table S1, an example sentences to illustrate scope of extraction rules is shown. Note, however, that the table is not exhaustive of all possible patterns, for

the rules account combinations of the different scenarios shown the table – say a combination of verb conjuncts and an open clausal complement, as seen in Figure S2. A comprehensive discussion of considerations behind extraction rules can be found in Stuhler (3). The code for all rules, including annotations with example sentences for each rule can be found on the semgram R package's website (version 0.1.1, 47) (4)*. One difference between the original scope of extraction rules described by Stuhler (4) is that preposition-mediated actions (see rows 10-13 in Table S1) are considered. This change implies that for the analyses in this study composites of verb and preposition (e.g., yell-at, pass-to, steal-from) are considered as actions when associated with an object . This makes it possible to obtain richer information about the relationships between characters.

**Table S1. Exemplary sentences demonstrating scope of extraction rules.**

| # | Sentence | Extract | Comment |
|---|---|---|---|
| 1. | 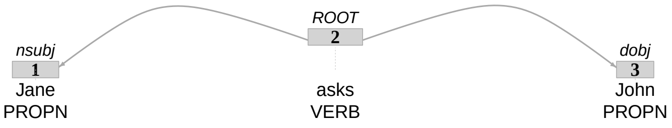  | *Jane—ask—John* | Transitive verbs ("asks") with a nominal subject ("Jane") and a direct object ("John") are the simplest and most frequent syntactic forms leading to extractions. |
| 2. | 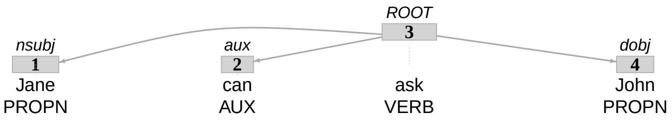  | *Jane—ask—John* | Such transitive verbs are also extracted when the relationship between the verb ("ask") and the nominal subject ("Jane") is mediated by an auxiliary verb ("can"). |
| 3. | 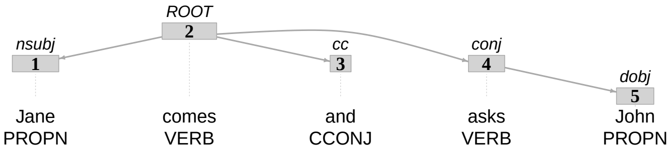  | *Jane—ask—John* | Similarly, transitive verbs are considered when their relationship to the nominal subject is mediated by a conjunct dependent ("comes"). |
| 4. | 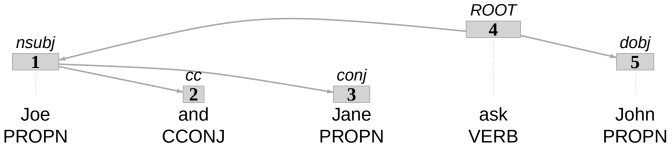  | *Joe—ask—John,* *Jane—ask—John* | Instances in which an entity ("Jane") is a conjunct dependent of a transitive verb's ("ask") nominal subject ("Joe") are also considered for identifying actions. |
| 5. | 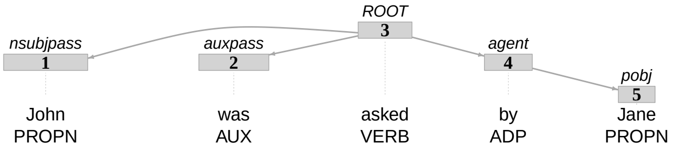  | *Jane—ask—John* | Passive constructions with "by" are considered for identifying actions, but are inversely coded. |
| 6. | 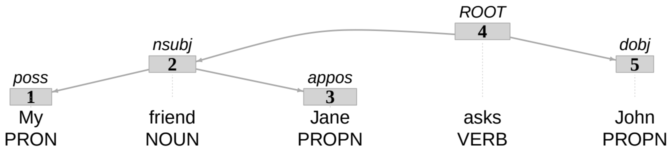  | *Jane—ask—John* | Instances in which an entity ("Jane") serves as appositional modifier of a transitive verb's ("asks") nominal subject ("friend") are also considered. |

Continued on next page

*Which can be found at https://github.com/omstuhler/semgram/tree/master/R

**Table continued from previous page**

| | Sentence | Extract | Comment |
|---|---|---|---|
| 7. | 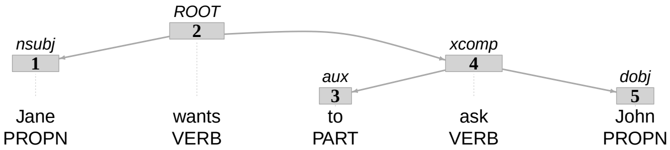 | *Jane—ask—John* | A transitive verb serving as clausal complement (“ask”) of a verb (“want”) is considered an action associated with that verbs nominal subject (“Jane”); unless it has its own nominal subject (“you” in second sentence). |
| | 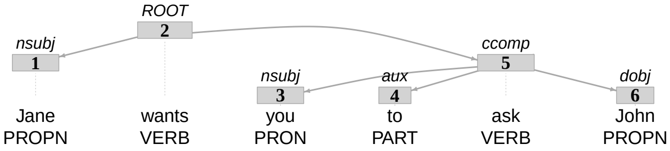 | *You—ask—John* | |
| 8. | 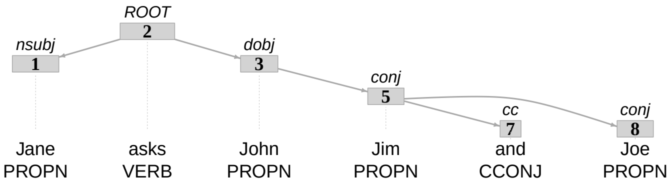 | *Jane—ask—John, Jane—ask—Jim, Jane—ask—Joe* | When there are multiple conjunct objects (“Jim” and “Joe”) of a transitive verb (“ask”), all of them are considered as recipients of the action implied by the transitive verb. |
| 9. | 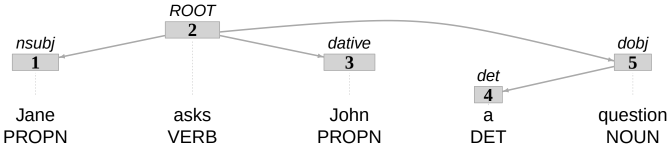 | *Jane—ask—John, Jane—ask—question* | Dative objects (“John”) of ditransitive verbs (“ask”) are also considered recipients. In some rare cases, where ditransitive verb has two distinct characters as direct object (“John”) and dative object (“him”), both are considered recipients. |
| | 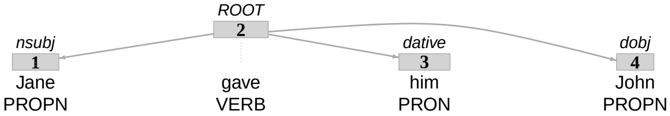 | *Jane—give—he, Jane—give—John* | |
| 10. | 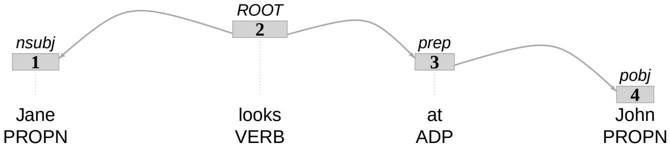 | *Jane—look-at—John* | Quasi transitive verbs (“looks”) that link to an object (“John”) via a preposition (“at”) are considered actions. In such cases, the composite of verb and preposition is considered as action (“look at”). |

Continued on next page

**Table continued from previous page**

| Sentence | Extract | Comment |
|---|---|---|
| 11. 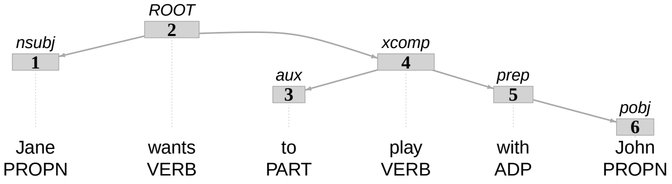  | *Jane—play-with—John* | Like with regular transitive verbs, preposition-mediated verbs ("play with") are considered when they are the clausal complement of an another verb ("wants"). |
| 12. 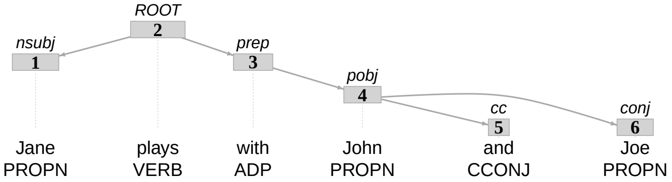  | *Jane—play-with—John, Jane—play-with—Joe* | Also, like with regular transitive verbs, conjunct objects ("Joe") are considered as recipients of the action implied by the quasi transitive ("plays with") verb. |
| 13. 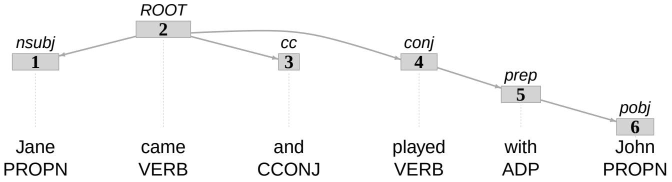  | *Jane—play-with—John* | Similarly, quasi transitive verbs ("played with"), like regular transitive verbs, are considered when their relationship to the nominal subject is mediated by a conjunct dependent ("came"). |

End of table

For validation, 400 actions sent between book characters were sampled and annotated regarding whether the action was correctly extracted. [†] In this annotation, we considered whether the respective verb actually related the two respective entities as subjects and objects. Half of the sample was annotated by two independent coders (Cohen's $\kappa = .75$) and disagreements were resolved deliberatively. Overall, 95.3% of extractions in the sample were correct. Among the 4.8% of incorrect extractions, the most common source of error was that an action was either attributed to the wrong agent entity or the wrong recipient entity. Below are some examples drawn from the sample to illustrate common forms of errors. The dataset with annotations and comments will be made available with the supplementary material.

- ENTITY-114 ENTITY-114 looked at ENTITY-191 with interest, and ENTITY-191, affecting not to notice the survey, watched ENTITY-90 ENTITY-90.
  - ENTITY-114 watch ENTITY-90 was extracted. This is wrong for the subject associated with "watched" is ENTITY-191, not ENTITY-114.
- ENTITY-188 leaped lightly from ENTITY-188 horse, and resigning ENTITY-3184 to ENTITY-123, ran up the steps, and encountered the purple face and blazing eyes of ENTITY-188 angry ENTITY-1373.
  - ENTITY-188 run ENTITY-3184 was extracted. This is wrong for "run" is not a transitive verb in this sentence.
- ENTITY-42 did not care to appear at ENTITY-69's until ENTITY-42 arrangements were more complete.
  - ENTITY-42 appear-at ENTITY-69 was extracted. This is incorrect, for the object should be the genitive "'s" but it makes no sense to say that ENTITY-42 appears at ENTITY-69)

One thing we noticed during annotation is that the composites of verb and preposition would sometimes be semantically

[†] With the Hathi Corpus, I unfortunately only had direct access to the processed and modified outputs of BookNLP while the full texts remain accessible only via a server interface with considerable amounts of lag. Because this form of validation requires reading full sentences of the original text, the US Novel Corpus (USNC) was used for validating action extraction. As discussed in more detail in Appendix Section F, this corpus is smaller but similar to the Hathi in terms of its composition. I also demonstrate below that all main findings reproduce with the USNC.

ambiguous. For instance, in the sentence below, the triplet [Entity-0]-[repose-in]-[ENTITY-91] was extracted from the following sentence:

- ENTITY-0 am going-to repose a trust in ENTITY-91 that ENTITY-0 cannot share at present with any one else – not even ENTITY-0 ENTITY-734.

The direction of the action is correctly identified. However, it is easy to see how, without the direct object *trust*, the extracted action lacks context and is semantically ambiguous. These cases account for 5.3% of the validation sample and were coded as correct. Note, however, that the vast majority of preposition composites (as defined above) is not semantically ambiguous. Ultimately, all preposition mediated verbs were considered for the analyses because even if the composite extraction is semantically ambiguous without context, they nonetheless constitute a subject-object relation. This is sufficient for the purposes of this paper since the primary quantity of interest in the analysis is the number of sent and received actions.

Finally, it is worth noting that the scope, even of this comprehensive rule set, has limits. Broadly speaking, it only accounts for instances in which an entity is either a direct or indirect object of a (preposition-mediated) action or a conjunction-mediated dependent thereof. However, it does not account for phrases that include dual conjunction mediation. For instance, in the sentence in Figure S3., the relationship between *entered* and *me* is mediated not just by the preposition *with*, its object *conversation*, and the secondary preposition *with*. To extract an action that adequately represents the relationship between *she* and *me*, one would have to extract the entire phrase *entered into conversation with*. This is not covered by the rules as these only covers actions representable by a single verb (e.g., *kiss*) or by a combination of preposition and verb (e.g., think-about). Future research may further extend the scope, though whether this is worthwhile will depend on the use case, as complex phrases such as the one in Figure S3 tend to be rare and thus lead to sparsity levels unconducive to many analytic purposes. Further discussion of these and other issues can be found in a recent article by Stuhler (3).

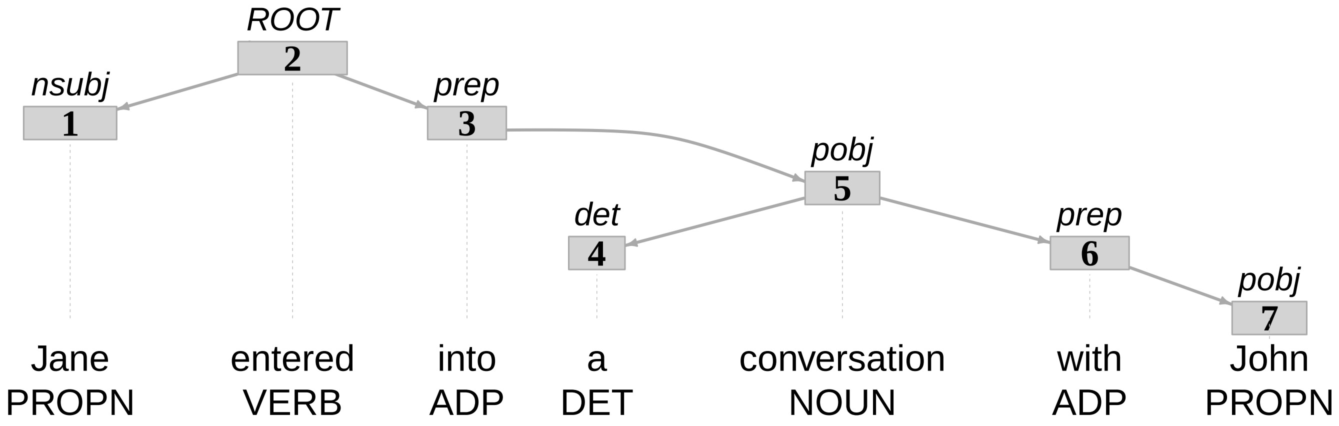


**Fig. S3.** Dual preposition mediated relationship between verb and object.

## B. Regression models

Table S2 documents the linear mixed models used to estimate the male agency surplus within dyads ($Y_{abmw}$), that is, the percentage point surplus in the male action share within a dyad (for variable descriptives and details, see Appendix Section D). Considered are only dyads with at least 5 exchanged actions that relate a male ($m$) and female ($w$) character. Each dyad is contained in a book ($b$) written by a specific author ($a$). The full model (column 7) is the basis for the effects presented in Panel B of Figure 3 in the main manuscript and includes random intercepts for authors ($A_{0a}$), books ($B_{0b}$), male characters ($M_{0m}$), female characters ($W_{0w}$), as well as fixed effects for the logarithmized and z-transformed number of actions within a dyad ($Q_{abmw}$), the male surplus in degree within a dyad ($D_{abmw}$), the male surplus in effective ego network size within a dyad ($E_{abmw}$), the male surplus in the betweenness centrality ($C_{abmw}$), the gender of a book's author ($G_a$), a variable indicating whether the author is a feminist ($F_a$), and the decade the book was written in ($D_b$). It is specified as:

$$\begin{aligned} Y_{abmw} =& \beta_0 + A_{0a} + B_{0b} + M_{0m} + W_{0w} + \\ & \beta_1 \log(Q_{abmw}) + \beta_2 D_{abmw} + \beta_3 E_{abmw} + \beta_4 C_{abmw} + \beta_5 G_a \quad + \beta_6 F_a + \beta_7 D_{ab} + e_{abmw}, \\ & e_{abmw} \sim \mathcal{N}(0, \sigma^2) \end{aligned} \tag{1}$$

Models were fit with the lme4 R package (5) and assume normally distributed intercepts and residuals. Cluster-robust standard errors at the author level were estimated with the clubSandwich R package (6). The reported marginal and conditional pseudo $R^2$ metrics reported in Table S2 are those proposed by Nakagawa et al. (7). Models 6 and 7 have fewer observations because books of authors for whom there is no gender prediction were dropped.

Note that the main paper states that 18.7% of the raw gender agency gap is accounted for by male characters' greater importance; which means that if all dyads had characters with equal degrees, effective ego network size, and betweenness centrality one would, when averaging over the time series, estimate the average male agency surplus to be 18.7% smaller. This should not be confused with the share of the total variance on the dyadic level that is explained by importance imbalances, which is considerably lower. As seen in Table S2, most of the variance in the data is explained by the random effect portion of the model. The models used to estimate the importance-adjusted gap in panel B of Figure 2 of the main manuscript have the simplified form of Model 5:

$$\begin{aligned} Y_{abmw} =& \beta_0 + A_{0a} + B_{0b} + M_{0m} + W_{0w} + \\ & \beta_1 Q_{abmw} + \beta_2 D_{abmw} + \beta_3 E_{abmw} + \beta_4 C_{abmw} + e_{abmw} \end{aligned} \tag{2}$$

They contain no terms for author characteristics or the decade and were fit independently for every window of 10 years. The importance-adjusted gap is then estimated by averaging the predicted male agency surpluses under the assumption of no imbalance in degree, effective ego network size, and betweenness centrality between the male and the female character (i.e., $D_{abmw} = 0$, $E_{abmw} = 0$, and $C_{abmw} = 0$). A notable observation from Table S2 is that the effects for degree and betweenness centrality shrink starkly when including the measure for the effective ego network size and even turn minimally negative. Generally, these measures are highly correlated (see Appendix Section N), and it would seem misleading to say that having high degree or being central (in the sense of betweenness) are not associated with having more agency, as is shown in Models 2 and 3. Yet it appears that net of local network structure (effective ego network size), these two features don't impact agency. To explore this further, I replicate the analyses shown in Panel B of Figure 2 but compute the adjusted gaps under the premise of $D_{abmw} = 0$ (using Model 2), $E_{abmw} = 0$ (using Model 3), and $C_{abmw} = 0$ (using Model 4) individually. I find that individually, these measures would reduce the gap by 15.1% (degree), 15.7% (betweenness), 17.9% (effective ego network size). Together, these results indicate that it is especially a character's relative advantage in bridging structural holes in the character network that comes with agency advantages.

**Table S2. Main regression models for male agency surpluses within dyads**

| | Male agency surplus | | | | | | |
|---|---|---|---|---|---|---|---|
| | (1) | (2) | (3) | (4) | (5) | (6) | (7) |
| Male degree surplus | | 0.096*** | | | −0.021*** | −0.021*** | −0.021*** |
| | | (0.001) | | | (0.003) | (0.003) | (0.003) |
| Male betweenness centrality surplus | | | 0.065*** | | −0.025*** | −0.024*** | −0.024*** |
| | | | (0.001) | | (0.002) | (0.003) | (0.003) |
| Male effective ego size surplus | | | | 0.118*** | 0.177*** | 0.175*** | 0.175*** |
| | | | | (0.001) | (0.005) | (0.005) | (0.005) |
| Logged # of actions (z-transformed) | | 0.735*** | 0.710*** | 0.659*** | 0.642*** | 0.702*** | 0.702*** |
| | | (0.051) | (0.051) | (0.051) | (0.051) | (0.055) | (0.055) |
| Male author | | | | | | 1.721*** | 1.685*** |
| | | | | | | (0.184) | (0.184) |
| Feminist author | | | | | | | −3.899* |
| | | | | | | | (1.892) |
| Constant | 6.624*** | 6.248*** | 6.192*** | 6.008*** | 5.952*** | 9.851*** | 9.886*** |
| | (0.085) | (0.081) | (0.081) | (0.081) | (0.081) | (0.637) | (0.636) |
| Decade fixed effects | No | No | No | No | No | Yes | Yes |
| Pseudo R2 (marginal) | 0 | 0.02 | 0.02 | 0.02 | 0.02 | 0.03 | 0.03 |
| Pseudo R2 (conditional) | 0.29 | 0.28 | 0.28 | 0.28 | 0.28 | 0.28 | 0.28 |
| Number of authors | 40,678 | 40,678 | 40,678 | 40,678 | 40,678 | 30,480 | 30,480 |
| Number of books | 87,531 | 87,531 | 87,531 | 87,531 | 87,531 | 72,546 | 72,546 |
| Number of male characters | 333,330 | 333,330 | 333,330 | 333,330 | 333,330 | 281,758 | 281,758 |
| Number of female characters | 297,249 | 297,249 | 297,249 | 297,249 | 297,249 | 250,713 | 250,713 |
| N | 568,302 | 568,302 | 568,302 | 568,302 | 568,302 | 486,180 | 486,180 |

*$p < .05$; **$p < .01$; ***$p < .001$

## C. Action data and web interface

As a supplement to this paper, a longitudinal dataset with the gender valences of all actions is made available. These data can be downloaded at: https://osf.io/64cwz/.

To facilitate easy exploration of the data, an interactive web interface is provided that allows researchers to trace the gendering and de-gendering of practices. Figure S4 shows the basic layout of the interface. The interface is currently available at: https://gender-agency.shinyapps.io/gender_agency/

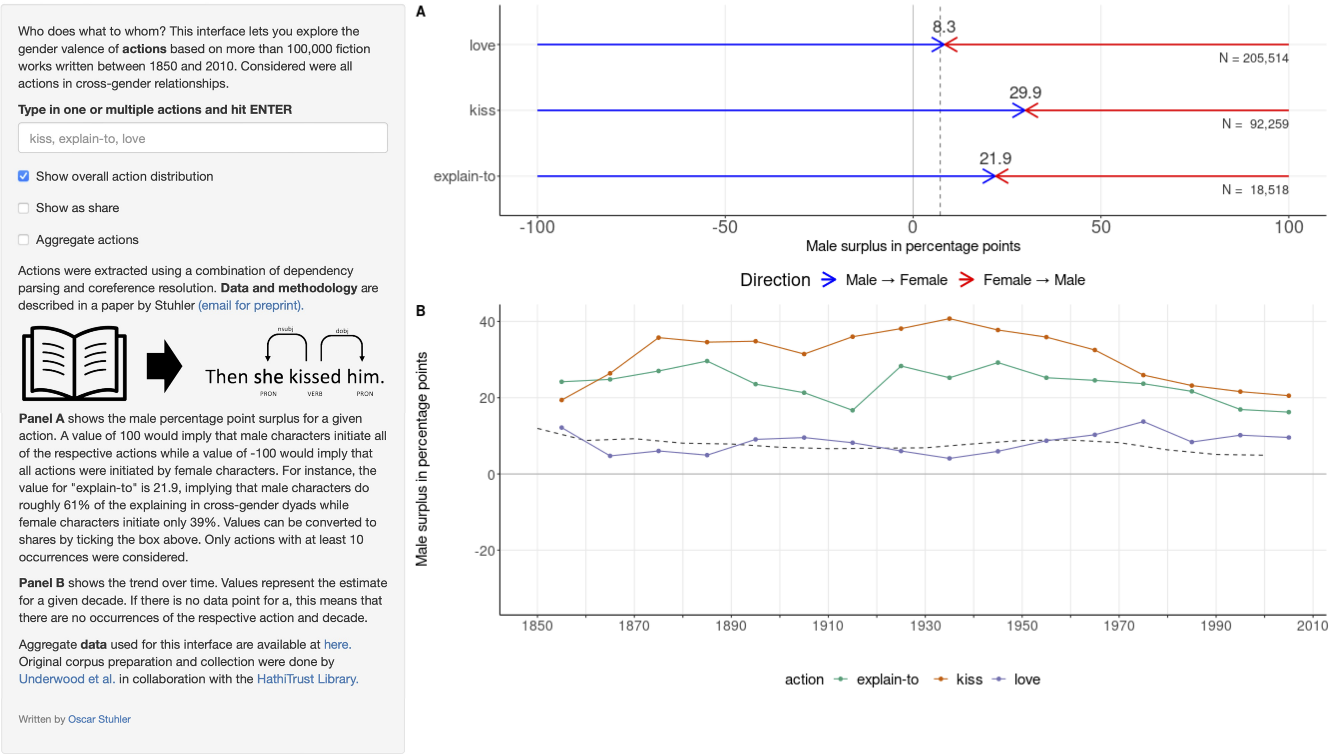


**Fig. S4.** Web interface for exploring agency in cross-gender relationships.

## D. Notes on corpus composition and descriptive statistics

The main analyses are based on the title list of Underwood and colleagues' NovelTM dataset. The title list is an effort to identify the earliest copy of every novel in the larger NovelTM dataset, which often contains multiple editions for a work. A detailed description of this corpus is provided by Underwood and colleagues (8), and additional information can be found in the documentation provided on the associated GitHub repository [‡]. The original title list contains 138,137 works written between 1527 and 2010. However, the data is sparse the further one goes back in time. Therefore, I decided to focus the analyses on works written in or after 1850, which leaves 126,589 works. Data were further constrained in two important ways.

First, note that the NovelTM provides two relevant dates for a title: the latest possible year of composition (the NovelTM's *latestcomp* variable) and the inferred year of publication (the *inferreddate* variable). In the vast majority of cases (88%), these dates coincide. However, there are two scenarios in which they don't. First, sometimes a copyright or first publication date for prior to *inferreddate* is recorded. Second, some works are published after (sometimes long after) an author's recorded date of death. I primarily aim at capturing change in literary production, and given that the publication date is, in some cases, long after the latest possible composition date, I decided to build the analyses on the latter. However, I noticed that relying on the *latestcomp* variable in the NovelTM could lead to major biases for single years, especially in the 19th century. This is because there are numerous posthumously published titles of prominent authors (i.e., authors represented in the corpus with many works) for which the true composition date is unknown, and *latestcomp* was imputed with the death date of the author. For example, roughly half of the titles with a latest composition date of 1870 were authored by Charles Dickens and Alexandre Dumas. Therefore, titles were removed from the corpus, in which the latest year of possible composition is equal to the death date of the author, and the publication date is after the death year of the author, which applies to 8.8% of the corpus. There is simply not a good enough estimate for when these titles were written to make them useful to the analyses.

Second, I decided to remove works the title of which indicates that they are part of a multi-volume collected edition. Given that most collected editions are published posthumously, most of these works had already been eliminated by the prior step, so that only another 0.9% of the data is removed in this step. The rationale for making this an additional step is similar to the one above. I found that the latest date of possible composition for collected editions was usually the title's publication date. However, for collected editions that include many works, this seems not particularly realistic. More importantly, multi-volume collected editions could lead to bias in the time series, for they could lead to a particular year being overly influenced by the works of a single author.

Overall, this leaves 111,723 titles. I create an author ID for each unique author name (as recorded in the *author* variable of the NovelTM). For 6.2 % of the titles, no author was recorded, so that I assigned a unique author ID to each of them. In total, there are 51,981 unique author IDs. However, the true number of distinct authors is likely to be slightly smaller than this, for there are distinct spellings of the same author's name, and some of the titles with no recorded author may have actually been authored by authors present in the corpus. Of these titles, 87,531 (78.3%) contain cross-gender dyads with at least five exchanged actions. Figure A 5 shows the distribution of these works over time. As noted above, the data is relatively sparse at the beginning of the time series, which is why I use decade fixed effects where appropriate.

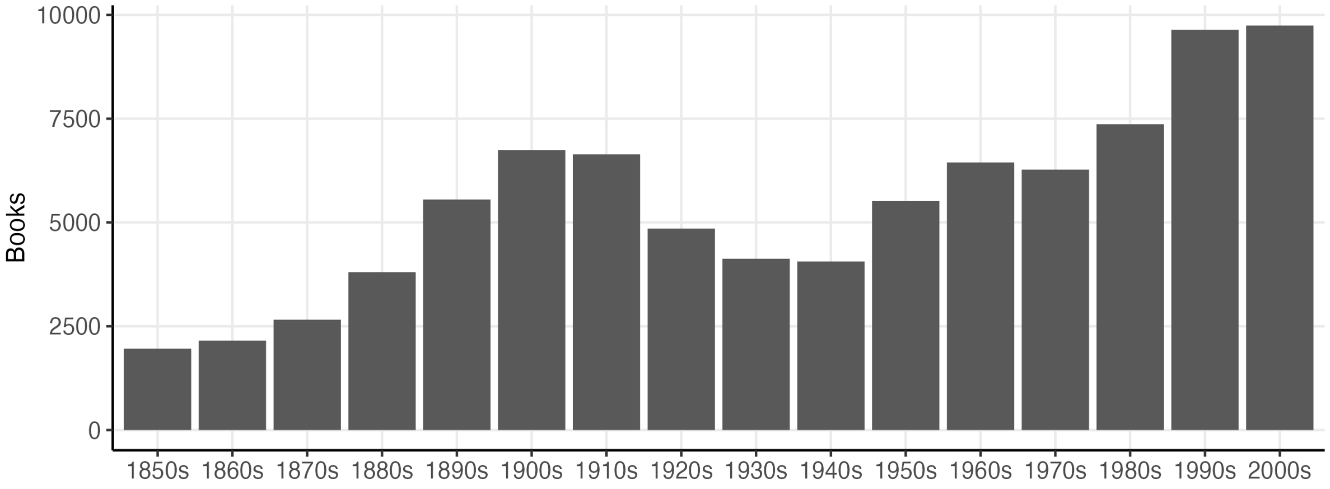


**Fig. S5.** Number of books containing cross-gender dyads over time. Note that 2010 was coded as 2000s.

[‡] Which can be found at https://github.com/tedunderwood/noveltmmeta

Figure S6 shows a histogram of the primary dependent variable over all 568,302 dyads with more than five exchanged actions. The distribution is almost normal throughout all data segments in terms of the number of exchanged actions, as shown in Panel B. The average male agency surplus is 6.4. Note that this is slightly smaller than the pooled average over books reported in the main paper (7.4) because the number of relationships in a book is negatively correlated with the average male agency surplus ($r$ = -.05).

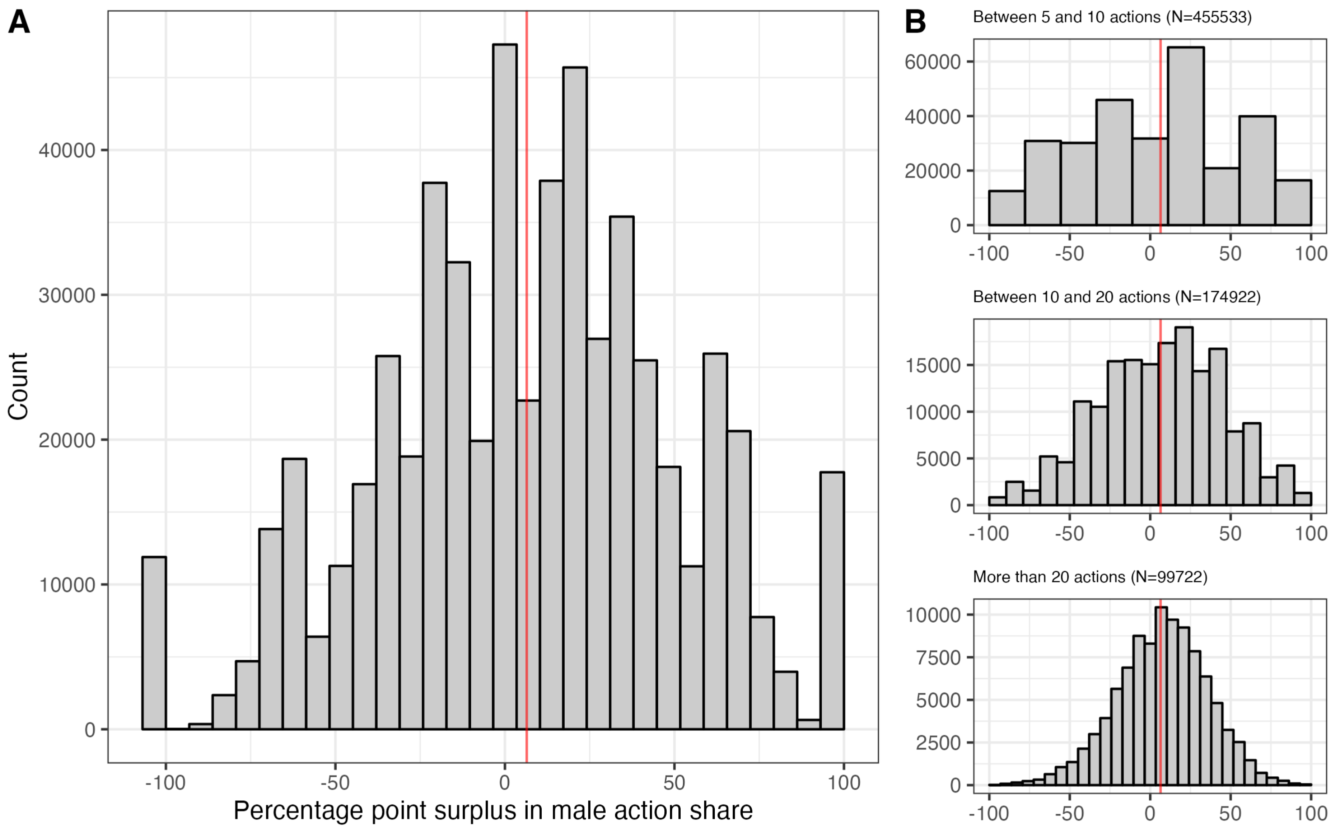


**Fig. S6.** Histogram of the primary dependent variable. Panel A shows the overall distribution of the male agency surplus, with a mean of 6.2. Panel B shows the distribution for different segments of the distribution in terms of the number of actions within a dyad.

The surplus in male agency can also be found in thick and thin relationships, that is, irrespective of the number of actions, as shown in Panel A of Figure S7. Thicker relationships tend to have slightly higher male agency surpluses. The figure also shows that the variance in agency is strongly associated with the thickness of the relationship, with thicker relationships having more equally distributed agency. Panel B shows the distribution of the number of exchanged actions per dyad.

Expanding on the figure in the main manuscript, Figure S8 shows the directional distribution of the 50 most frequent actions in cross-gender dyads, that is, whether they are directed from male characters towards female ones or the other way around. Together, these 50 actions account for 52.9% of the 8.4 million cross-gender actions in the relationships of the corpus. I also show the 25 actions with the strongest imbalance in each direction among actions that occur at least 1,000 times. The web interface discussed in Appendix Section C allows for a similar examination of the full data.

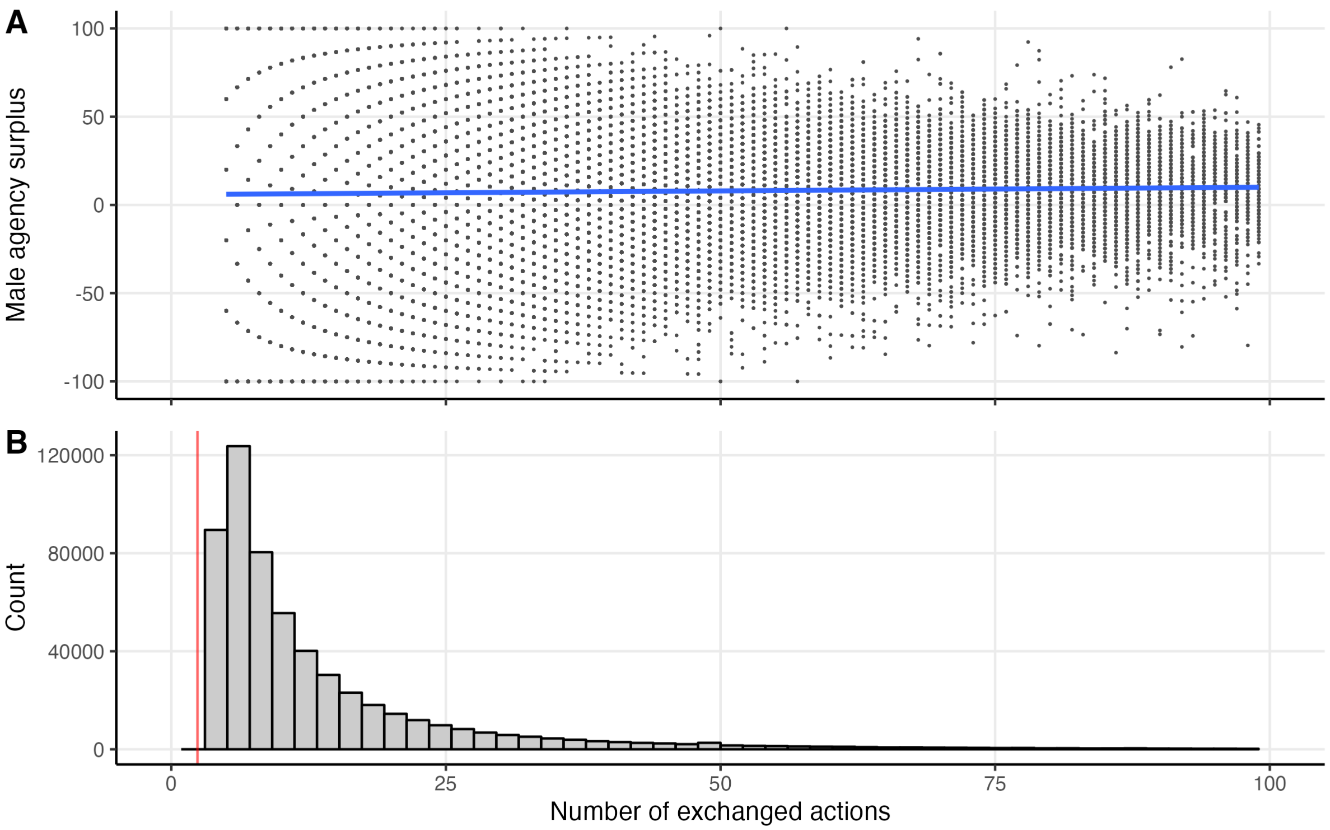


**Fig. S7.** Distributions of key variables and data. Panel A shows the agency distribution for dyads by the number of exchanged actions within the dyad. The male agency surplus is slightly higher in dyads with more actions. Panel B shows the distribution of the number of exchanged actions per dyad. The range of the x-axis was censored at 100, which covers 99.3% of all dyads.

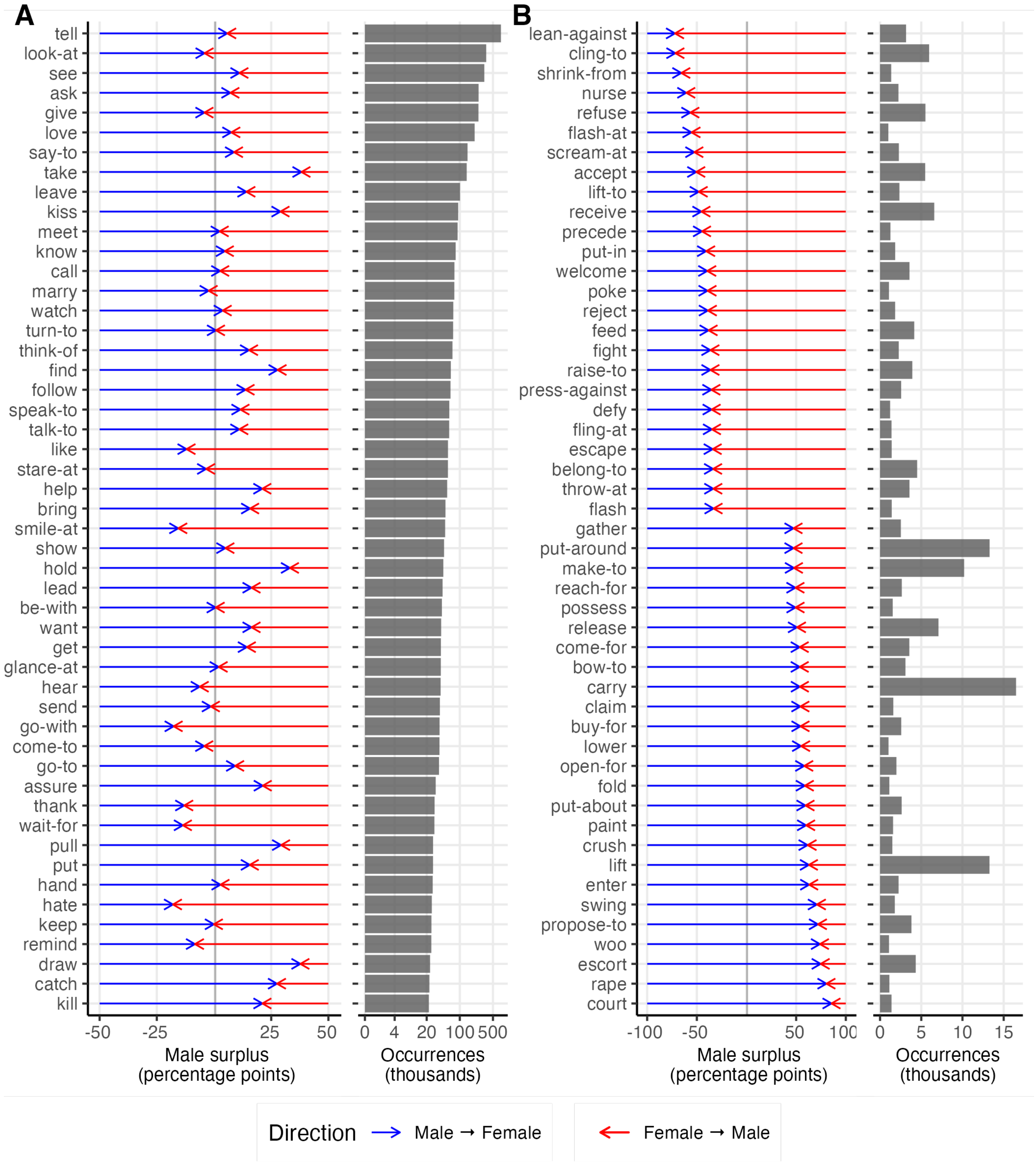


**Fig. S8.** Most frequent actions and actions with highest gender imbalance. Panel A shows the 50 most frequent actions within cross-gender dyads sorted by the number of occurrences. The red and blue lines represent the share of these actions' occurrences in either direction. In the upper most rows, for instance, we see that male characters "tell" female characters slightly more often than vice versa. Meanwhile, female characters "look-at" male characters more often than the other way around. Note that the x-axis is cut off. For Panel B, I selected the 25 actions with the highest imbalance in either direction among actions with at least 1,000 occurrences.

## E. Identifying feminist authors

To identify authors commonly regarded as feminists, I initially accessed crowd-sourced book lists from goodreads.com (e.g., "best feminist fiction" or the "feminist fiction shelf"). Works are voted into these lists by website users. While many of the authors of the final list featured in these lists, the goodreads.com lists turned out to be heavily biased in favor of books with a broad readership. Numerous books with, it seemed, questionable feminist merits, appeared to have been voted into the list simply because they had many readers. Given this, I decided to use a more curated approach and searched for reading recommendation lists and "best of" compilations published by a variety of sources. I found seven distinct lists, published by magazines (e.g., Bustle magazine), archives (e.g., literaryladiesguide.com), as well as educational websites (e.g., thoughtco.com). These lists all offer compilations of feminist writers, often framed as reading suggestions with titles like "12 Classic Feminist Authors to Discover or Rediscover." Some of the lists focus more on contemporary literature, while others feature more works regarded as classics. The lists' foci lie on fiction, though they also contain a few authors who are primarily theorists but occasionally wrote fiction (e.g., Simone de Beauvoir). The sources, together with short descriptions, are listed in Table S3. The links to the lists are provided in the references section, referenceable via the list name. Neither of the sources listed in Table S3 can be regarded as the ultimate source, but together they constitute a good representation of which fiction writers are commonly considered feminist. This is corroborated by the considerable overlap between lists.

**Table S3. Lists of feminist authors.**

| List name | Short description | Authors | Quote |
|---|---|---|---|
| fairygodboss.com (2022) | This website is an online community for women to find jobs and career advice. The list was posted as article in its "Articles & Advice" section. | 27 | "27 Feminist Writers You Need To Read ASAP. All women should make a point of reading the works of these 25 feminist writers. [. . . ] Essentially, feminist literature covers a wide range of written expression, but what they all have in common is a focus on the female experience and how it changes, expands, and evolves." |
| becomeawritertoday.com (2023) | becomeawritertoday.com is a website "dedicated to the craft of writing." It contains advice and job listings for aspiring writers but also articles on particular writers or genres. | 22 | "Top 22 Feminist Authors to Discover for Yourself. Discover the voice of women by reading one of these top 22 feminist authors. [. . . ] Whether you are a feminist yourself or simply want to understand the feminist movement more deeply, this list of twenty-two feminist authors will help you gain the understanding you seek." |
| bustle.com (2016) | bustle.com is an online women's magazine focusing popular culture and politics. It maintains sections on entertainment, style, and wellness, among others. | 10 | "10 Feminist Fiction Every Woman Should Read. [. . . ] So this is a list of women writers that every feminist — newbies and second-wavers alike — should read. It's 100 percent fiction; there's no theory or criticism here, although several of the authors featured here have written non-fiction as well. I've tried to keep this as contemporary as possible, so you can look forward to more from these women in the coming years." |
| theguardian.com (2016) | The guardian is a major UK newspaper. The article used here featured in its books section. While the word "feminist" does not appear in the list's title, this appears to be implied in the article. There is also considerable overlap with other lists, only two of the authors being listed exclusively (Ursula K. Le Guin and Zadie Smith). | 10 | "10 inspiring female writers you need to read. As a response to Gay Talese's failure to name any inspirational female writers, we asked our readers to explain why and how these authors changed their lives. [. . . ] We have celebrated female authors on the Books site before, but we contacted some of our readers and asked them to tell us which female writers shaped their lives. Here are 10 of the most mentioned authors [. . . ]." |

| | | | |
|---|---|---|---|
| literaryladiesguide.com (2018) | This website is an archive “dedicated to classic women authors and their work.” It contained guides, biographies of female writers, and articles, as well as a digital library. | 12 | “12 Classic Feminist Authors to Discover or Rediscover. While this is by no means an exhaustive list of classic feminist authors, it’s easy to argue that these women writers (who are no longer with us) were all visionaries in their unique ways. Fortunately, many more women writing today weave their feminist views into their fiction and nonfiction works.” |
| thoughtco.com (2019) | thoughtco.com is a website with expert created educational content in science and tech, humanities, and languages. The article was featured in their “history & culture” section. | 42 | “42 Must-Read Feminist Female Authors. [. . . ] For the purposes of this list, a feminist writer is one whose works of fiction, autobiography, poetry, or drama highlighted the plight of women or societal inequalities that women struggled against. Although this list highlights female writers, it’s worth noting that gender isn’t a prerequisite for being considered "feminist." Here are some notable female writers whose works have a decidedly feminist viewpoint.” |
| vocal.media (2017) | Vocal.media is a social journalism platform containing stories, guides, and articles from users and professional journalists. | 7 | “Famous Feminist Writers of the 20th Century. Today, we need to pay homage to the most famous feminist writers of the 20th century. Without them, there might be no girl power today. [. . . ] The 20th century saw some of the greatest advancements in the feminist movement — and much of that is thanks to the many famous feminist writers of the 20th century. Their words inspired and also raised awareness of the plight of women in the world.” |

The longest of these lists contains 42 authors, while the shortest contains only 10. Some authors appear on the majority of the lists, such as Virginia Woolf (6), Toni Morrison (4), or Sylvia Plath (4), while others appear only in one, such as Marilyn French. In total, this leads to 69 distinct authors. I then identified all works authored by any of these authors in the corpus. This identification process was done manually to ensure that spelling variations (e.g., the omission of a second given name) would not lead to omissions or errors. In total, 51 of the authors are represented in the NovelTM with fiction works and 44 wrote works between 1850 and 2010 that contain at least one cross-gender relationship that passes the frequency threshold of five actions. Note that this leads to the omission of notable feminist fiction writers, some of which were contained in multiple of the lists (e.g., Roxane Gay).

Together, this leads to the identification of 385 works in the corpus (0.44%), which contain 3,177 dyads (0.56%). Table S4 gives an overview of the authors, sorted by the number of times they were listed. All authors in the final list turn out to be women (George Eliot and George Sand being pseudonyms of female writers), although this was not a necessary criterion for selecting lists. The average gender agency gap among feminist authors is .6 percentage points – suggesting that these authors attribute almost equal agency to male and female characters in cross-gender relationships. When confining the list to authors included in at least two distinct “best of” lists, the average value shrinks to -.8, suggesting a minimal agency surplus for female characters. There are also outliers for which estimates are based on very few actions and dyads. When considering only authors with at least five distinct dyads, the average gap is at -.7.

**Table S4. Feminist authors and male agency surplus.**

| Author | Listings | Books with cross-gender dyads | Cross-gender dyads | Actions in cross-gender dyads | Average male agency surplus |
|---|---|---|---|---|---|
| Woolf, Virginia | 6 | 7 | 68 | 966 | -18.8 |

| | | | | | |
|---|---|---|---|---|---|
| Adichie, Chimamanda Ngozi | 4 | 3 | 35 | 701 | -12.9 |
| Alcott, Louisa May | 4 | 19 | 121 | 1834 | -8.6 |
| Atwood, Margaret | 4 | 11 | 98 | 1982 | 1.7 |
| de Beauvoir, Simone | 4 | 9 | 91 | 2666 | -13.3 |
| Lessing, Doris | 4 | 44 | 396 | 7255 | 1.2 |
| Morrison, Toni | 4 | 8 | 44 | 478 | 18.1 |
| Plath, Sylvia | 4 | 1 | 4 | 51 | -16.4 |
| Carter, Angela | 3 | 11 | 56 | 724 | 16.6 |
| Chopin, Kate | 3 | 3 | 8 | 98 | -10.6 |
| Cisneros, Sandra | 3 | 1 | 1 | 5 | 60 |
| Perkins Gilman, Charlotte | 3 | 2 | 11 | 94 | 4.5 |
| Lorde, Audre | 3 | 1 | 1 | 9 | -55.6 |
| Sand, George | 3 | 10 | 92 | 1354 | 0.8 |
| Tan, Amy | 3 | 5 | 42 | 806 | 2.6 |
| Walker, Alice | 3 | 7 | 45 | 669 | -4.9 |
| Allende, Isabel | 2 | 5 | 47 | 902 | 1.3 |
| Butler, Octavia E | 2 | 4 | 52 | 1245 | 6.8 |
| Eliot, George | 2 | 24 | 229 | 3987 | 15.7 |
| Erdrich, Louise | 2 | 4 | 38 | 544 | -21.7 |
| Hong Kingston, Maxine | 2 | 1 | 1 | 17 | -5.9 |
| Stein, Gertrude | 2 | 2 | 8 | 535 | 21.4 |
| Bronte, Charlotte | 1 | 2 | 20 | 294 | -15.4 |
| Brooks, Gwendolyn | 1 | 1 | 2 | 25 | 25 |
| Buck, Pearl S. | 1 | 46 | 514 | 10303 | 5.9 |
| Cather, Willa | 1 | 13 | 59 | 722 | 20 |
| de Pizan, Christine | 1 | 1 | 4 | 48 | 31.2 |
| Ferrante, Elena | 1 | 2 | 24 | 314 | 4 |
| Franklin, Miles | 1 | 10 | 117 | 1295 | 9.7 |
| French, Marilyn | 1 | 5 | 49 | 1077 | -9.5 |
| Fuller, Margaret | 1 | 4 | 29 | 387 | -5.8 |
| Neale Hurston, Zora | 1 | 7 | 48 | 1454 | 9.3 |
| Orne Jewett, Sarah | 1 | 13 | 40 | 399 | -15.5 |
| Kempe, Margery | 1 | 1 | 18 | 173 | 22.9 |
| Lahiri, Jhumpa | 1 | 1 | 9 | 217 | 6.4 |
| Le Guin, Ursula K | 1 | 16 | 74 | 879 | -8 |
| Lispector, Clarice | 1 | 4 | 45 | 969 | -22.2 |
| St. Vincent Millay, Edna | 1 | 1 | 2 | 61 | 12.2 |
| Nin, Anais | 1 | 15 | 93 | 1440 | -1.5 |
| Oates, Joyce Carol | 1 | 49 | 465 | 9245 | 8.2 |
| Rossetti, Christina | 1 | 2 | 10 | 83 | -1.6 |
| Russ, Joanna | 1 | 5 | 24 | 331 | -16.3 |

214

## F. Replication of main findings with US Novel Corpus

To further assess the robustness of the main findings, I replicate the analyses with a second corpus of fiction works—the US Novel Corpus (9). This corpus (USNC) was assembled by the Chicago Text Lab and contains a total of 9,089 works of English-language fiction that were written by 3,166 authors and published between 1880 and 2000. It contains both highly canonical and mass-market works. Unlike the NovelTM, the USNC puts an explicit emphasis on English-Language Fiction. 87% of its works are authored by American writers, 10.5% by British writers. Whereas the NovelTM marks the attempt to represent the population of fiction works held in U.S. university libraries, also containing translations of works written in other languages, the USNC is more selective and seeks to identify the most prominent works of English-language fiction. Books were selected based on the number of U.S. library holdings recorded by WorldCat, a global catalog of library materials. Because both corpora are based on materials from U.S. libraries, they do not provide a neutral representation of the literary field but emphasize works considered important by a relatively well-educated American audience. More information on the USNC can be found on the associated website (9) as well as in a paper by So et al. (10).

Figure S9 shows the distribution of actions across dyad types for the USNC and the NovelTM, replicating Panel A of Figure 2 in the main paper. The USNC has considerably less data. Both time series use a 10-year rolling average, resulting in a slightly more volatile trend for the USNC, due to it containing considerably less data. Like in the main paper, this figure only considers the main relationships of a book, that is, ones with at least 5 exchanged actions. The distributions are remarkably similar, with *male→male* actions typically making up between 35 and 45% of the actions and *female→female* ones only around 10%. Furthermore, there are more *male→female* actions than *female→male* ones. Put differently, we find a similar gender agency gap in the USNC as in the NovelTM. The trends are also relatively similar, with the share of male→male actions peaking in mid-century and the share of female→female actions starting to increase in the late 1970s. The USNC does not contain works published after 2000, so that we cannot make inferences about the 21st century. A minimal difference between the time trends is that the share of male→male actions in the USNC is slightly lower in the early 20th century than in the NovelTM. However, it seems unwarranted to read too much into this because the USNC does not contain a lot of data for this period.

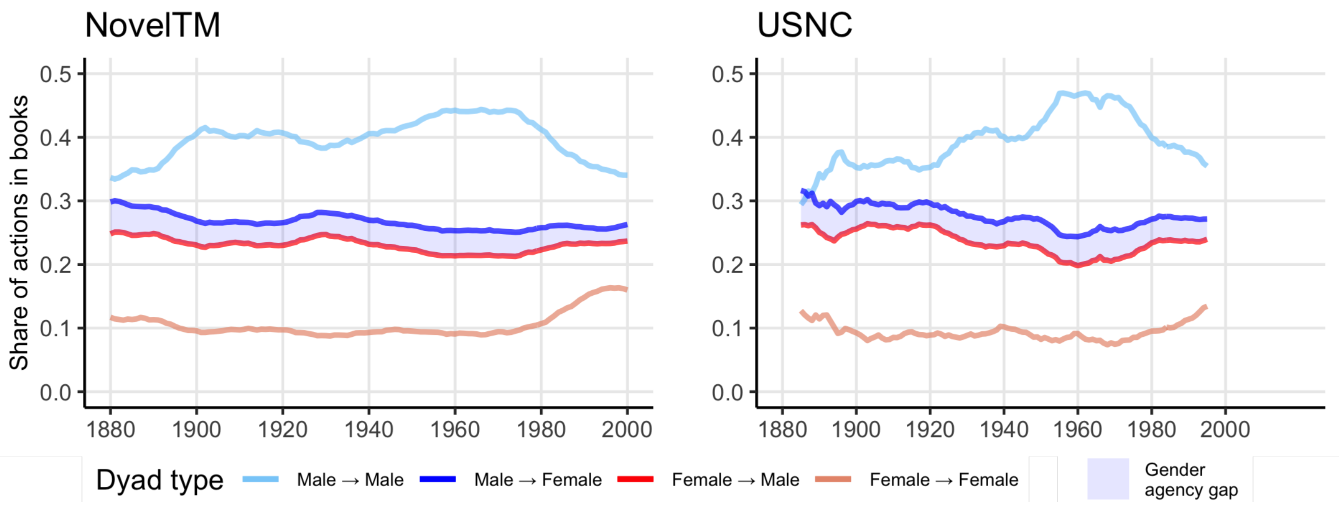


**Fig. S9.** Distribution of actions in NovelTM and USNC. The left panel shows the distribution of actions across dyad types presented in the main paper (Panel A of Figure 2). The right panel replicates the analysis with the USNC. The USNC time series ends in 1990 for the USNC only contains works published up to 2000.

Using the same strategy as with the NovelTM dataset, I identify 119,738 cross-gender dyads between 61,796 female and 65,022 male characters. Like in the NovelTM dataset, there are slightly more male characters. These dyads are nested in 8,725 works. Figure S10 shows the raw and the importance-adjusted gender agency gap in percentage points over time (replicating Panel B of Figure 2 in the main manuscript). Because the USNC contains considerably fewer works, especially for the beginning of the time series, I use a 20-year time window for the USNC estimates, instead of the 10-year time window used for the Novel TM. Overall, the size of the gender agency gap is relatively similar in the USNC. The raw estimate ranges between 6 and 10 percentage points, versus 6 and 9 percentage points for the NovelTM (when considering the same time range). The trends are also similar. While the gap in the USNC starts out lower than in the NovelTM, the uncertainty around these early estimates is considerable. When averaging over the time series, differences in character importance account for 22.0% of the gender agency gap (versus 17.8% in the NovelTM). This implies that in both corpora, the gender agency gap is a feature of cross-gender relationships, rather than a byproduct of books forcusing more heavily on male characters (captures by the three different measures for character importance).

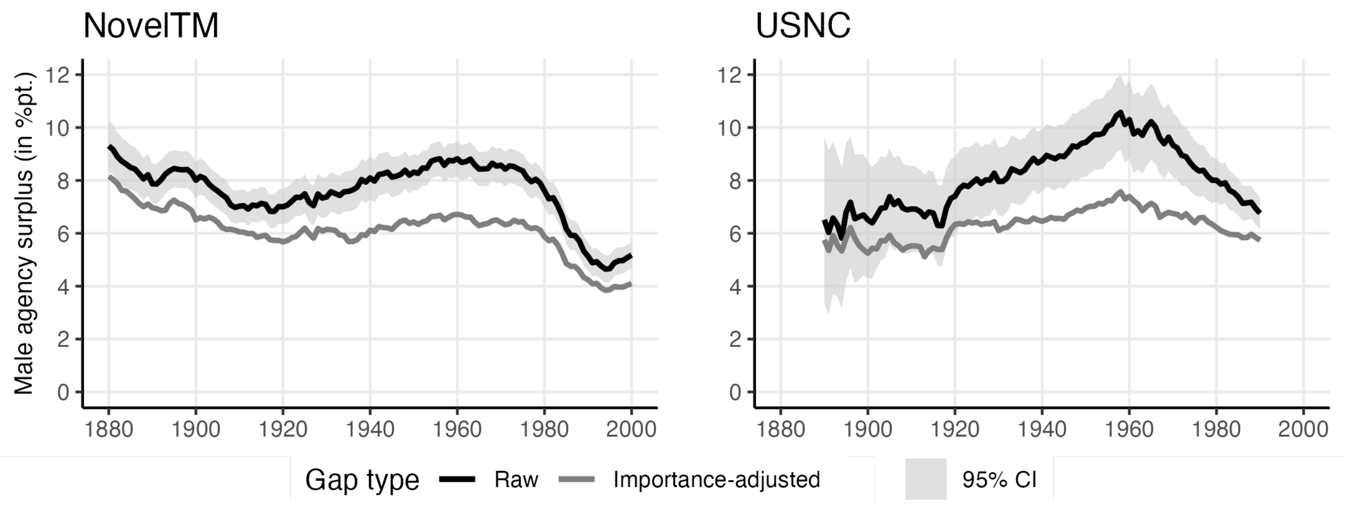


**Fig. S10.** Gender agency gap in NovelTM and USNC. The left panel shows the raw and importance-adjusted gender agency gap presented in the main paper (Panel B of Figure 2). The right panel replicates the analysis with the USNC. For the importance-adjusted gap estimate, just like in the main paper, I fit linear mixed models with random intercepts for male and female characters nested in books and authors and fixed effects for male advantages in degree, betweenness centrality, and the effective ego network size. I then use the models to predict the gap for a scenario in which male and female characters were equally important to each work (i.e., have the same degree, betweenness centrality, and effective ego network size). Because the USNC has considerably less data, I use a 20-year rolling average instead of the 10-year rolling average used for the NovelTM. For the importance-adjusted gap, this means that an independent model was fit for every 20-year window. The USNC time series ends in 1990 for the USNC only contains works published up to 2000.

Unlike the NovelTM, the USNC contains hand-coded gender information. Like the NovelTM, the USNC reflects the underrepresentation of female authors in the literary field, with 62.7% of its works authored by men. Figure S11 shows that in both male and female authors in the USNC attribute more agency to male characters in cross-gender relationships. In support of the finding in the main paper, male authors do this at a higher rate than female ones.

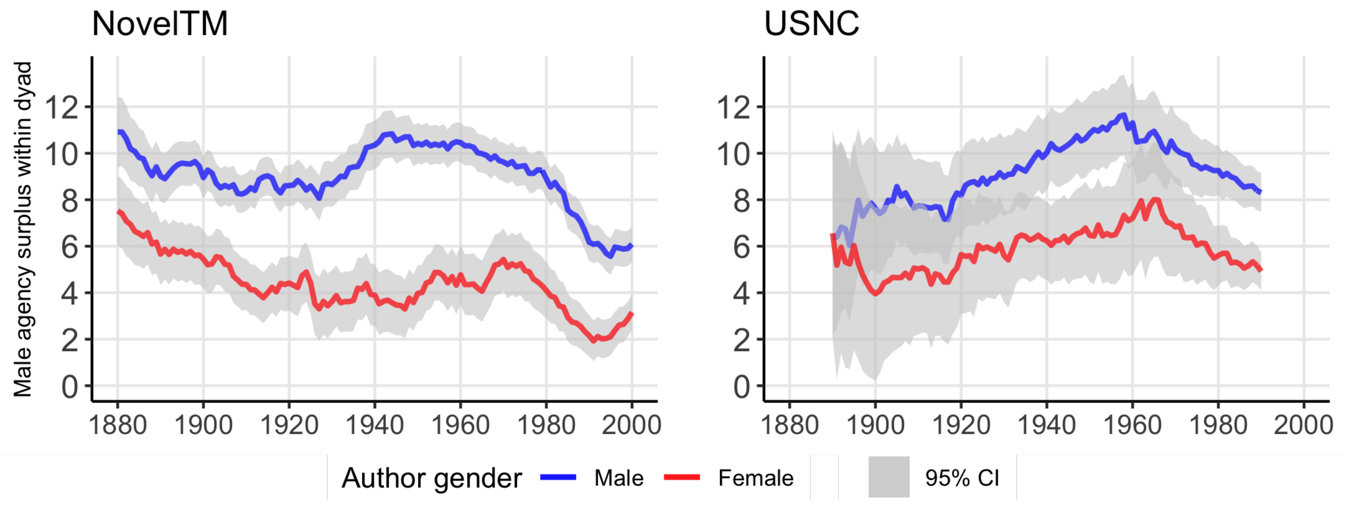


**Fig. S11.** Gender agency gap by author gender in NovelTM and USNC. The left panel shows the raw gender agency gap for male and female authors presented in the main paper (Panel A of Figure 3). The right panel replicates the analysis with the USNC. Bars denote 95% confidence intervals. I use 10- and 20-year moving averages for the NovelTM and the USNC, respectively.

## G. Genre analyses

Unlike the NovelTM dataset, the USNC contains genre tags for its works. Genre labels are binary variables so that each book in the corpus is annotated with none, one, or multiple genre tags. Genre annotations result from a process combining library tags and machine learning, described in more detail by Pruett (11). First, multiple libraries were accessed, allowing for the assemblage of genre annotations for roughly 53% of the corpus. These human labels were then unified and used to predict binary genre tags for the rest of the corpus.

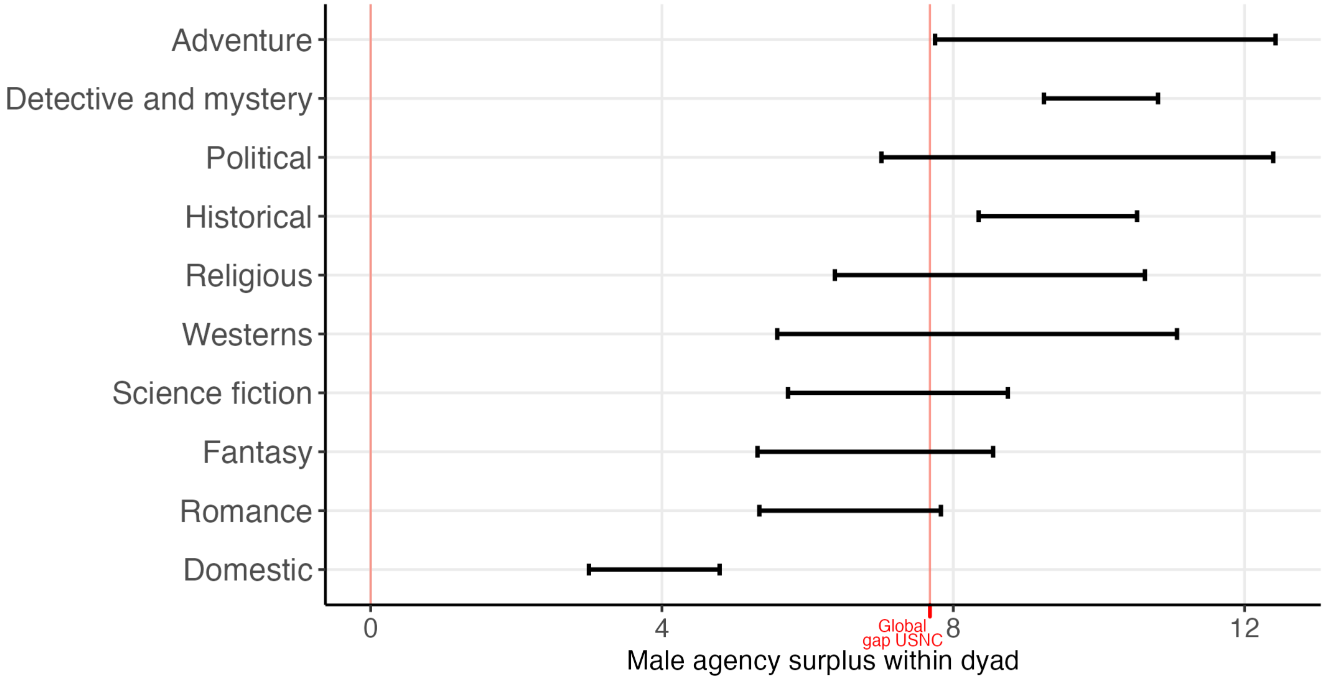


**Fig. S12.** Male agency surplus by genre. Values were averaged over books. Bands denote 95% confidence intervals.

Figure S12 shows the average male agency surplus for each genre. There are particularly high gender agency gaps in the genres *adventure*, *detective and mystery stories*, *political*, and *historical fiction*. On the other hand, *romance* and, especially, *domestic fiction* have a lower male agency surplus. Especially the latter is unsurprising but may be seen as an additional validation of the measure, given that domestic fiction novels focus on heroine characters and female subjectivity (12)—though reading this as emancipatory development is contestable (13, 14). That said, male characters receive more agency in cross-gender relationships in *all* genres.

## H. Identifying author gender

The NovelTM title list by Underwood et al. (8) does not include information on author gender. However, one can make use of the recorded author's name. Gender predictions are based on two aspects: honorifics and given names' gender valence. Given that honorifics are likely to be more reliable indicators of authorial gender, I first used regular expressions to identify authors names that include male (e.g., "Mr.", "Sir", "Sr.") or female honorifics (e.g., "Mrs.", "Miss", "Lady"). Only a relatively small number of author names include such titles, however (2.5%). For the rest, I make use of the given names.

The gender valence of given names varies across time. For instance, Madison was a primarily male name in the first half of the 20th century but changed its gender valence in the 1980s and is now primarily female. To infer an author's gender, data from three sources are used: the US Social Security Administration Baby Names dataset (SSA), the Integrated Public Use Microdata Series (IPUMS), and North Atlantic Population Project (NAPP). The three datasets span different time ranges (1880-2012 for SSA; 1789-1930 for IPUMS; 1758-1910 for NAPP). Birth name data from these sources were integrated in the "gender" R package by Blevins and Mullen (15).

For each work in the corpus, I define a time range to compute the gender valence of a given name. For works where we know the birth year of the author, this time range is 15 years before and after that birth year. While we could constrain ourselves to data from the birth year only, increasing the window allows for better inferences, especially for rarer names. For works with unknown author birth year, I consider the time range between 70 and 20 years before the works composition year. In this way, for instance, the gender inference for a work from 1980 with unknown author birth year will be based on births between 1910 and 1960. Were the author named "Ashley," this would lead us to predict them being male, for the SSA records 86.6% of recorded births in that time range to have been associated with male gender. I use the three datasets (SSA, IPUMS, and NAPP) iteratively in the cases of missing records. For instance, for an author birth date in 1915 (range 1900 to 1930), I first attempt to make a prediction based on the SSA data. If no entry for the given name could be found, IPUMS is consulted, then NAPP. I use the orders SSA-IPUMS-NAPP and IPUMS-NAPP-SSA for the data ranges centering after and before 1900, respectively.

Many authors have multiple given names. The inference process is run on the given names sequentially, so that, should the first given name not allow for a gender inference, the script moves on to the next. Not all works in the dataset have an entry for the name of the author (93.8%), and not all have a first name for which a gender could be inferred. Using honorifics and first names, it was possible to make a gender prediction for 82% of the data in the corpus.

This strategy is imperfect, and there are various potential sources for error. For one thing, some writers use pseudonyms with given names associated with the other gender. Perhaps most famously, writer Mary Ann Evans is known under her pen name George Eliot. Second, given names do not allow for robust gender inference. For instance, the given name of female writer Miles Franklin scales male. Finally, it is worth pointing out that the databanks used stem from birth data in the US (SSA, IPUMS) and northern European countries (NAPP). This implies that we are less likely to correctly predict the gender of authors born outside these countries. In order to assess the quality of the gender estimation strategy, I use the manually-checked title sample of the NovelTM dataset. This is a random sample of the title list, stratified across time, for which Underwood and colleagues (8) manually added columns for authorial gender. Considering the manual annotations as ground truth, overall accuracy of the gender predictions is .97 (N = 1489). Table S5 shows the precision and recall for male and female authors, respectively. These numbers suggest that, overall, honorifics- and name-based gender inference works well in the corpus. The main implication of the error is that the estimates of differences by author gender are conservative and that real differences might be minimally larger than shown.

**Table S5. Accuracy of gender prediction.**

| Precision (male authors) | Recall (male authors) | Precision female authors | Recall female authors |
|---|---|---|---|
| .97 | .99 | .97 | .94 |

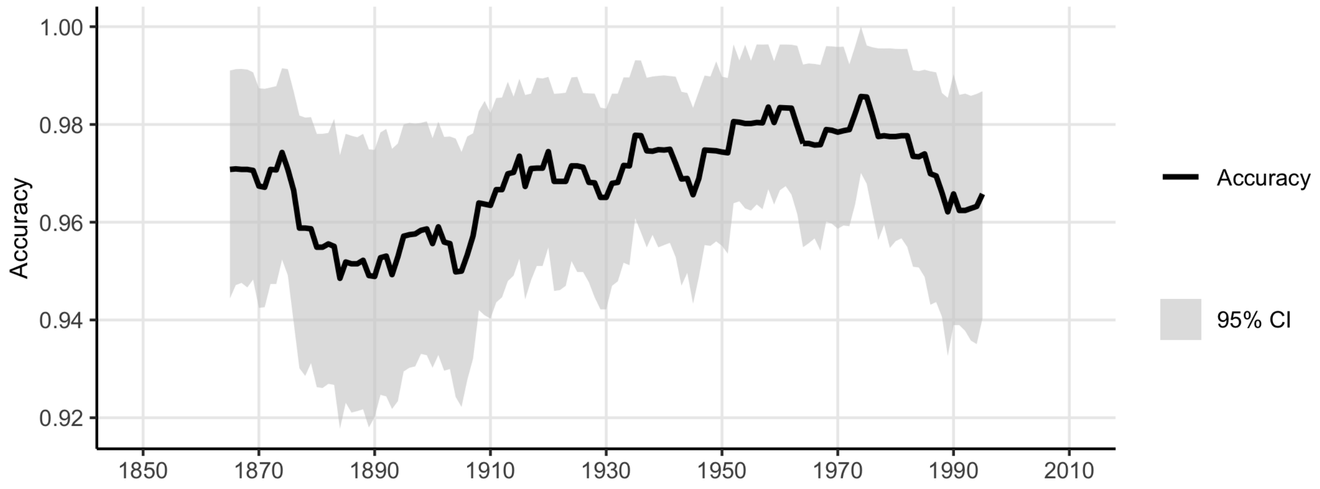


**Fig. S13.** Accuracy of author gender prediction over time. The figure shows the accuracy of the gender prediction approach. Ground truth is provided by the NovelTM's manually-checked title subset, which is a time-stratified random sample of the significantly larger title list we use for the analyses. To measure accuracy across time, we use 30-year moving windows with an average of 291 titles in each window. The grey area indicates bootstrapped 95% confidence intervals.

Figure S13 shows the accuracy of the gender prediction over time, using 30-year windows. Despite some variation, the time series looks remarkably stable, with a range of only .037. Note that the average window here contains only 291 titles, so that, based on the overall accuracy of .97, one would only expect 8.7 falsely predicted cases in a given window. This implies that one should expect some variation simply by chance. Overall, these results suggest that author gender prediction performance does not cause any major biases in the time trends shown in Figure 3 of the main paper.

Finally, I undertook a few steps to further improve the quality of the gender variable. First, there were a couple of authors (15) for whom the process led to diverging gender predictions. This can result from their works having no recorded birth date and being published in different years, leading to different time ranges for gender estimation. I researched and fixed these authors' genders manually. Second, there were a few works (349) for which the above approach led to no gender prediction, but there was a gender predictions for the author based on other works. In those cases, I extrapolated from the evailable gender predictions (four of these works happen to be in the manually-labeled list, and the predictions for them were correct). Third, through the engagement with the data, I occasionally noticed errors in the predicted gender of certain authors and fixed these manually. As was the case, for instance, with female writer Miles Franklin, who is part of the feminist list (see Appendix Section E). I did this for not doing so seemed like a missed chance to improve the quality of the data, if marginally. Fourth, I used the manually coded gender for works contained in the NovelTM's manually-checked title sample, though this only makes for 1.5% of the data. All these steps were done after the validation described above so that the numbers provided should provide, if anything, a conservative estimate of the quality of the gender inference. Using all described measures, I was able to make gender predictions for 92,278 (82.6%) of the 111,723 titles in the corpus.

## I. Spheres of action

To measure agency in the different spheres of action (communication, physical action, emotion and sentiment, perception/cognition, and villainy), five verb dictionaries are defined through a two-step approach. First, the 200 most frequent verbs were examined (considering only those that occur in the main relationships; 1000 for villainy because few of the most frequent verbs were attributable to this dictionary), which, cumulatively, make up 79.6% of all verb occurrences. Where appropriate, verbs were assigned to one of the five dictionaries. In a second step, the initial dictionaries were expanded by prompting GPT-4 to provide potential additions, which were then manually validated. Table S6 shows the five dictionaries used in the analyses.

**Table S6. Dictionaries for spheres of action.**

| Dictionary | Initial verbs (based on frequency) | Generated verbs (based on GPT) | Total occurrences |
|---|---|---|---|
| Communication | tell, ask, say-to, call, speak-to, talk-to, assure, thank, write-to, beg, answer, promise, explain-to, say, warn, invite, write, call-to, inform, persuade, greet, interrupt, address, whisper-to, talk-with, urge, advise, tell-about, bid, speak-of, accuse, read-to, talk-about, encourage, order, mention-to | notify, brief, instruct, command, communicate-to, describe-to, direct, converse-with, query, respond-to, suggest-to, confide-in, declare-to, announce-to, report-to, disclose-to, shout-to, recount-to, express-to, narrate-to, disclose-to, signal-to, gesture-to, pronounce-to, voice-to, hint-to, confess-to, proclaim-to, chant-to, sing-to, remark-to, testify-to, plead-with, beseech, consult-with, update, comment-to, note-to, recite-to | 1,760,481 |
| Physical action | kiss, follow, lead, hold, smile-at, draw, hand, pull, kill, touch, push, sit-beside, carry, accompany, lift, hand-to, hold-to, put-around, stand-before, walk-with, laugh-at, pass, press, hug, sit-with, shake, embrace, stand-beside, turn-toward, hit, run-to, release, grab, dance-with, cling-to | throw, kick, drag, toss, fetch, pour-on, wave-at, point-to, lean-against, brush-against, play-with, bite, swing-at, leap-over, reach-for, shove, move-toward, poke, roll-toward, pat, gesture-to, prod, chase, beckon-to, scratch, slap, strap-to, throw-at, brush, climb-over, crawl-under, rip, tug, twist, sweep-toward, pinch | 756,133 |
| Emotion and sentiment | love, like, hate, forgive, trust, miss, care-for, admire, feel-for, pity | despise, cherish, envy, loathe, adore, detest, fear, resent, yearn-for, disdain, crave, dread, abhor, savor, idolize, mourn, grieve-for, delight-in, long-for, pine-for, lust-after, value, treasure, sympathize-with, ache-for, thrill-to, venerate, smitten-with, bond-with, covet, fume-at, rage-against | 408,032 |
| Perception and cognition | look-at, see, know, think-of, watch, stare-at, hear, glance-at, understand, believe, listen-to, remember, forget, gaze-at, regard, think-about, recognize, look, hear-from, think, look-for, notice, expect, know-about, study, look-after, imagine, glare-at, look-to, feel | perceive, observe, discern, sense, ponder, reflect-on, mull-over, consider, inspect, view, listen-for, glimpse-at, fathom, grasp, appraise, evaluate, judge, comprehend, ponder-on, ruminate-on, wonder-about, dwell-on, interpret, note, deduce, muse-on, inspect, peruse, survey, behold, eye, detect, distinguish, witness, assess, marvel-at, dream-of, fantasize-about, hypothesize-about | 1,347,823 |

| | | | |
|---|---|---|---|
| Villainy | kill, hurt, deceive, hit, lie-to, shoot, frighten, betray, kick, insult, punish, threaten, scare, murder, attack, rob, neglect, ruin, wound, torment, torture, abuse, destroy, harm, trick, punch, strangle, humiliate, rape, injure, cheat, stab, bully, whip, steal-from | belittle, berate, blackmail, bludgeon, browbeat, chastise, choke, con, curse, denigrate, deride, desecrate, drown, enslave, exploit, extort-from, gaslight, harass, manipulate, malign, molest, oppress, perjure, poison, sabotage, slander, terrorize, violate, coerce, defame, degrade, delude, demean, entrap, eradicate, hang | 118,372 |

A central difficulty in making these assignments was that a decent share of the English language's most common verbs can carry a multitude of senses, depending on their context. For instance, as stated in the main manuscript, give could be used to denote a physical action ("give him the apple") but also metaphorically in various ways ("give him an answer", "give him love", "give him attention"). It is therefore unwarranted to assign give to any of the five dictionaries. Generally, a verb was assigned to one of the five dictionaries only if it was highly unequivocally attributable. At first, I considered building on external resources to make such calls, such as the OntoNotes word sense groupings (16). However, I then realized that for the analysis in this paper, it didn't so much matter whether a word could be used in different ways but rather whether it was actually used in different ways in the corpus in the respective instances that involve two main characters.

Consider, for instance, the verb "love," the fifth most frequent verb in cross-gender relationships, with 205,518 occurrences. OntoNotes lists three distinct word senses for "love:" first, to have great affection, liking, or desire for (e.g., "I love you."); second, to thrive on, tend to, or have the need (e.g., "The cactus loves hot air."); third, to have sexual intercourse with (e.g., "We loved all night the day before he left."). A helpful constraint in the assignment process was that we know that the verb was used transitively between two main characters of a book—that is, characters that recur at least so often that they exchanged five actions with another character. This eliminates the second word sense, which, according to the OntoNotes entry, requires a non-human in the recipient position, leaving the first, and third sense. Technically, this means that love is not unequivocally attributable to one of the four dictionaries, for "loving" could describe both emotions or physical activity. However, looking at a sample of sentences in which "love" is used transitively between two main characters, one finds that among these two senses, love is overwhelmingly used in the first and only very rarely to describe sexual intercourse. In the context of the analysis, it is therefore warranted to assign love to the dictionary of emotions and feelings.

To make this process more formal, two independent coders (one of whom was the author) followed these steps:

1. Consider whether the verb is uniquely classifiable as "physical action," "perception and cognition," "communication," or "emotion and sentiment." For many verbs, this is the case. For instance, "think-of" is clearly assignable to perception and cognition.

2. If there were multiple possible senses, look at a sample of ten sentences in which the verb is used transitively between two main characters. If in at least nine of them, the verb is used in one of the five senses, assign the verb to the respective dictionary. Otherwise, leave the verb unassigned. For instance, all ten sentences in the "love" sample use it to describe an emotion and not a physical action. Therefore, "love" is assigned to the "emotion and sentiment" dictionary.

A verb could only be assigned to one of the first four dictionaries; however, overlap between villainy and the other dimensions was permitted. We used the following guiding questions for the five dimensions:

- Communication: Does the verb imply that the subject communicates with the object?

- Emotions and sentiment: Does the verb imply something about the emotions, feelings, or the sentiment the subject holds towards the object.

- Perception and cognition: Does the verb imply something about the perception and cognition of the subject in relation to the object.

- Physical action: Does the verb imply a physical action by the subject towards the object?

- Villainy: Does the verb imply that the subject does something bad to the object.

Agreement between the coders was generally high, with Cohen's $\kappa$ values of .86 (communication), .81 (perception and cognition),.85 (emotion and sentiment), .7 (physical action), and .77 (villainy). Disagreements on specific cases were resolved by deliberation.

After the initial coding of the first, 200 verbs (1000 for villainy), especially the dictionaries for villainy as well as emotion and sentiment were still relatively short. However, going through the most frequent verbs was not a very efficient way to further expand them. Instead, GPT-4 was prompted to come up with potential additions to the dictionaries, 100 for villainy and emotions, 50 for the other four dimensions. Below is an example prompt used to generate additions for the villainy dimension:

> *Below is a list of transitive verbs. All these verbs imply that that the subject does something bad to the object. Please provide a list with 100 more verbs that share this quality.*
>
> *You may use a combination of verb and preposition, such as "lie-to" or "steal-from." Make sure that there are no duplicates in your list and that there are no overlaps between your list and the list below.*
>
> *[kill, hurt, deceive, hit, lie-to, shoot, frighten, betray, kick, insult, punish, threaten, scare, murder, attack, rob, neglect, ruin, wound, torment, torture, abuse, destroy, harm, trick, punch, strangle, humiliate, rape, injure, cheat, stab, bully, whip, steal-from] "*

Two independent coders then again manually assessed these potential additions following the same steps as above. Cohen's $\kappa$ values were somewhat lower for this round with values of .46 (communication), .42 (perception and cognition), .71 (emotion and sentiment), .41 (physical action), .58 (villainy). This is because the GPT suggestions contained a high number of edge cases: the first suggestions were in almost all instances valid additions to the dictionaries but as we went further down the list, this rate would steadily decrease. Together, the two steps ensure that there are at least no obvious omissions of frequent verbs in the dictionaries. If not completely exhaustive, the dictionaries provide a comprehensive and intersubjectively valid device for measuring action in the five different spheres.

Communication (20.8%) and perception and cognition (16.0%) account for most of the exchanged actions in cross-gender relationships, followed by physical action (9.0%), emotion (4.8%), and villainy (1.4%). To contextualize the trends in different spheres of action shown in the main paper, the bottom panel of Figure S14 shows the importance of these forms of action in cross-gender relationships over time. This is done by depciting the share of actions belonging to a sphere for male and female characters. Physical actions have become twice as prevalent over the period studied, while the opposite applies to emotions and sentiment. Communication and villainy have also become notably more prevalent in both male and female actions. Perception and cognition gained importance for cross-gender relationships until the middle of the 20th century before declining again. By and large, trends for male and female characters align. However, there are some noteworthy exceptions. While perception and cognition were equally prevalent in the conduct of both genders until the 1970s, they have remained more prevalent for female characters.

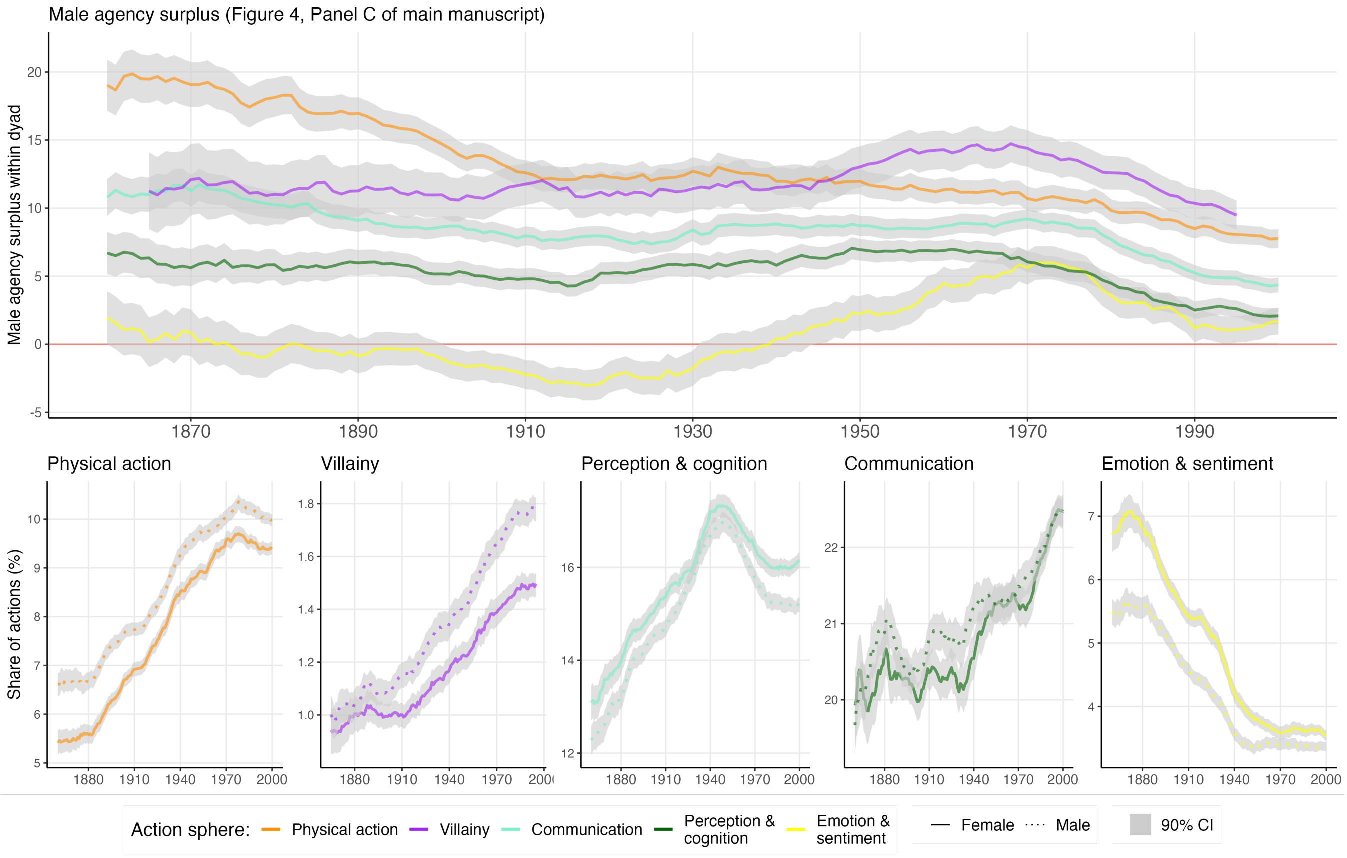


**Fig. S14.** Agency gaps by sphere and shares of action by character gender. The top panel shows the male agency surplus for five different spheres of action (replicating Panel C of Figure 4 in the main paper). The bottom panel shows the share of actions within cross-gender dyads that belong to a particular sphere for male (dotted lines) and female characters (solid lines). Values were averaged over books. For instance, the leftmost panel suggests that when selecting a random cross-gender dyad in a randomly drawn book between 1990 and 2010, one would expect around 6.5% of the actions sent by the male character to be physical actions. Like in the main manuscript, 20-year rolling averages (30 years for villainy) and 90-percent confidence intervals are used.

## J. Analyses without first-person narrator

First-person narrators generally tend to have agency advantages in their relationships. In this section, all main findings are replicated while omitting relationships that contain a first-person narrator (either as a male or female character). BookNLP usually detects first-person narrators and assigns a particular code (code 0) to them. Excluding such characters leaves 444,208 of 568,302 dyads (78%). Figure S18 shows the distribution of actions in the main paper and without first-person narrators. The share of male→male actions is considerably smaller, as 79% of the first-person narrators were classified as male characters. However, the male agency surplus prevails throughout the entire time series.

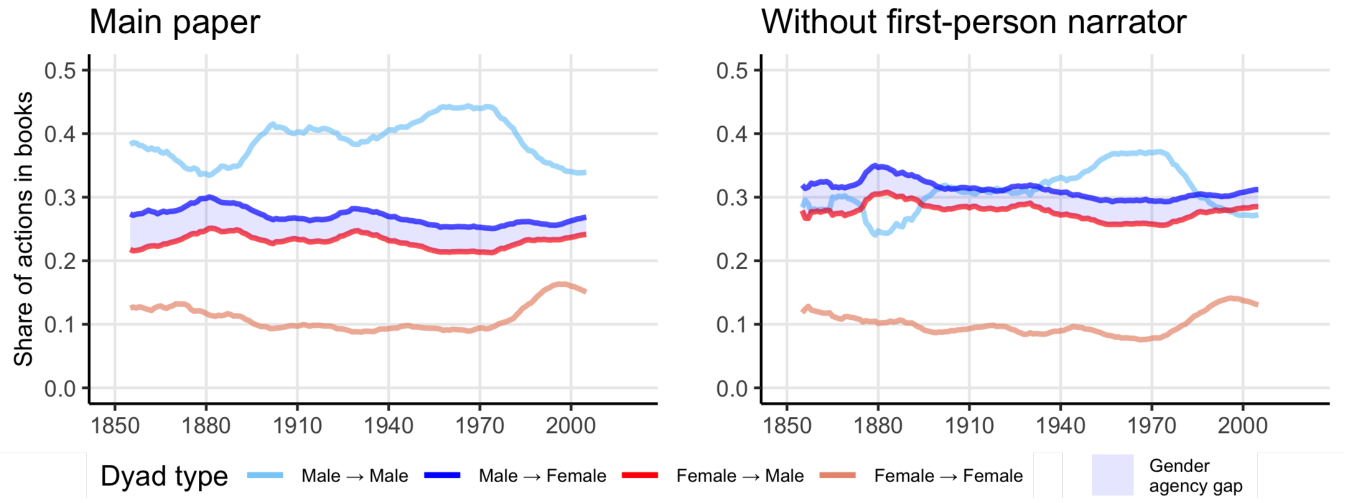


**Fig. S15.** Distribution of actions with all characters (main paper, left) and without first-person narrator (right).

In Figure S19, the evolution of the raw and importance-adjusted gender agency gap for the relationships not involving first-person narrators is shown. Beyond the fact that there is a gender agency gap for the entire period, two things are noteworthy: first, the gap is usually between one and two percentage points smaller. This indicates that at least some of the gender agency gap is attributable to the fact that there are more male first-person narrators. Second, the difference between the raw and the importance-adjusted gap is smaller. On the one hand, first-person narrators are responsible for most of the heterogeneity in degree, effective ego network size, and betweenness imbalances, for the narrative is usually (but not always) structured around them. However, beyond that, these results suggest that they are also largely responsible for the link between character importance and agency. Put differently, among non-first-person narrator characters, the link between characters' importance and their agency advantage is smaller. In this context, it is also worth noting that character gender prediction is somewhat less reliable for first-person narrators, a fact discussed in more detail in Appendix Section M.

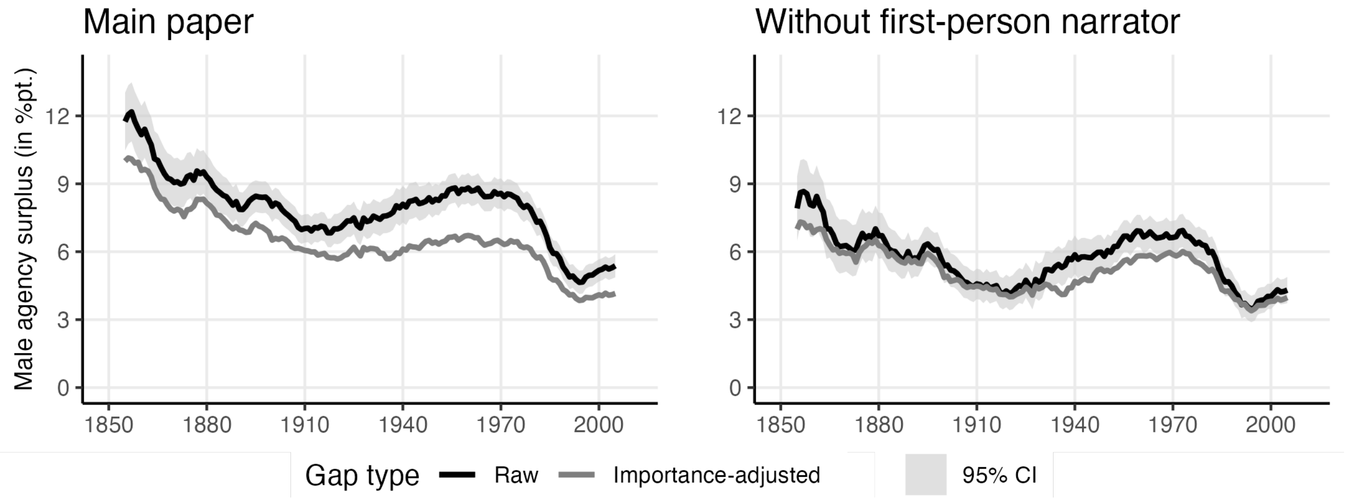


**Fig. S16.** Gender agency gap in the main paper (left) and without first-person narrators.

Figure S20 shows that the difference between male and female writers shrinks when disregarding first-person narrators. This

is because male-authored works are especially likely to have male first-person narrators. Note that 18.2% of the dyads in male-authored works involve a male first-person narrator, whereas only 13.1% of female-authored works do. In comparison, the difference between the prevalence of female first-person narrators is much smaller (4.6% and 6.9% of relationships in male- and female-authored works, respectively).

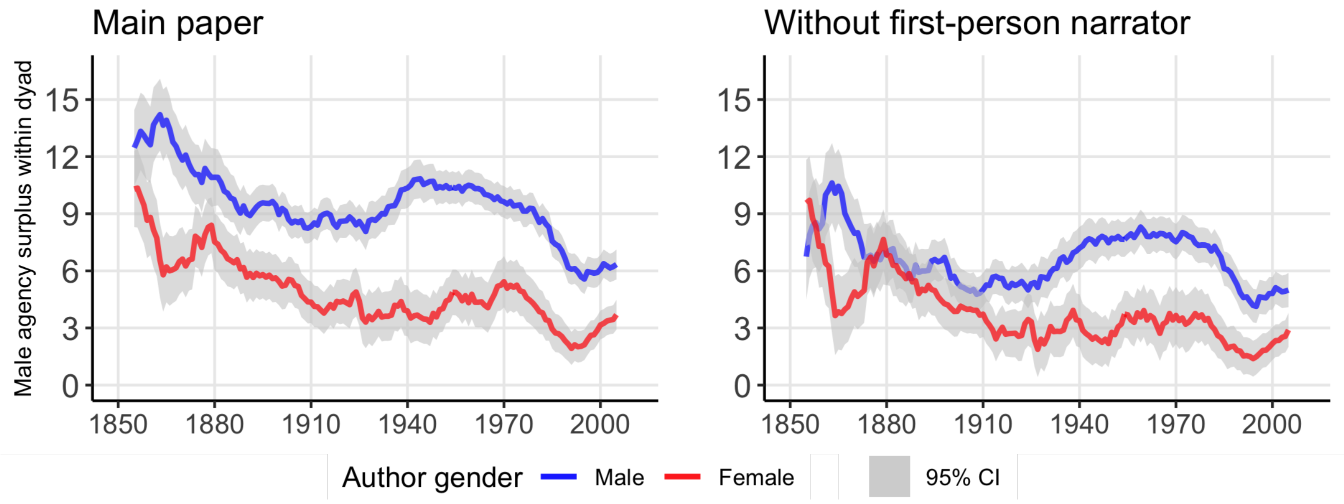


**Fig. S17.** Gender agency gap by author gender in the main paper (left) and without first-person narrators (right).

## K. Analyses based on variations of the action threshold

The analyses in the main paper are based on relationships with at least five exchanged actions, leading to a total of 568,302 cross-gender dyads. This threshold implies a focus on interaction among characters with substantial presence in a book. The decision to use a cutoff of five exchanged actions is motivated by the consideration that the analyses in this paper are premised on reliable identification of characters and predictions of character gender (see validation in Appendix Section M). Including characters that appear only in, say, one clause, would likely decrease the performance on these tasks.

Nonetheless, it is important to establish the robustness of thefindings to variations in this cutoff. To do this, using the USNC, I have generated character networks for each book for all possible action cutoffs between one and thirty. A cutoff of one means that all relationships among gendered characters are considered, even if they just contain one clause, leading to the inclusion of a considerably higher number of characters. Meanwhile, a higher cutoff implies a focus on "thicker" relationships, leading to the inclusion of fewer characters. In order to also present the character importance-adjusted gap, I then computed all three measures of character importance (degree, effective ego network size, and betweenness centrality) based on the new networks independently. In Figure S18, I present the gap in both its raw and importance-adjusted variant conditional on all thresholds. The gap for the USNC at the specification used in the main paper (i.e., a cutoff of 5 actions) lies at 7.7 (raw) and 5.9 percentage points (importance-adjusted). Overall, I find that the gap is smaller for lower action cutoffs, with values of 6.7 (raw) and 4.0 (adjusted) for a minimal threshold of one action. These numbers also imply that when including less important characters, imbalances in character importance account for a larger share of the gendered variation in agency. Conversely, when shifting the action threshold towards higher values, differences in character importance account for less variation.

While it may seem more comprehensive to include all relationships instead of only those with recurrent interaction into the analysis, there are two important limitations to these estimates. First, using all relationships irrespective of the action count arguably leads to a distortion: while character relationships with minimal interaction (e.g., a single clause) shape impressions of agency, they arguably do so less than relationships that are discussed repeatedly throughout a book. To address this, I calculate an additional version of the gap that weights dyads within a book by how many actions they contain. At the minimal cutoff, that is, when including all relationships, this produces estimates of 7.7 (raw) and 4.7 (importance-adjusted) for the gap.

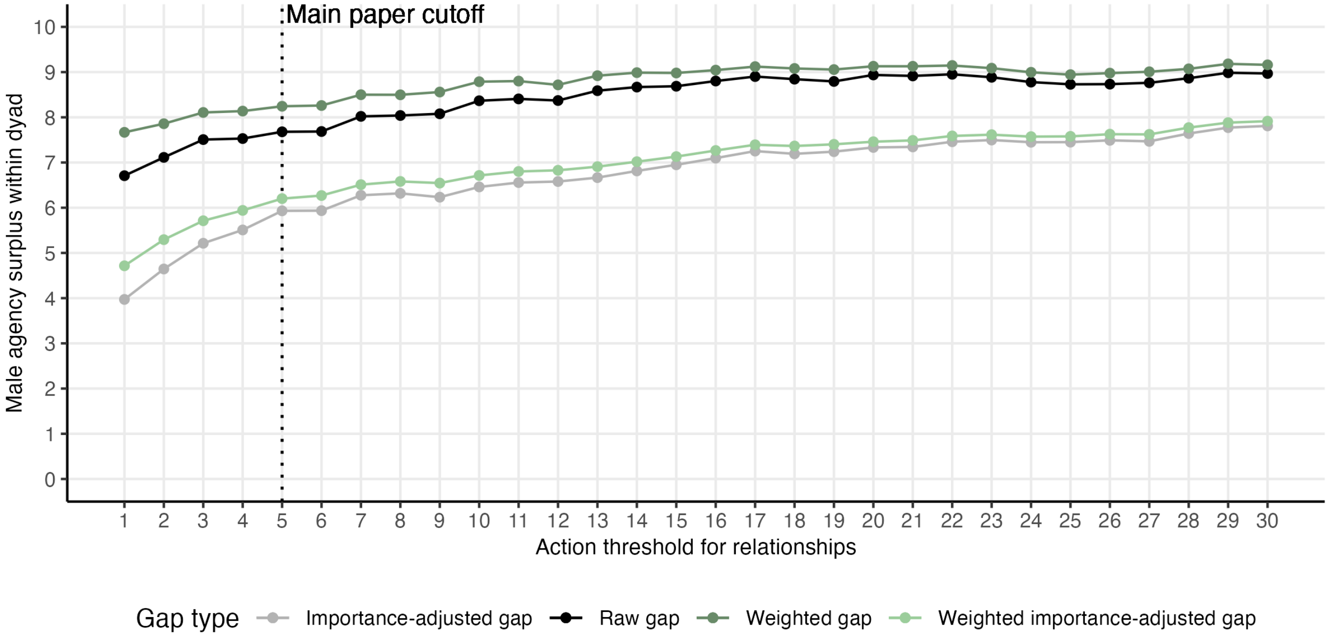


**Fig. S18.** Gender agency gap depending on cutoff used to define relationships. The dotted line indicates the specification used for the analyses in the main paper. Estimates in this figure are based on the USNC.

Second, as stated above, gender prediction is likely to be less accurate for characters that only make very short appearances. To test this systematically, I drew a stratified sample of 130 characters (10 from each decade) from the characters that were included in the analysis with the minimal threshold (i.e., an action cutoff of one). I then manually annotated the gender of these characters by looking at the original text (see Appendix Section M for the main validation). Accuracy for gender prediction in this sample was .915, which is lower than the .96 in the sample found in the main validation which is based on a cutoff of five. There were four cases of relationships for which gender could not be inferred. In two of these cases, the coreference resolution had erroneously grouped together entities of different genders and there was an even split; on another case, the

phrase "Cosmetics" was considered a character. Furthermore, there were two instances in which the character corresponded to the mention of an animal (a kitten and a horse). While these were indeed gendered and the gender prediction was correct, this may suggest that including entities with minimal appearances may amplify other conceptual challenges (no non-human entities were in the other validation steps based on the higher threshold).

Note that the accuracy estimate is based on a relatively small sample and getting precise estimates for the accuracy at different action cutoffs would require manual annotation of a significantly larger set of characters. Nonetheless, this suggests that including more characters likely increases the error rate for gender prediction and, potentially, coreference resolution. This prompts a new question: to what extent is the lower gap for lower thresholds a consequence of measurement error S18. While a complete answer would require having ground truth on all characters' gender, the question can be tackled by simulating the effects of a higher error rate on the gap estimate. To do this, I start from the data that is based on a five-action cutoff (i.e., the same specification as in the main paper). The estimated accuracy of gender prediction in this sample is .96 (see Appendix Section M). Based on this premise, I then flip the gender assignment of a random sample of characters to approximate different error rates. For instance, flipping the gender of 1.1% (rounded) of the characters approximates an accuracy of .95, flipping 2.2% approximates .94, flipping 3.3% approximates 9.3 and so forth. I then re-generate the networks and set of cross-gender dyads for these data with higher error rate. I repeat this process 20 times for a series of accuracy values between .87 and .95. While this approach does not account for ways in which gender prediction error may not be random, it allows us to generate a baseline estimate for the extent to which gender prediction error may affect the the size of the gap.

In S19, I present the gaps for these different simulated thresholds. The association between the estimated gap and the accuracy of gender prediction is nearly linear for both the raw gap and the importance-adjusted one. At an accuracy level of .91, I find that estimate for the raw gap closely align with that found previously for the analyses with the one-action cutoff (cross in S19) while the estimates for the importance-adjusted gap are, on average, around .8 percentage points higher than the observed gap for the one-action cutoff (triangle in S19). This suggests that the increased error rate for character gender likely does not fully explain the decrease of the importance-adjusted gap presented in Figure S18. Note that there there is uncertainty around the accuracy of gender prediction for the character sample based on a one-action relationship cutoff (the 90% confidence interval spans between .875 and .955). Nonetheless, based on these analyses, we can state that the gender agency gap, at least in the adjusted variant, is likely to be somewhat smaller for brief interactions than for "thicker" cross-gender relationships that span throughout a book.

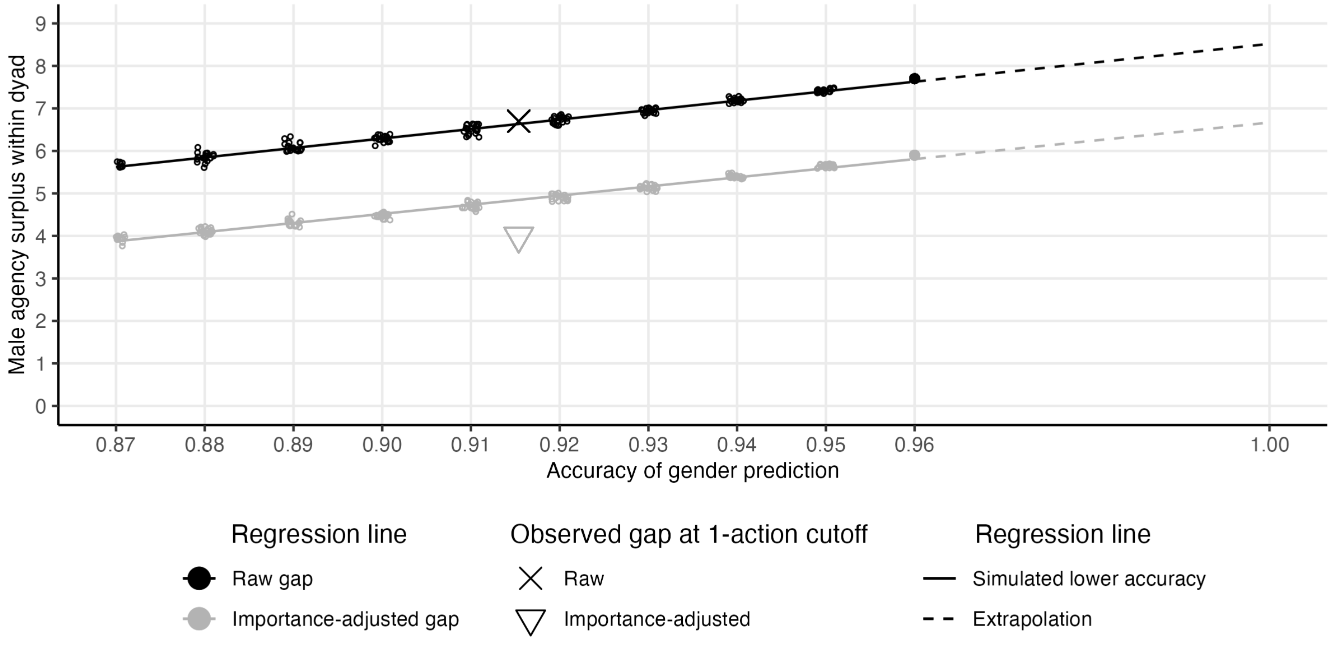


**Fig. S19.** Gender agency gap for different accuracy levels of character gender prediction. These estimates in this figure are based on the USNC. Specifically, the scenario with an action cutoff of five actions is used for this analysis, which is estimated to have a gender prediction accuracy of .96 (see Appendix Section M). Additional error is then introduced into these data by randomly flipping the gender of a fraction of the characters to match the accuracy levels shown on the x axis. Lower accuracy levels can be approximated by increasing the fraction of characters with flipped gender. This procedure is repeated 20 times for each accuracy level. For each of these 180 data sets, the gender agency gap is computed in both its raw and importance-adjusted variant. The empty points representing these estimates are jittered along the x axis to visualize heterogeneity. Subsequently, linear regression lines were fit through these points. The filled points represent the empirical gaps for the five-action cutoff scenario. The dotted line represents an extrapolation which estimates the expected gap for higher levels of gender prediction accuracy. The cross and the triangle represent the empirically observed gap for the one-action cutoff scenario (see Figure S18). it's x-coordinates are based on the estimate for the gender predication accuracy level of .915, which was obtained through manual annotation of a random sample of 130 characters.

Besides this, these analyses hold another important implication. The fact that there is most definitely measurement error in gender prediction for the main analyses together with the near-linear association between measurement error and the gap suggest that the true gap is most definitely slightly larger than reported in the main paper. Given the best estimate for the accuracy in the five-action cutoff scenario (which is .96, see Appendix Section M), we can expect the true gaps to be .8 (raw) and .7 (importance-adjusted) percentage points larger, both in the raw and importance adjusted variant. Therefore, as is noted in the main manuscript, the numbers presented there are a conservative estimate of the size of the gap.

Finally, in Figure S20, I present the shares of actions in different dyad types, also including within gender interaction for different action cutoffs. This is a synchronic replication of the analyses presented in Panel A of Figure 2, which is, again, based on the USNC (see Appendix Section F for a diachronic replication using the same action cutoff). Unlike the size of the gap, I find that the shares of within gender actions display little variation, especially across the lower action cutoff values. When focusing on highly dense relationships, the focus on cross gender actions increases relative to that of actions among male characters. This suggests that the most densely described relationships in fiction writing are those between male and female characters.

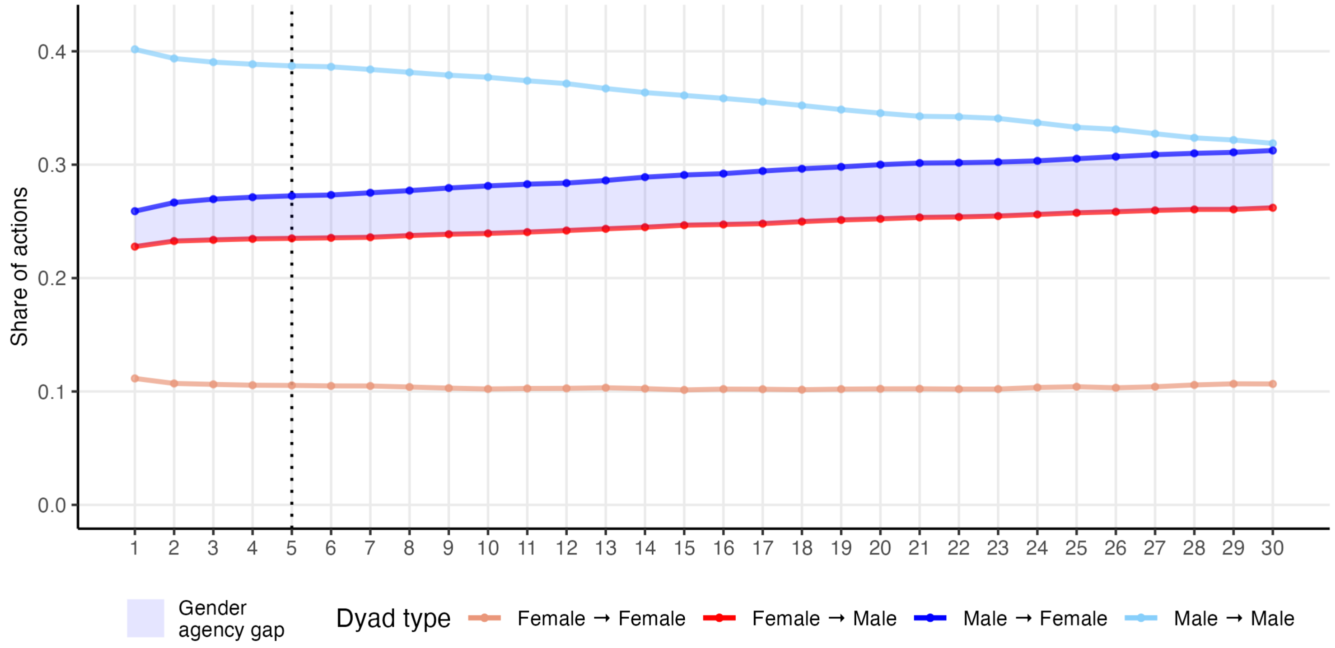


**Fig. S20.** Prevalence of actions in different dyad types among all actions for different cutoff used to define relationships. The dotted line indicates the specification used for the analyses in the main paper. Estimates in this figure are based on the USNC. Actions were averaged over books and replicate the analyses presented in Panel A of Figure 2 in the main paper.

## L. Using weighted verb representations for alternative measures of agency

As stated in the main paper, the notion of agency employed here is theoretically grounded in formal semantics, specifically, the literature on general thematic roles (17, 18). It is based on the idea that an agent associated with an event holds agency relative to the recipient. Among theorizations of agency, of which there is a long history (19–21), this position aligns most closely with Fuchs' (22) notion of agency as a concept of second order, that is, something which is attributed by an observer. This study does not investigate human interaction and can, therefore, largely bypass the complicated questions concerning free will and intentions that (depending on the conceptualization) can arise when seeking to attribute agency in interaction. Instead, we observe linguistic expressions in which, we premise, such attributions have been made by the author via the medium of syntax: "She kissed him." — if it says so on the page.

In the first two sections of the analyses, it is premised that syntax alone is sufficient for making such attributions. That is, agency is measured by looking at what character is more prone to the agent role rather than the recipient role in the transitive verbs (or pseudo-transitive verbs, e.g., think-of) associated with a given dyad—but we do not consider the semantics of these verbs. In the third section, we loosened this constraint in so far as we examined, among others, attributions of physical or communicative action. Here, I go one step further in this direction and offer additional, alternative measures of agency. These measures move us beyond the question of "who is the agent?" and towards the question of "what are their actions?"

This distinction roughly aligns with the two broad traditions of theorizing agency identified by Campbell (21). Whereas, the first conceives of agency simply as the human ability to initiate action, the second, concerns the efficacy of these actions in the world. This second form, labeled "agentic power," is defined as "power over either others or social structure more generally." (ibid. fn. 1) It is evident that when conceptualizing agency as agentic power, some verbs project agency more strongly than others (consider, for instance, "protect" versus "watch"), and some ("beg," "hide-from") may even be indicative of a relative lack thereof.

To create a measure of the distribution of agentic power within a dyad, I first draw on resources from Affect Control Theory (ACT) (23–25). ACT scholars have spent considerable effort measuring the affective meanings associated with a large variety of concepts. Among other things, this includes measuring the powerfulness (or "potency") that people associate with certain actions. This is usually done by confronting survey respondents with a concept and asking them to rate it along a scale with two poles labeled "powerless" to "powerful." Ratings of concepts are published as dictionaries. For instance, in a recently released dictionary that combines a series of surveys (26), "cure" is associated with a potency score of 3.63, making it the most powerful action. In contrast, the least powerful action is "beg" with a potency score of -2.0.

In ACT, scores like the above are used to simulate interactions between different identities and to assess how perplexing an interaction is in a specific cultural context. Here, I use the potency scores of actions to generate a dyadic measure of agentic power. First, I combine the dictionaries of Smith-Lovin et al. (26, 27), Heise (28), and Mostafavi et al. (29). I do this by giving preference to the dictionaries in the cited order, that is, I take all estimates of Smith-Lovin et al. (26) and add estimates of the other sources only if they are for additional actions. Note that the estimates of Mostafavi et al. are not derived via the classic surveys but instead estimated via a novel procedure that combines previous surveys with textual data. I take their estimates for actions only for which there is no survey-based estimate. Keeping only single-word actions (e.g., "beg") or ones followed by a preposition (e.g., "object-to"), this combination yields estimates for 1,100 actions. This makes it possible to match estimates for 50.3% of all actions exchanged within the main cross-gender dyads. The five actions with the highest potency, beyond cure, are the following: save (3.4), inspire (3.3), rescue (3.3), educate (3.3), and help (3.2). The least potency, beyond beg, is associated with mumble-to (-1.7), whine-to (-1.6), plead-with (-1.5), cling-to (-1.4), stutter-at (-1.3). Across the board, affective meanings tend to be relatively stable over time (30) though there are changes for particular concepts. This is mostly discussed in relation to identities (e.g., "gay"), but is, presumably to a lesser extent, also true for behaviors. Given that the analyses do not heavily depend on any particular action but stretch across a large variety of them, it appears defensible to use contemporary estimates of affective meaning.

To generate a comparable measure to that used in the main paper, I replace the number of all actions in a dyad with the sum total of all projected agency aggregated across all actions in a dyad. Verbs with negative values are assumed to project agency onto the recipient of said action. This means, for instance, that a female character attacking (1.4) the male character adds 1.4 to the female agency count, but a female character begging the male character (-2.0) will add 2.0 to the male count. Like in the original measure, I then generate the percentage-point surplus of male agency in a dyad, which varies between -100 (agency is exclusively projected onto the female character) and 100 (agency is exclusively projected onto the male character). A value of 0 indicates that agentic power is equally distributed between the male and the female character whereas positive values generally indicate a surplus in male agency. On the dyadic level, the resulting measure correlates at .68 with the count-based one used in the main paper.

Beyond the ACT-based measure, I replicate the analyses with a second agency dictionary that was recently publicly released by Antoniak and colleagues (31). This dictionary is based on work by Sap and colleagues (32) and assigns a score of -1, 1, or 0 to verbs based on whether they project agency onto the object, the subject, or neither. The paper defines agency as follows:

"The agency attributed to the agent of the verb denotes whether the action being described implies that the agent is powerful, decisive, and capable of pushing forward their own storyline." (p. 2330) This definition comes conceptually close to "agentic power" — yet it adds the elements "decisive" and "capable of pushing forward their own storyline." The overall quality of this dictionary is likely to be lower than the ACT one given that the annotation was done by only three independent coders. Meanwhile, ACT is an established research program and scores are based large scale surveys. That said, testing the results with an additional, independent resource adds nuance and robustness to the finding. Besides, the dictionary is almost twice as large (2,145), allowing me to score 65.3% of all verbs. Like above, I then compute the total amount of agency projected within a dyad by adding the verb scores and coding negative values as projecting agency onto the object. I then form a measures for the relative agency advantage bound between -100 and 100. At .53, the correlation for this measure with the count-based one is slightly lower than for the ACT-based one.

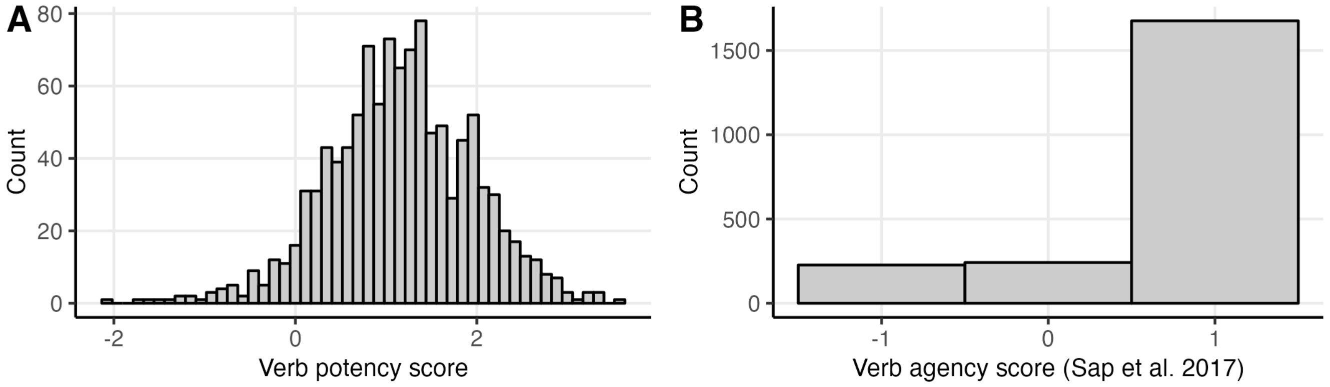


**Fig. S21.** Panel A show the potency score distribution of the 1,100 verbs with available potency scores from the different ACT dictionaries. Panel B shows the distribution of the agency scores for 2,145 verbs based on Sap et al.

In Figure S21, I show the distributions of the potency (ACT) and agency (Sap et al.) scores. Unsurprisingly, most scores are positive, following the intuition that verbs tend to connote agency on behalf of the subject and not the object. The mean values are 1.16 (ACT) and 0.68 (Sap et al.). Statistical properties of these distributions are documented in Table S7.

**Table S7. Statistical properties of verb agency scores**

| Measure | Mean | Median | Standard deviation | Minimum | Maximum | N |
|---|---|---|---|---|---|---|
| Potency (ACT) | 1.16 | 1.16 | .8 | -2.02 | 3.63 | 1100 |
| Agency (Sap et al. 2017) | .68 | 1 | .66 | -1 | 1 | 2,146 |

In Figure S22, I present the distributions of the three measures on the dyadic level. The measure based on the ACT-scores is smoother than the other two because these scores are continuous. The count-based score and the one based on the Sap et al. scores are more likely to take on particular values. For the same reason, agency parity is more likely for counts or discrete scores. Furthermore, more dyads have extreme values (-100 and 100), which is a consequence of the fact that not all verbs in the ≥5-action dyads could be matched to scores. The resulting sparsity leads to overall greater variance in the agency scores.

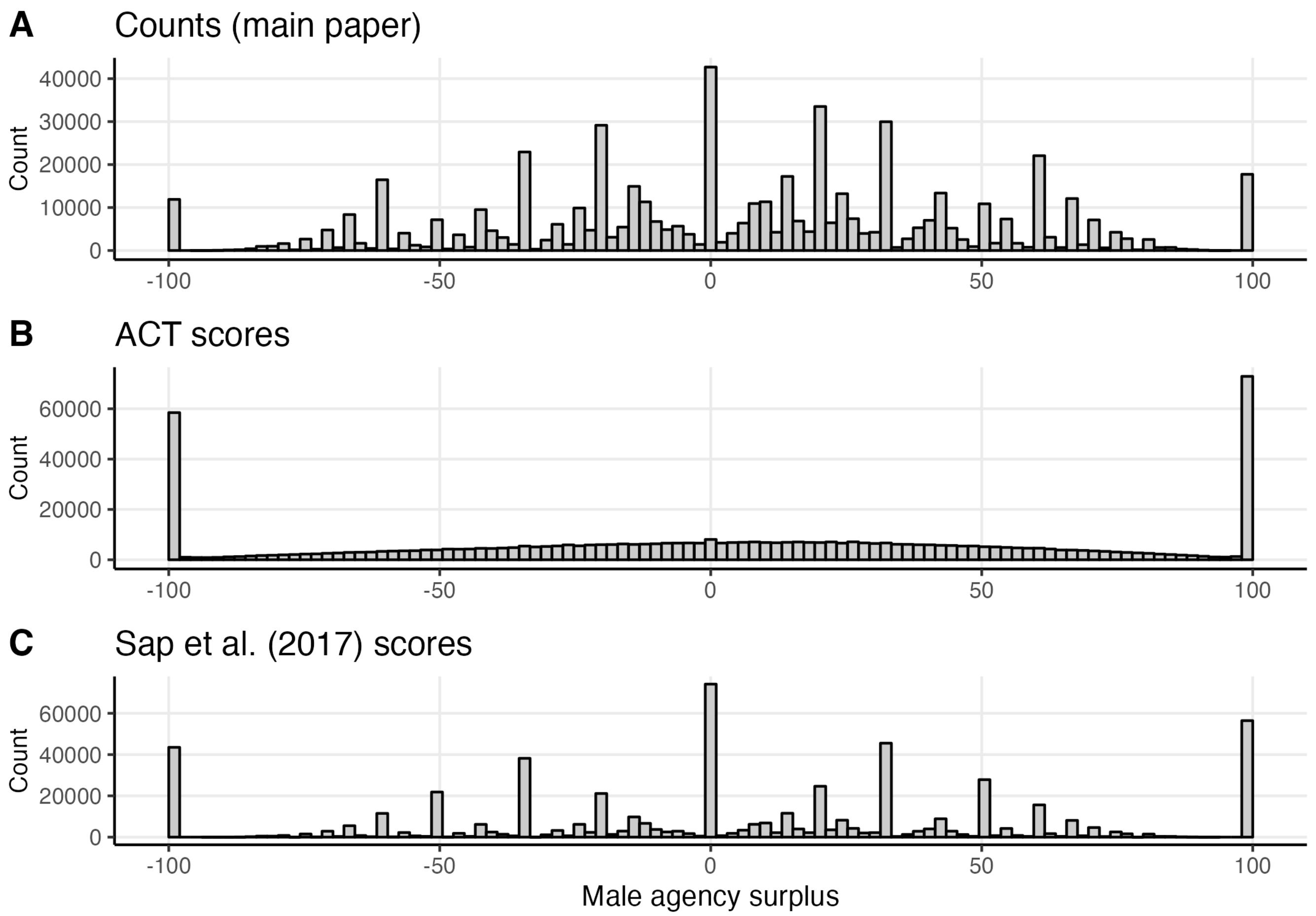


**Fig. S22.** The figure shows the distributions for the male agency surplus on the dyadic level based on three distinct measures. Panel A shows the count based measure used in the main paper. In this case, the measure represents the male percentage point advantage in terms of the share of actions sent within a cross-gender dyad. Panel B shows the weighted measure that uses ACT-based word scores. Panel C shows the measure based on the scores by Sap and colleagues (32). In this case, the total agency projected within a dyad is not equal to the number of actions but on the aggregation of the scores, where negative scores project agency onto the object of an action.

In Table S8 I present statistics of the three measures averaged over dyads within books. We see that the mean for both weighted measures is positive. This indicates that male characters aren't just more agentic in the sense that they are agents of most of the actions in cross-gender dyads, but that they possess more agentic power than their female counterparts (ACT) and that this is also the case for a weighted agency measure that incorporates elements of decisiveness and driving storylines (Sap et al.). In fact, the male agency advantage has roughly the same size when using weighted verb representations. While the measure based on the ACT scores is almost equal to the one based on the raw counts, the measure based on the Sap et al. scores is about one percentage point lower. The standard deviation for both weighted scores is higher than for the raw counts, which, again, is a consequence of the higher levels of sparsity in these measures as dyads based on fewer verbs are more likely to have more extreme agency distributions. Note that the minimal discrepancy in the agency advantage for the counts (7.39 versus 7.2 in reported the main paper) stems from the fact that here, I first average over dyads whereas in the main paper this number is based on averaging over actions within books.

**Table S8. Three measures for male agency advantage**

| Measure | Mean | Median | Standard deviation | Minimum | Maximum |
|---|---|---|---|---|---|
| Main paper (counts) | 7.39 | 7.4 | 27.3 | -100 | 100 |
| Agentic power (ACT scores) | 7.1 | 7.1 | 37.75 | -100 | 100 |
| Agency (Sap et al. 2017) | 6.35 | 6.33 | 32.91 | -100 | 100 |

In Figure S22 I compare the agency gap from the main paper with the two weighted measures. Like in the main paper, the values are positive for the entire time series. Furthermore, the trends for all measures are highly similar as there are two relatively sharp declines, one in the 19th century and one towards the end of the 20th. The correlation with the count-based measure across the time series is 0.97 (ACT) and .91 (Sap et al.).

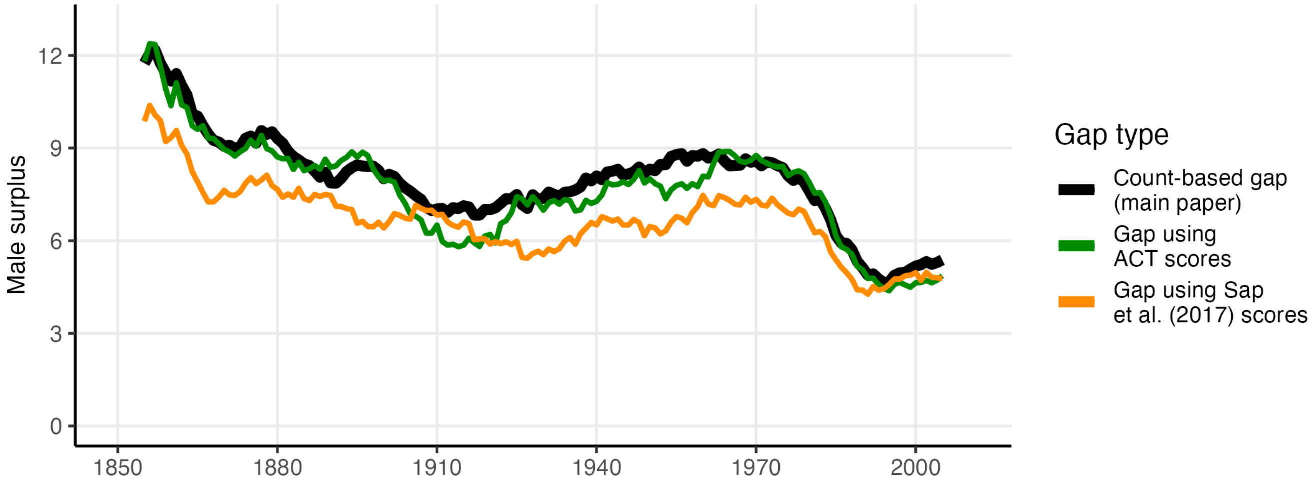


**Fig. S23.** Male agency surplus over time based on the count-based measure (main paper), the ACT-based measure, and the measure based on the verb scores of Sap et al. (32). The figure averages over books and replicates panel B of Figure 2 in the main paper. All measures range from -100 to 100. Positive values indicate that male characters have more agentic power. The measures are based 568,302 (count-based), 560,049 (ACT), and 564,612 (Sap et al.) dyads, reflecting the fact that not all verbs could be matches to scores leading some dyads to be dropped. All lines use 10-year moving averages.

## M. Validation of coreference resolution and character gender prediction

An important processing subtask underlying the analyses is coreference resolution. Characters are often not referenced via their proper name (e.g., "John Doe") but via other phrases — especially pronouns (e.g., "he," "him," "his") but also other noun phrases ("his friend"). The problem of coreference is, of course, not unique to literary texts. Yet literary texts can be especially challenging for coreference resolution, not least because they tend to be longer than most other text forms (33, 34). Coreference resolution models are commonly evaluated on the OntoNotes dataset (16). However, OntoNotes is mainly made up of news and conversation data and can therefore hardly be used for a reliable assessment of how well a model detects coreferences in literary texts. To make such evaluations possible, in what must have been a heroic annotation effort, Bamman and colleagues (35) recently created an annotated dataset of coreference in English literature. This dataset is an extension of LitBank (34) and currently the best available resource for assessing coreference resolution on literary texts. It contains text from 100 works of English language fiction published in the 18th, 19th, and 20th centuries and covers a variety of authors, styles, and genres — which makes its composition highly similar to that of the data used for the analyses. Coreference, here, is not just annotated among characters but also other entities (e.g., locations or vehicles).

The BookNLP pipeline used for data processing in my analysis was trained on a combination of PreCo (36) and LitBank. When evaluated against a held-out portion of LitBank, the model is reported to achieve an average F-score (the canonical measure for evaluating coreference systems) of 79.0 (for details on the dataset and measures, see Bamman et al. (35), for details on training and evaluation, see Bamman (37)). This is only slightly less than state-of-the-art models achieve on the OntoNotes datasets (38) and gives confidence in the model's general ability to resolve coreferences in English-language literature.

That said, while this evaluation of the model's performance is encouraging, for the analyses of this paper, I am technically only concerned with the model's performance in a very specific subset of instances. Namely, it matters whether the model can correctly resolve coreferences for main characters (i.e., ones that meet the relationship action frequency thresholds) when, and only when, interacting with other main characters across gender lines. This may be a simpler task than general coreference, for such characters, unlike side characters, usually have distinct names (i.e., "Lisa" instead of "the girl") and inferring coreference is likely easier when there is heterogeneity in pronouns in a clause (e.g., in "she kissed him" instead of "he kissed him."). However, since this is merely an intuition that lacks supporting hard evidence, I conducted additional evaluations.

For this, I first sampled 25 characters, evenly distributed throughout time. For each of these characters, I then examined all instances in which they were involved in an action either as agent or as recipient, which led to a total of 530 actions. Like for the analyses, I only considered actions that occurred in relationships between characters of different genders that have at least five actions in them. Thus, each of the sampled characters is associated with at least five actions in the sample while one character had as many 99 actions associated with it. For each instance of an action, I annotated whether the agent or recipient identified with the character was actually the respective character or not. Imagine, for instance, that we sample Character-1 and we encounter among her actions "kissing," stemming from the sentence "[Lisa] kissed him." In other words, the model has mapped the token "Lisa" onto Character-1 in the respective sentence. In cases like this, where the sentence involves a proper name, it is usually relatively easy to infer whether the mapping is correct, for one can simply look at the most frequent proper nouns used to refer to the respective character. If, in this case, the character is normally referred to as "Lisa," the mapping is coded as correct. At other times, however, one encounters sentences like "she kissed him" or "the girl kissed him." In such cases, it is not possible to infer whether the instance is correct based on the sentence alone. Therefore, I read and explored the context of the respective sentence. Sometimes, the sentence immediately before or after was sufficient to infer the correctness of the mapping. Often, however, it was necessary to investigate multiple passages before and after an action to infer whether the mapping was correct. Because of this, U used the U.S. Novel corpus for the coreference validation where one could easily access the full text.

Table S9 presents the results of the evaluation. I find that the coreference for the sampled character is correct in .92 of the 530 examined actions. Importantly, I find no major differences between genders, as the expected share of correct attributions is .92 for male and female characters alike. Rather than systematically biasing the analyses in the sense of attributing more actions to characters of one gender, imperfect coreference resolution introduces error into the analyses. While this form of error may minimally attenuate the measure of agency imbalance in relationships, overall, these numbers suggest that the coreference underlying the analyses is solid. The file with the coreference annotations will be made available in the supplementary material.

**Table S9. Character coreference resolution evaluation**

| | N actions | N characters | Share correct | Share correct (averaged over characters) |
|---|---|---|---|---|
| Total sample | 530 | 25 | .92 | .92 |
| Male subsample | 208 | 10 | .89 | .92 |
| Female subsample | 322 | 15 | .94 | .92 |

The BookNLP pipeline also makes character gender predictions. This is done based on a combination of three pieces of

information: the gender scaling of given names according to government records, the alignment of character names with gendered honorifics (e.g., "Mr. Pargiter"), as well as pronominal information obtained through coreference (i.e., characters referenced with male or female pronouns can be assumed to be male or female, respectively). The pipeline's capacity to predict character gender has been evaluated on an earlier version of the Hathi corpus used here by Underwood and colleagues, who sampled and manually annotated the gender of 525 characters. They report precision values of .95 and .91 for identifying female and male characters, respectively; recall values are reported at .83 and .86, respectively (39). These values remain constant over the time studied here and do not decline as we go further back in time (ibid., p. 15). This is important, for a change in the capacity to accurately predict character gender would bias the estimates of the distribution of agency between genders.

These numbers provide a stable reference point for our analyses because the corpus we use is largely the same. In fact, one might expect even better performance of the model in the context of this paper's analyses for two reasons. First, Underwood and colleagues used a prior version of the BookNLP pipeline which did not yet make use of transformer models. The pipeline has since been adapted and is now based on BERT. This means that its performance on subtasks that precede and underlie gender prediction (e.g., parsing) is improved, which is likely to lead to improvements in the latter. Second, the performance reported by Underwood and colleagues is based on a random sample of characters. However, in my analyses I only consider characters that appear in at least one dyad with at least 5 exchanged actions, and most of the characters are involved in considerably more actions and dyads. In other words, I focus on characters that are central to the novel and appear frequently, which implies that the model has more data points (i.e., references to the character via pronouns) to build on when making gender predictions.

Nonetheless, because character gender is crucial for the dependent variable in this paper, I conducted an additional validation. For this, I again used the USNC where one could easily access the full text. I drew a random sample of 120 characters evenly distributed throughout time, each coming from a different book. I then manually annotated these characters' genders. In most cases, a character's referential gender could be determined based on the sentences around actions used in the analyses. In some cases, I read through more sections of the book to determine the gender of a character. The annotated files are provided in the supplementary materials. As expected, I find slightly better performance than Underwood and colleagues (39), with an overall accuracy of .96. Recall is at .94 and .97 for female and male characters, respectively, and precision at .96 for both (see Table S10). Like Underwood and colleagues, I find no indication that gender prediction is less reliable in older works (the erroneous predictions come from the years 1916, 1970, 1983, 1986, and 1994).

**Table S10. Character gender prediction evaluation.**

| | Accuracy | Recall | | Precision | | N | | |
|---|---|---|---|---|---|---|---|---|
| | | Male | Female | Female | Female | Total | Male | Female |
| Character random sample | .96 | .94 | .97 | .96 | .96 | 120 | 74 | 47 |
| 1880s sample | .96 | .96 | .96 | .96 | .96 | 60 (10 from initial sample) | 32 | 28 |
| First-person narrator sample | .86 | .97 | .5 | .86 | .86 | 52 (2 from initial sample) | 39 | 13 |

In addition to this general assessment, I conducted two additional checks. First, despite the above and despite Underwood and colleagues' finding of constant accuracy over time, I wanted to double-check whether gender prediction works equally well for older books. Since the BookNLP was mostly trained on a corpus containing 18th to 20th century works (34) it should have relatively constant performance over time. Nonetheless, I extra caution seems warranted because working with older texts is often delicate due to factors that reduce the overall quality of the data (e.g., OCR problems). I sampled an additional 50 characters from the 1880s, this being the earliest decade in the USNC. Again, I find no indication that gender prediction works less well in older texts, with accuracy, precision, and recall at .96 for both genders in the 1880s sample.

Second, as has been noted by Underwood and colleagues (39), gender prediction is harder for first-person narrators, for the respective pronouns (I, me, my) do not imply any gender. Note, however, that it is by no means impossible for BookNLP to infer the gender of a first-person narrator, for instance through instances where the narrator is addressed in direct speech ("'How are you doing, John?', he asked me."). Furthermore, unlike other characters, such narrators are often a constant presence throughout the book, which means that there are many potentially useful data points. I drew a sample of 50 first-person narrators, which are identified by BookNLP with the '0' code. I then combined this sample with the first-person narrators included in the larger random sample from above. In the annotation process, I noticed that in some books, the narrator code was assigned to regular characters who happened to use first-person pronouns in direct speech. In cases where the '0' entity contained direct speech from multiple characters, I assigned the gender of whichever character was primarily associated with the entity. Not surprisingly, I find worse performance for predicting the gender of first-person narrators (documented in Table S10), with an overall accuracy of .86. Recall is at .5 and .97 for female and male characters, respectively; precision at .86 for both. This implies that there is a slight tendency to overpredict male characters among first-person narrators. Nonetheless, these numbers appear sufficient to warrant the inclusion of first-person narrators in the main analyses. This is especially so because the agency of (usually male) first-person narrators is one part of the very concept that the analysis aims to measure. Merely excluding first-person narrators would undermine this effort. That said, as is shown in Appendix Section J, the general finding of an agency gap is robust to the exclusion of first-person narrators. While the gender agency gap decreases when

first-person narrators are removed (see Figure S19), this is primarily because male characters are, in fact, overrepresented among first-person narrators, who typically carry agency advantages: I find 75% of the first-person narrators in the manually labeled sample to be male, while only 61.1% of the character sample is.

## N. Measures for character importance

As stated in the main manuscript, I operationalize the extent to which a work focuses on a character with three conceptually distinct measures. First, character *degree* is measured as the number of actions a character is involved in within all main relationships of a book. Formally, if $a_{ij}$ represents the number of actions exchanged between characters $i$ and $j$, then the degree $D$ of character $i$ is defined as:

$$D_i = \sum_j a_{ij} \qquad [3]$$

Second, I measure the *effective size* of a characters ego network. This measure was first proposed by Burt (40) as an operationalization of the notion of sturctural holes. Specifically, the measure captures the extent to which a node connects other nodes, taking into account the potential redundancy of its ties by accounting for ties between alters. In the case at hand, the measure is used to implement the idea that a character is more important if it is positioned in a structural hole of the character network, that is, the character serves as a bridge connecting different relatively unconnected parts of the story's social structure. While the effective size of an ego network can be defined for directed and/or weighted graphs, I use this measure on the undirected and unweighted character network. Note that the directed variant would effectively build notions of agency into the measure of importance, and that the volume of action is captured by the degree (see above). Borgatti (41) has demonstrated that for undirected, unweighted graphs, Burt's definition of the effective size of a characters ego network $E_i$, can be re-written as:

$$E_i = n_i - \frac{2t_i}{n_i} \qquad [4]$$

where $n_i$ is the size of character $i$'s ego network (that is the number of characters $i$ is tied to) and $t_i$ is the number of ties between alters in said network. This measure is "local" insofar as it is based on a character's neighborhood but does not account for the global character network.

To address this, I use a measure for the betweenness centrality of a character, implements the idea that a character is more relevant if it is placed at the center of a book's entire social structure. The betweenness centrality $C$ of a character $i$ is defined as:

$$C_i = \sum_{j \neq i \neq s} \frac{\sigma_{js}(i)}{\sigma_{js}} \qquad [5]$$

where $\sigma_{st}$ is the total number of shortest paths from character $j$ to node $s$, and $\sigma_{js}(i)$ is the number of those paths that pass through character $i$. Betweenness centrality was implemented using the igraph R package (42).

Subsequently, these attributes are used to create three measures for the relative difference in character importance within a given dyad. Given the degrees of a male character ($D_m$) and a female character ($D_f$) character, the male degree surplus $D_{mf}$ within a cross-gender dyad is defined as:

$$D_{mf} = 200(\frac{D_m}{D_m + D_f} - .5) \qquad [6]$$

The corresponding dyadic measures for the male surplus in effective ego network size and betweenness centrality are analogously defined. In the latter case, a small constant is added to the betweenness centrality of characters to avoid division by 0 in cases where both nodes don't lie on any geodescis. Note that the other transforming constants are used to align the scales of the different measures. Positive values indicate that the male character is more important, while negative values indicate the opposite. A value of 0 implies equal importance of the male and female character. The asymptotic minimum and maximum values for all three measures are -100 and 100. Average values for the three measures are 3.1 (degree), 4.3 (effective ego network size) and 5.4 (betweenness centrality), implying that male characters are more important accoring to each of them. Figure S24 shows the histogram of all three measures. Note that many characters don't lie on any geodesics, which leads to the trimodal distribution in Panel C. The relatively high number of 0 values for both degree and effective ego network size imply equal importance of the male and female character. Some of these (though not all of them) stem from cases in which a cross-gender dyad is either unconnected to the rest of the books social structure or the only relationship in a book which passes the threshold of 5 exchanged actions. Table S11 shows statistical properties of the three variables. Unsurprisingly, the three dyadic measures of advantages in importance are highly correlated, indicating that, if a character has an advantage in terms of degree, it is also highly likely to have an advantage in terms of betweenness or its effective ego network size and vice versa. Still, these measures capture distinct notions of how a character may be importance.

**Table S11. Relative character importance measures**

| Measure | Mean | Standard deviation | Minimum | Maximum | Correlation with degree | Correlation with betweenness |
|---|---|---|---|---|---|---|
| Character degree | 3.14 | 58 | -99.1 | 98.92 | 1 | .9 |
| Effective ego network size | 4.28 | 54.21 | -89.84 | 90 | .94 | .95 |
| Betweenness centrality | 5.43 | 84.75 | -100 | 100 | .9 | 1 |

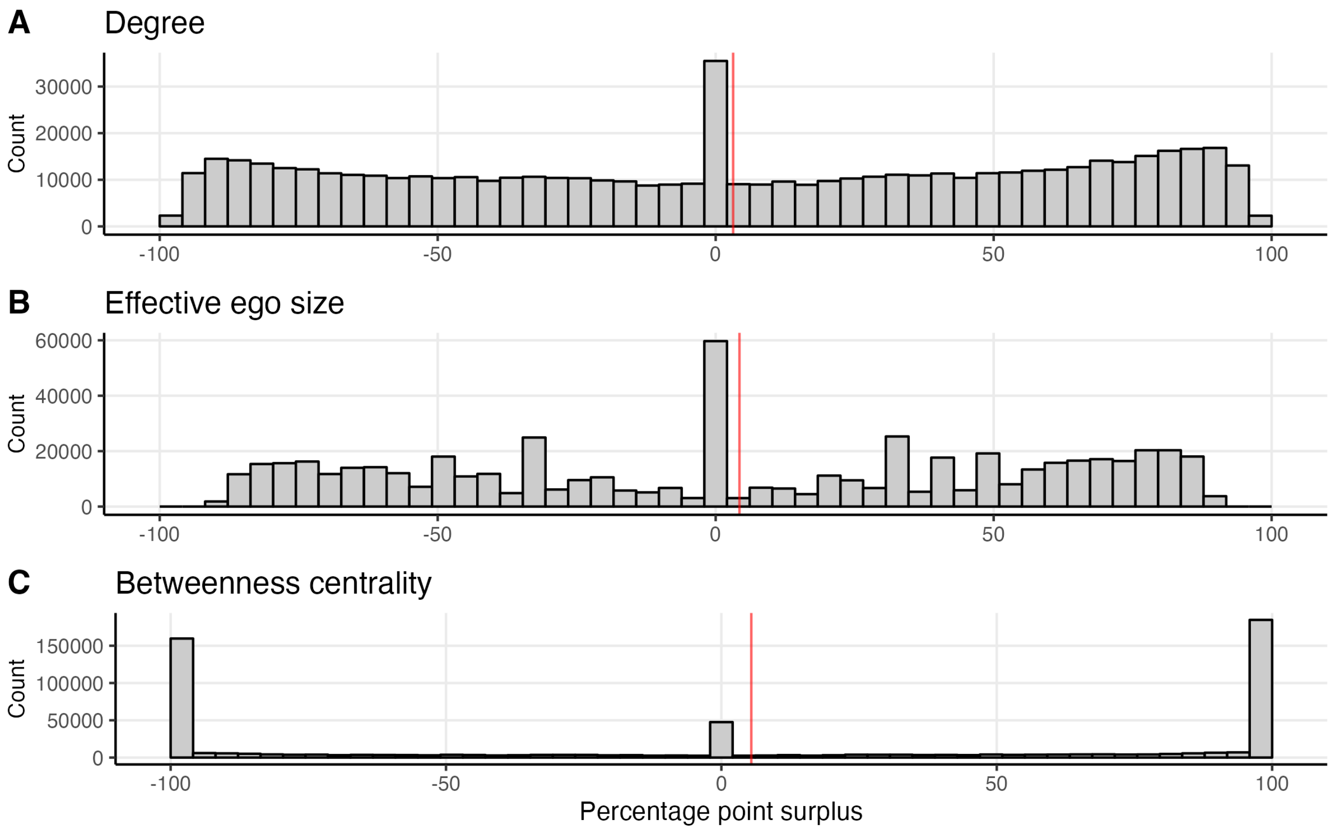


**Fig. S24.** The figure shows the distributions of the dyadic measures of relative character importance. Positive values indicate that the male character has a greater degree, effective ego network size, or betweenness centrality, while negative values indicate the opposite. The red lines represent averages. All figures are based on the 568,302 cross-gender relationships that were used for the main analyses in the paper.

## O. Distribution of reference form by character gender

While not the primary focus of the paper, Panel A of Figure 2 also presents the shares of within gender actions, that is, female→female actions and male→male actions. In a recent contribution, it was shown that interaction among male entities is less prevalent than interaction among female ones (3). However, male-male actions make up a significantly larger share of the actions in the present study when compared to the respective paper. The previous study did not detect such narrators, but used three elements to identify gendered entities: gendered pronouns, two lists of gendered first names, and words preceded by honorifics like "Mr.," "Mrs." Much of the comparatively higher share of male→male actions in the present study is explained by the fact that the previous study did not identify first-person narrators, among which there is a disproportional overrepresentation of male characters (see Appendix Sections J and M). However, as Appendix Section J, even when removing first-person narrators, the share of male-male actions is still larger than in the previous study.

Engagement with the text suggests that this larger share is likely the case because female characters are often referenced by their first name while male characters are much more frequently referenced by their last name. Note, also, that the previous contribution used a list of 2.964 female first names but only 767 male first names. This followed the rationale that in the U.S. population, there is considerably less heterogeneity in male first names than there is in female ones, and these lists would account for an equal share of the actual male and female population (see Appendix E of (3)). However, whether this also holds true for entities in fiction writing is technically a different question and was not independently examined.

To test this hypothesis systematically, I drew a random sample of 500 character mentions in sentences containing actions. I then manually annotated the type of phrase or word that referenced the character. For this sample, I excluded actions from first-person narrators, for it would seem redundant to include them. To ensure comparability, I used the USNC corpus, which was used in the respective study. I distinguish the following reference types:

- Third person pronouns (he, him, his, she, her, hers). These would have been *detected* in (3).
- First and second person pronouns (I, me, you), which mostly stem from direct speech. These would have been *undetected* in the previous study.
- First names contained in the lists used in the previous study.
- Names that are not contained in the lists used in the previous study. Note that it is not always possible to distinguish whether something is a first or a last name. So this category includes references via things that are clearly last names as well as names that are rare first names.
- Composites of first and last name. Depending on context, these may have been detected in the previous study.
- Phrases with honorifics that would have been matched in the previous study (e.g., "Mr. Gatsby").
- Phrases that would have gone unmatches in the previous study (e.g., "the soldier")

In Table S12, I present the shares for each category by character gender. I find that male characters are indeed about five times as likely to be referenced by an unmatched name, that is, either by their last name or by an unmatched name. Cumulatively, I estimate that among non-first person narrators, the previous contribution missed around half of the referenced to male characters while only missing 30% of the references to female characters. Note also that for the detection of interaction *within* gender, these errors scale quadratically because both characters need to be identified.

**Table S12. Reference form by character gender**

| Character gender | 3rd person pronoun | 1st & 2nd person pronoun | Matched first name | Unmatched name | First & last name | Phrases with honorifics | Unmatched phrases | Detected in (3) |
|---|---|---|---|---|---|---|---|---|
| Female | .46 | .19 | .23 | .04 | .01 | .02 | .06 | .70 |
| Male | .36 | .19 | .14 | .22 | .02 | .01 | .05 | .52 |

## P. Within relationship change of gender agency gap

In this section, I test whether the gender agency gap typically increases or decreases over the course of books. To do so, I drew a random sample of 20,000 cross-gender relationships from the USNC data (using 5-action and 10-action cutoffs for relationships). Sampling considerably reduces the computational cost and is sufficient for this purpose. For each of these relationships, I then computed the correlation between and actions token position within a book and whether the action was a male→female (1) or female→male (0) one. Dyads in which only one character acts were removed from the analysis, as they have no variation. In Figure S25, I present the distribution of these correlations. For both cutoffs, the mean is .00. This implies that in the average dyad, there is no association between the gender direction of an action and when the action occurs. Note that token position here is a proxy for narrative time, but that the relationship between these concepts is usually not linear (43). The standard deviation in both distributions indicates that in most relationships, there is some "back and forth" — i.e., actions sequences overlap, and are not neatly split (which would imply values of 0 or 1). The standard deviation for the 10-action cutoff is smaller due to the fact that with more actions, perfect splits of the action sequence become less likely.

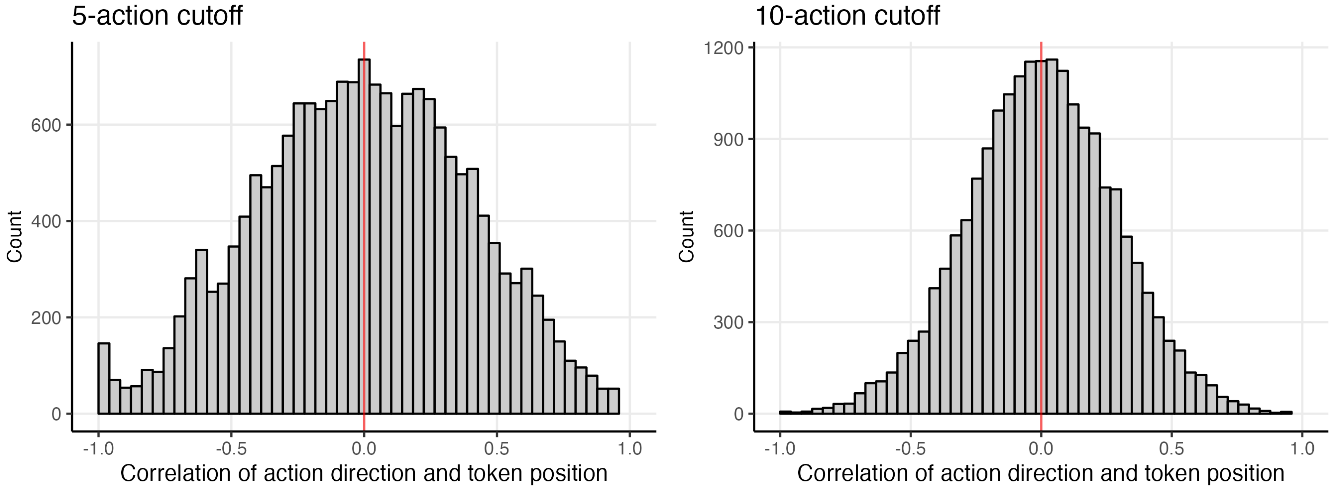


**Fig. S25.** The figure shows the distributions of the correlation between an action verbs token position within the book and whether whether the action was a male→female (1) or female→male (0) one. A positive correlation would indicate, that the male→female actions occur towards the end of the book whereas a negative correlation indicates that the female→male occur towards the end of the book. The mean of both distributions is 0. Both figures are based on a sample of 20,000 cross-gender relationships.

## Q. Gender role conformity within dyads

My measure for the gender role conformity is based in a straightforward intuition: how predictable is the gender assignment of the actions contained in a cross-gender dyad, given what we know about the genderedness of actions? To operationalize this notion, I first compute the conditional probabilities of all actions $a$ for both directions $d$ based on all cross-gender dyads. For instance, for the action of "nursing" I generate the probabilities $p(nurse \mid m \rightarrow f)$ and $p(nurse \mid f \rightarrow m)$. For each action, I then use these two probabilities to form the terms $p(m \rightarrow f \mid nurse)$ and $p(f \rightarrow m \mid nurse)$. For the case of "nursing", for instance $p(m \rightarrow f) = .18$ and $p(f \rightarrow m) = .82$. Note that because I base these terms in actions' probabilities conditional on direction, they are independent of the fact that there are more male→female than female→male actions. Subsequently, I define the role conformity of a dyad $R$ as the average likelihood of its actions' observed directions:

$$R = \frac{1}{N}\sum_{i=1}^{N} p(d_i \mid a_i), \quad d_i \in \{m \rightarrow f, f \rightarrow m\} \qquad [7]$$

where $N$ is the total number of actions within that dyad, $d_i$ the observed direction of the $i$th action $a_i$, and $p(d_i \mid action_i)$ the probability of observing the direction given the action. Intuitively, this measure captures how well a randomly drawn action from a dyad predicts which character is female and which one is male. A value closer to 1 indicates that the gender of the actions participants (who is male and who is female) is well predicted, a value close to .5 means that the gender of the participants cannot be predicted based on the action content, and a value close to 0 would indicate that the relationship is subversive in terms of its action content. The advantage of averaging the probabilities is that the measure becomes formally independent of the number of actions exchanged within the dyad. While one could base a measure on a cumulative gender prediction of the direction that makes use of all actions in a dyad, this measure would likely be correlated with the number of actions. The measure defined above is uncorrelated with the number of actions in a dyad ($r = .02$). Note, also that the models presented below additionally control for the number of actions in a dyad. It is worth pointing out that authors, of course, have other ways of describing relationships in gender role conforming ways. Yet focusing on the directed action content in this way provides a formally simple and intuitive approach.

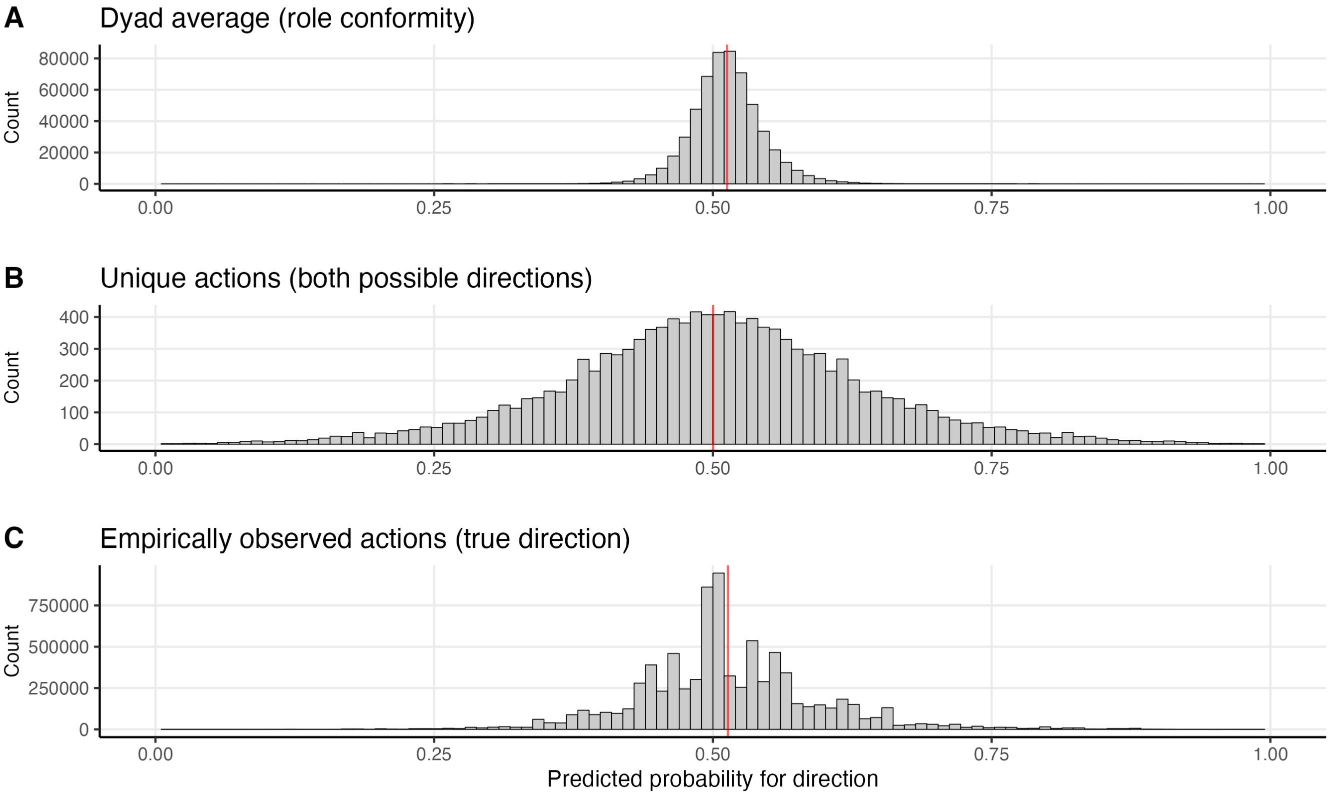


**Fig. S26.** Role conformity based on actions. Panel A presents the distribution of the dyadic measure for role conformity. Panel B shows the distributions of the probabilities for all unique actions for both directions. Panel C shows the likelihood of all 8.3 million empirically observed actions' true directions. These measured are based on using only actions that have at least 100 total occurrences.

I Figure S26 I present the distribution of the dyadic measure for role conformity (Panel A), together with the distributions of the probabilities for all unique actions for both directions (Panel B), and the likelihood of all empirically observed actions'

directions (Panel C). Note that the Panel B has a wider spread than the other two distributions and is symmetric with a mean of .5 because each action (e.g., "nurse") contributes two values. We see that it's standard deviation is larger than that of Panel C. This is because many highly frequent actions have relatively equal gender valences. Note that the mean of distribution C is only slightly above .5 (.51). This means that observing a random action in a cross-gender dyad is typically not sufficient for inferring the gender direction once we condition on the different agency levels as I did above. Put differently, when considering the size of the gap presented in the main paper, for a *random* cross-gender action, the direction of the action tells us more about which character is male or female than the action content alone would. It follows that the mean for the role conformity in Panel A is also only slightly above .5 (.51). Note that for this measure, I only considered actions which appear at least 100-times in cross-gender dyads, which preserves 98.2% of all observed action instances. Given this high percentage, the statistical properties of the dyadic measure are robust to variation of this threshold (see S13).

In Table S14 I present the results of regressions models predicting the male agency surplus within dyads. The model architecture follows that of the main analyses presented in Appendix Section B and Model 2 in the table is the same as the full model (Model 7) presented in Table S2 in Appendix Section B. The full model controls for variation over time nonparametrically via a decade fixed effects. As reported in the main paper, a one-standard deviation increase in a dyad's role conformity is associated with a 5.7 percentage point increase in the male agency surplus ($p < .001$).

Finally, it is worth pointing out that gender roles have evolved over time, and that some actions may have shifted in their gender valence. To accommodate this fact in my measure, I compute an additional version of the measure, for which I re-compute all $p(a \mid m \rightarrow f)$ and $p(a \mid f \rightarrow m)$ terms for the periods between 1850 and 1900, 1901 and 1950, and 1951 and 2010. For each dyad, I then compute $R$ based on the time window the work was written in. The resulting measure correlates at $r = .89$ with the role conformity measure that is based on global action probabilities. As Model 4 in Table S14 shows, the effect of this alternative measure is very close to the one based on global action probabilities.

**Table S13. Dyadic role conformity measures**

| Measure | Mean | Standard deviation | Median | Minimum | Maximum | N of dyads |
|---|---|---|---|---|---|---|
| Dyadic role conformity (10-occurrence-threshold) | .51 | .03 | .51 | .26 | .85 | 568,301 |
| Dyadic role conformity (100-occurrence-threshold) | .51 | .03 | .51 | .26 | .78 | 568,298 |
| Dyadic role conformity (1000-occurrence-threshold) | .51 | .03 | .51 | .2 | .79 | 568,228 |

**Table S14. Regression models with dyadic gender role conformity**

| | Male agency surplus | | | |
|---|---|---|---|---|
| | (1) | (2) | (3) | (4) |
| Male degree surplus | | $-0.021^{***}$ | $-0.020^{***}$ | $-0.019^{***}$ |
| | | (0.003) | (0.003) | (0.003) |
| Male betweenness centrality surplus | | $-0.024^{***}$ | $-0.021^{***}$ | $-0.024^{***}$ |
| | | (0.003) | (0.003) | (0.003) |
| Male effective ego size surplus | | $0.175^{***}$ | $0.165^{***}$ | $0.173^{***}$ |
| | | (0.005) | (0.005) | (0.005) |
| Logged # of actions (z-transformed) | $0.649^{***}$ | $0.702^{***}$ | $0.751^{***}$ | $0.522^{***}$ |
| | (0.050) | (0.055) | (0.078) | (0.055) |
| Role conformity (z-transformed) | $6.080^{***}$ | | $5.731^{***}$ | |
| | (0.079) | | (0.085) | |
| Male author | | $1.685^{***}$ | $1.535^{***}$ | $1.644^{***}$ |
| | | (0.184) | (0.178) | (0.180) |
| Feminist author | | $-3.899^{*}$ | $-3.971^{*}$ | $-3.943^{*}$ |
| | | (1.892) | (1.807) | (1.812) |
| Role conformity by period (z-transformed) | | | | $5.378^{***}$ |
| | | | | (0.089) |
| Constant | $6.654^{***}$ | $9.886^{***}$ | $7.302^{***}$ | $8.618^{***}$ |
| | (0.081) | (0.636) | (0.635) | (0.611) |
| Decade fixed effects | No | Yes | Yes | |
| Pseudo R2 (marginal) | 0.02 | 0.03 | 0.04 | 0.04 |
| Pseudo R2 (conditional) | 0.28 | 0.28 | 0.27 | 0.27 |
| Number of authors | 40677 | 30480 | 30480 | 30215 |
| Number of books | 87530 | 72546 | 72546 | 71396 |
| Number of male characters | 333328 | 281758 | 281757 | 277231 |
| Number of female characters | 297245 | 250713 | 250710 | 246784 |
| N | 568,298 | 486,180 | 486,177 | 478,343 |

$^{*}p < .05$; $^{**}p < .01$; $^{***}p < .001$

## References


1. J. D. Choi, M. Palmer, *Guidelines for the Clear Style Constituent to Dependency Conversion* (UC Boulder, Institute of Cognitive Science), (2012).
2. M. Honnibal, I. Montani, S. Van Landeghem, A. Boyd, SpaCy: Industrial-strength natural language processing in python (https://doi.org/10.5281/zenodo.1212303) (2023) Accessed 11/01/2023.
3. O. Stuhler, Who does what to whom? Making text parsers work for sociological inquiry. *Sociol. Methods Res.* **51**, 1580–1633 (2022).
4. O. Stuhler, semgram: Extracting semantic motifs from textual data (https://CRAN.R-project.org/package=semgram) (2022) R package version 0.1.0.
5. D. Bates, M. Mächler, B. Bolker, S. Walker, Fitting linear mixed-effects models using lme4. *J. Stat. Softw.* **67**, 1–48 (2015).
6. J. Pustejovsky, sandwich: Robust covariance matrix estimators (https://cran.r-project.org/web/packages/sandwich/index.html) (2023) R package version 0.5.10.
7. S. Nakagawa, P. C. D. Johnson, H. Schielzeth, The coefficient of determination r(2) and intra-class correlation coefficient from generalized linear mixed-effects models revisited and expanded. *J R Soc Interface* **14** (2017).
8. T. Underwood, P. Kimutis, J. Witte, Noveltm datasets for English-language fiction, 1700-2009. *J. Cult. Anal.* **5** (2020).
9. Chicago-Text-Lab, U.S. Novel Corpus (https://textual-optics-lab.uchicago.edu) (2023) Accessed 11/01/2023.
10. R. So, H. Long, Y. Zhu, Race, writing, and computation: Racial difference and the US novel, 1880-2000. *J. Cult. Anal.* **3** (2019).
11. J. Pruett, Machine learning for genre classification (https://textual-optics-lab.blogspot.com/2020/07/machine-learning-for-genre.html) (2020) Accessed 11/01/2023.
12. N. Baym, *A Guide to Novels by and about Women in America, 1820-70.* (Cornell University Press, Ithaca, NY), (1973).
13. N. Armstrong, *Desire and Domestic Fiction: A Political History of the Novel.* (Oxford University Press, Oxford), (1989).
14. G. Brown, *Domestic Individualism: Imagining Self In Nineteenth-Century America.* (University of California Press, Berkeley, CA), (1992).
15. C. Blevins, L. Mullen, Jane, John ... Leslie? A historical method for algorithmic gender prediction. *Digit. Humanit. Q.* **9** (2015).
16. E. Hovy, M. Marcus, M. Palmer, L. Ramshaw, R. Weischedel, Ontonotes: The 90 *Proc. Hum. Lang. Technol. Conf. North Am. Chapter ACL* p. 57–60 (2006).
17. A. R. Davis, *3. Thematic roles*, eds. C. Maienborn, K. von Heusinger, P. Portner. (De Gruyter Mouton, Berlin, Boston), pp. 99–125 (2019).
18. A. Williams, *Arguments in Syntax and Semantics.* (Cambridge University Press, Cambridge), (2015).
19. M. Emirbayer, A. Mische, What is agency? *Am. J. Sociol.* **103**, 962–1023 (1998).
20. S. Hitlin, G. H. Elder, Time, self, and the curiously abstract concept of agency. *Sociol. Theory* **25**, 170–190 (2007).
21. C. Campbell, Distinguishing the power of agency from agentic power: A note on Weber and the "Black Box" of personal agency. *Sociol. Theory* **27**, 407 (2009).
22. S. Fuchs, Beyond agency. *Sociol. Theory* **19**, 24–40 (2001).
23. D. R. Heise, *Understanding events: Affect and the construction of social action.* (Cambridge University Press, Cambridge), (1979).
24. D. R. Heise, *Expressive Order. Confirming Sentiments in Social Actions.* (Springer, New York, NY), (2007).
25. L. Smith-Lovin, Behavior settings and impressions formed from social scenarios. *Soc. Psychol. Q.* **42**, 31–43 (1979).
26. L. Smith-Lovin, et al., Mean affective ratings of 929 identities, 814 behaviors, and 660 modifiers by university of georgia and duke university undergraduates and by community members in durham, nc, in 2012-2014. (https://affectcontroltheory.org/usa-combined-surveyor-dictionary-2015/) (2020) Accessed 11/01/2023.
27. L. Smith-Lovin, D. T. Robinson, B. C. Cannon, B. H. Curdy, J. H. Morgan, Mean affective ratings of 968 identities, 853 behaviors, and 660 modifiers by amazon mechanical turk workers in 2015. *https://affectcontroltheory.org/usa-online-dictionary-2015/* (Accessed 11/01/2023).
28. D. R. Heise, Project magellan: Collecting cross-cultural affective meanings via the internet. *Electron. J. Sociol.* **5** (2001).
29. M. Mostafavi, M. D. Porter, D. T. Robinson, Learning affective meanings that derives the social behavior using bidirectional encoder representations from transformers. *arXiv:2202.00065* (Accessed 11/01/2023).
30. N. J. MacKinnon, A. Luke, Changes in identity attitudes as reflections of social and cultural change. *The Can. J. Sociol.* **27**, 299–338 (2002).
31. M. Antoniak, et al., Riveter: Measuring power and social dynamics between entities (http://maartensap.com/pdfs/antoniak2023riveter.pdf) (2023) Accessed 11/01/2023.
32. M. Sap, M. C. Prasettio, A. Holtzman, H. Rashkin, Y. Choi, Connotation frames of power and agency in modern films in *Proceedings of the 2017 Conference on Empirical Methods in Natural Language Processing*, eds. M. Palmer, R. Hwa, S. Riedel. (Association for Computational Linguistics, Copenhagen, Denmark), pp. 2329–2334 (2017).
33. I. Roesiger, S. Schulz, N. Reiter, Towards coreference for literary text: Analyzing domain-specific phenomena in *Proceedings of the Second Joint SIGHUM Workshop on Computational Linguistics for Cultural Heritage, Social Sciences, Humanities and Literature*, eds. B. Alex, et al. (Association for Computational Linguistics, Santa Fe, New Mexico), pp. 129–138 (2018).

34. D. Bamman, S. Popat, S. Shen, An annotated dataset of literary entities in *Proceedings of the 2019 Conference of the North American Chapter of the Association for Computational Linguistics: Human Language Technologies, Volume 1 (Long and Short Papers)*, eds. J. Burstein, C. Doran, T. Solorio. (Association for Computational Linguistics, Minneapolis, Minnesota), pp. 2138–2144 (2019).
35. D. Bamman, O. Lewke, A. Mansoor, An annotated dataset of coreference in English literature in *Proceedings of the Twelfth Language Resources and Evaluation Conference*, eds. N. Calzolari, et al. (European Language Resources Association, Marseille, France), pp. 44–54 (2020).
36. H. Chen, Z. Fan, H. Lu, A. Yuille, S. Rong, PreCo: A large-scale dataset in preschool vocabulary for coreference resolution in *Proceedings of the 2018 Conference on Empirical Methods in Natural Language Processing*, eds. E. Riloff, D. Chiang, J. Hockenmaier, J. Tsujii. (Association for Computational Linguistics, Brussels, Belgium), pp. 172–181 (2018).
37. D. Bamman, BookNLP (https://github.com/booknlp/booknlp) (2023) Accessed 11/01/2023.
38. B. Bohnet, C. Alberti, M. Collins, Coreference resolution through a seq2seq transition-based system. *Transactions Assoc. for Comput. Linguist.* **11**, 212–226 (2023).
39. T. Underwood, D. Bamman, S. Lee, The transformation of gender in English-language fiction. *J. Cult. Anal.* **3** (2018).
40. R. S. Burt, *Structural Holes: The Social Structure of Competition.* (Harvard University Press, Cambridge, MA), (1995).
41. S. P. Borgatti, Structural holes: Unpacking Burt's redundancy measures. *Connections* **20**, 35–38 (1997).
42. G. Csárdi, et al., igraph: Network analysis and visualization in r (https://CRAN.R-project.org/package=igraph) (2022) R package version 2.0.3.
43. S. A. Yeager, Time maps: Theory and method. *J. Cult. Anal.* **8** (2023).

**Lists of feminist fiction writers.**

1. fairygoodboss.com (2022): https://fairygodboss.com/career-topics/feminist-writers (Accessed 07/11/2023)
2. becomeawritertoday.com (2023): https://becomeawritertoday.com/feminist-authors/ (Accessed 07/11/2023)
3. bustle.com (2016): https://www.bustle.com/articles/148815-10-feminist-fiction-writers-every-woman-should-read (Accessed 07/11/2023)
4. theguardian.com (2016): https://www.theguardian.com/books/booksblog/2016/apr/06/10-inspiring-female-writers-you-need-to-read (Accessed 07/11/2023)
5. literaryladiesguide.com (2018): https://www.literaryladiesguide.com/literary-musings/12-classic-feminist-authors-to-discover-or-rediscover/ (Accessed 07/11/2023)
6. thoughtco.com (2017): https://www.thoughtco.com/must-read-feminist-authors-739724 (Accessed 07/11/2023)
7. vocal.media (2022): https://vocal.media/viva/famous-feminist-writers-of-the-20th-century (Accessed 07/11/2023)
